# Discovery and functional characterization of neuropeptides in crinoid echinoderms

**DOI:** 10.3389/fnins.2022.1006594

**Published:** 2022-12-13

**Authors:** Alessandra Aleotti, Iain C. Wilkie, Luis A. Yañez-Guerra, Giacomo Gattoni, Tahshin A. Rahman, Richard F. Wademan, Zakaryya Ahmad, Deyana A. Ivanova, Dean C. Semmens, Jérôme Delroisse, Weigang Cai, Esther Odekunle, Michaela Egertová, Cinzia Ferrario, Michela Sugni, Francesco Bonasoro, Maurice R. Elphick

**Affiliations:** ^1^Department of Environmental Science and Policy, University of Milan, Milan, Italy; ^2^School of Biological & Behavioural Sciences, Queen Mary University of London, London, United Kingdom; ^3^Institute of Biodiversity, Animal Health and Comparative Medicine, University of Glasgow, Glasgow, United Kingdom

**Keywords:** neuropeptide, crinoid, echinoderm, SALMFamide, calcitonin, vasopressin/oxytocin, *Antedon mediterranea*, feather star

## Abstract

Neuropeptides are one of the largest and most diverse families of signaling molecules in animals and, accordingly, they regulate many physiological processes and behaviors. Genome and transcriptome sequencing has enabled the identification of genes encoding neuropeptide precursor proteins in species from a growing variety of taxa, including bilaterian and non-bilaterian animals. Of particular interest are deuterostome invertebrates such as the phylum Echinodermata, which occupies a phylogenetic position that has facilitated reconstruction of the evolution of neuropeptide signaling systems in Bilateria. However, our knowledge of neuropeptide signaling in echinoderms is largely based on bioinformatic and experimental analysis of eleutherozoans—Asterozoa (starfish and brittle stars) and Echinozoa (sea urchins and sea cucumbers). Little is known about neuropeptide signaling in crinoids (feather stars and sea lilies), which are a sister clade to the Eleutherozoa. Therefore, we have analyzed transcriptome/genome sequence data from three feather star species, *Anneissia japonica, Antedon mediterranea*, and *Florometra serratissima*, to produce the first comprehensive identification of neuropeptide precursors in crinoids. These include representatives of bilaterian neuropeptide precursor families and several predicted crinoid neuropeptide precursors. Using *A. mediterranea* as an experimental model, we have investigated the expression of selected neuropeptides in larvae (doliolaria), post-metamorphic pentacrinoids and adults, providing new insights into the cellular architecture of crinoid nervous systems. Thus, using mRNA *in situ* hybridization F-type SALMFamide precursor transcripts were revealed in a previously undescribed population of peptidergic cells located dorso-laterally in doliolaria. Furthermore, using immunohistochemistry a calcitonin-type neuropeptide was revealed in the aboral nerve center, circumoral nerve ring and oral tube feet in pentacrinoids and in the ectoneural and entoneural compartments of the nervous system in adults. Moreover, functional analysis of a vasopressin/oxytocin-type neuropeptide (crinotocin), which is expressed in the brachial nerve of the arms in *A. mediterranea*, revealed that this peptide causes a dose-dependent change in the mechanical behavior of arm preparations *in vitro*—the first reported biological action of a neuropeptide in a crinoid. In conclusion, our findings provide new perspectives on neuropeptide signaling in echinoderms and the foundations for further exploration of neuropeptide expression/function in crinoids as a sister clade to eleutherozoan echinoderms.

## Introduction

Neuronal secretion of peptides that act as intercellular signaling molecules (neuropeptides) is an evolutionarily ancient characteristic of nervous systems, which is reflected in the diversity of neuropeptides that have been discovered in bilaterian and non-bilaterian phyla (Elphick et al., 2018). Furthermore, evidence of a pre-metazoan origin of neuropeptide signaling has been reported (Yañez-Guerra et al., 2022). Neuropeptides are derived from larger precursor proteins that have an N-terminal signal peptide, which targets these molecules to the lumen of the endoplasmic reticulum for secretion. The precursor proteins can comprise one or more neuropeptides, which are bounded by monobasic or dibasic cleavage sites. Furthermore, during neuropeptide precursor processing post-translational modifications of neuropeptides can occur, which include most commonly the conversion of a C-terminal glycine residue to an amide group that is protective against carboxypeptidases. Other post-translational modifications of neuropeptides include conversion of an N-terminal glutamine to pyroglutamate, which is protective against aminopeptidases, tyrosine sulfation and intramolecular and/or intermolecular formation of disulphide bridges between cysteine residues (Abraham and Podell, 1981; Dockray, 1987). Neuropeptides typically exert effects on other cells by binding to specific G-protein coupled receptors (GPCRs), acting locally as modulators of synaptic transmission and/or systemically as hormones. The actions of neuropeptides at a cellular level then manifest at the organ/organismal level to cause changes in physiological processes and/or behavior. Thus, neuropeptides are important regulators of, for example, feeding and digestion, osmoregulation, growth, locomotor activity and reproductive processes (Jekely et al., 2018).

Investigation of the evolution of neuropeptide signaling systems has been greatly facilitated by transcriptome/genome sequencing. Initially, this was restricted to widely studied “model” species such as humans, mice, the nematode *Caenorhabditis elegans* and the insect *Drosophila melanogaster*, with comparison and functional characterization of neuropeptide signaling systems in these species providing key insights into neuropeptide relationships and the evolutionary origins of different neuropeptide types (Li et al., 1999; Hewes and Taghert, 2001; Hauser et al., 2006; Schoofs et al., 2017; Jekely et al., 2018). However, as transcriptome/genome sequencing has been applied to an ever-growing wider variety of animal taxa, important new insights into neuropeptide evolution have been obtained (Jekely, 2013; Mirabeau and Joly, 2013; Elphick et al., 2018). One of the animal phyla that has been important for reconstruction of the evolutionary history of neuropeptide signaling systems is the phylum Echinodermata (e.g., starfish, brittle stars, sea urchins, sea cucumbers, feather stars). As ambulacrarian deuterostomes, echinoderms, together with hemichordates, are positioned in a sister clade to the phylum Chordata (vertebrates, urochordates, and cephalochordates) and therefore they provide a key evolutionary link between research on neuropeptide systems in protostome invertebrates (e.g., arthropods, nematodes, mollusks, and annelids) and vertebrates (Semmens and Elphick, 2017; Elphick, 2020). Visualization of neuropeptide expression in echinoderm nervous systems was first enabled by use of antibodies to neuropeptides discovered in other phyla (e.g., the molluscan cardioactive peptide FMRFamide) (Elphick et al., 1989; Garcia-Arraras et al., 1991; Hoekstra et al., 2012). More recently, insights into the neuropeptide repertoire of echinoderms were enabled by sequencing of the transcriptome/genome of the sea urchin *Strongylocentrotus purpuratus* (class Echinoidea) (Burke et al., 2006; Rowe and Elphick, 2012; Monroe et al., 2018). Subsequently, analysis of transcriptome/genome sequence data has enabled discovery of neuropeptide precursor genes in other echinoderms, including sea cucumbers (class Holothuroidea), starfish (class Asteroidea), and brittle stars (class Ophiuroidea) (Rowe et al., 2014; Semmens et al., 2016; Smith et al., 2017; Zandawala et al., 2017; Suwansa-Ard et al., 2018; Chen et al., 2019). Furthermore, molecular characterization of neuropeptide signaling systems in selected echinoderm species has provided important insights into neuropeptide relationships and neuropeptide evolution (Semmens and Elphick, 2017). For example, the discovery of corazonin-type signaling in the starfish *Asterias rubens* and in other echinoderms revealed the urbilaterian origin of a neuropeptide system that hitherto had been thought to be unique to arthropods or protostomes (Tian et al., 2016). Similarly, the discovery of both somatostatin-type and allatostatin-C-type neuropeptides in *A. rubens* and other echinoderms revealed that neuropeptides that hitherto were thought to be orthologs are in fact paralogs (Zhang et al., 2022). Additionally, functional characterization of neuropeptides in *A. rubens* and other echinoderms has revealed evolutionarily ancient and conserved roles in regulation of, for example, feeding processes and reproduction (Kato et al., 2009; Mita et al., 2009; Tinoco et al., 2018, 2021; Odekunle et al., 2019).

Whilst much has been learnt in recent years about neuropeptide signaling systems in echinoderms, there remains one echinoderm class that has received little attention—the Crinoidea (feather stars and sea lilies). Crinoids are of particular interest for evolutionary studies on echinoderms because they are a sister clade to the sub-phylum Eleutherozoa, which comprises the Asterozoa (Asteroidea and Ophiuroidea) and Echinozoa (Echinoidea and Holothuroidea) (Telford et al., 2014). Therefore, comparative analysis of crinoids and eleutherozoans may facilitate reconstruction of the neuropeptide systems that existed in the common ancestor of extant echinoderms. Furthermore, anatomical studies have investigated crinoid neuroarchitecture and revealed homology between different regions of crinoid and eleutherozoan nervous systems (Heinzeller, 1998; Bohn and Heinzeller, 1999; Nakano et al., 2009; Mashanov et al., 2016; Ben Khadra et al., 2018; Mercurio et al., 2019). In the lecithotrophic doliolaria larvae of feather stars, the nervous system comprises an anterior apical organ and a diffuse basi-epidermal nerve plexus (Nakano et al., 2009; Barbaglio et al., 2012; Mercurio et al., 2019). The larval nervous system degenerates at metamorphosis and new neural cell populations develop in the post-metamorphic pentacrinoid stage (Nakano et al., 2009). On the oral surface neurons are found associated with the tube feet, while on the aboral surface the aboral nerve center (ANC) forms at the base of the calyx (Mercurio et al., 2019; Omori et al., 2020). The ANC projects aborally into the stalk, forming the stalk nerve, and orally into five thin nerves (Mercurio et al., 2019). In the adult, the oral nervous system comprises ectoneural and hyponeural systems that are homologous to the corresponding components in eleutherozoans. A third conspicuous component of the nervous system in crinoids is the aboral entoneural system, which comprises the ANC and brachial nerves running within the ossicles of each arm. However, there are very few studies that have investigated neuropeptide signaling systems in crinoids. Using antibodies to the starfish SALMFamide neuropeptides S1 and S2 (Elphick et al., 1991a), the presence and location of related neuropeptides in the feather stars *Antedon bifida* and *Antedon mediterranea* have been revealed (Heinzeller and Welsch, 1994; Bonasoro et al., 1995; Candia Carnevali et al., 1998). Furthermore, analysis of transcriptome sequence data obtained from *A. mediterranea* enabled identification of a transcript encoding a precursor protein comprising 14 SALMFamide-type neuropeptides (Elphick et al., 2015). However, more comprehensive analyses of transcriptome/genome sequence data to identify neuropeptide precursor genes have yet to be reported for crinoid species. Therefore, here we have analyzed transcriptome/genome sequence data obtained from three feather star species, *A. mediterranea, Florometra serratissima*, and *Anneissia japonica*, to produce a detailed inventory of crinoid neuropeptide precursors. Furthermore, informed by these findings, we have employed mRNA *in situ* hybridization and immunohistochemical techniques to investigate the expression of selected neuropeptides (F-type SALMFamide, calcitonin-type, vasopressin/oxytocin-type) in the larval (doliolaria), pentacrinoid and adult stages of *A. mediterranea.* Lastly, we have investigated the *in vitro* pharmacological actions of a vasopressin/oxytocin-type neuropeptide (crinotocin) on arm preparations from adult *A. mediterranea.* Collectively, our findings provide the first extensive account of neuropeptides in crinoid echinoderms and a strong basis for further studies on this fascinating group of marine animals.

## Materials and methods

### Crinoid transcriptome/genome sequence data analyzed

Transcriptome sequence data (Illumina HiSeq) for the feather star *A. mediterranea* were generated by the Elphick group at Queen Mary University of London^1^ and an assembly of these data generated previously using SOAPdenovo-Trans-31mer (Elphick et al., 2015) was analyzed here.

Transcriptome sequence data (Illumina HiSeq) for the feather star *F. serratissima* were generated by the Lowe group at Stanford University.^2^ These data were downloaded as fastq raw reads files^3^ and processed to remove potential adaptor sequences using Trimmomatic v. 0.36 (Bolger et al., 2014) with the parameters: ILLUMINACLIP:TruSeq3-PE-2.fa:2:30:10 SLIDINGWINDOW:5:5 MINLEN:30. A *de novo* assembly of the transcriptome data was generated using trinity/2.11.0 (Grabherr et al., 2011; Haas et al., 2013) with default parameters for paired-end reads.

The *A. mediterranea* and *F. serratissima* transcriptomes were translated into amino acid sequences using TransDecoder v. 5.5.0^4^ with the minimum sequence length set to 75 amino acid residues.

The genome sequence of the feather star *A. japonica* has been reported recently (Li et al., 2020). The proteins and translated CDS from genomic fasta files were downloaded from NCBI (GenBank GCA_011630105.1). The proteins file was used to identify putative neuropeptide precursors, while the translated CDS file was used for gene structure analysis.

To remove redundant transcript variants, all three proteomes were subjected to a cd-hit with an identity threshold of 96% (Li and Godzik, 2006; Fu et al., 2012). Both the non-cd-hit and the cd-hit versions of all three species proteomes were assessed for completeness using BUSCO v. 4.0.6 (Manni et al., 2021) (see Supplementary Table 1). The cd-hit versions for all three species were used for further analyses.

### Discovery of crinoid homologs of neuropeptide precursors identified in other echinoderms

To identify putative neuropeptide precursors in the three crinoid species, two different approaches were used.

First, a sequence-similarity search was performed against the crinoid proteomes using the amino acid sequences of already known neuropeptide precursors from several species of echinoderms as queries, including the brittle stars *Ophionotus victoriae* and *Amphiura filiformis* (Zandawala et al., 2017), the starfish *A. rubens* (Semmens et al., 2016) and *Acanthaster planci* (Smith et al., 2017), the sea cucumber *Apostichopus japonicus* (Chen et al., 2019) and the sea urchin *S. purpuratus* (Rowe and Elphick, 2012; Monroe et al., 2018). BLASTP was conducted with high E-value threshold of 0.1 because neuropeptide precursors are relatively short (e.g., typically 50–150 residues) and often exhibit lower levels of interphyletic sequence similarity than other types of proteins (Mirabeau and Joly, 2013).

Second, the cd-hit proteomes of all three crinoid species were run through PrediSi tool (Hiller et al., 2004) to retain only sequences with a predicted signal peptide. These were then further filtered to retain only sequences containing key features of neuropeptide precursors by using the Patternsearch.sh script developed by Thiel et al. (2021).^5^

The outputs of BLASTP and PrediSi+Patternsearch were then pooled and cd-hit with identity 100% was performed to remove duplicates. Predicted crinoid neuropeptide precursor sequences were pooled with the annotated echinoderm query sequences and used for a similarity-based clustering of sequences with CLANS (Frickey and Lupas, 2004; Supplementary Figure 1). This enabled prediction of homology of crinoid sequences with known neuropeptide precursors in other echinoderms. Clusters were annotated (based on echinoderm query sequences) at different *p*-value thresholds for different neuropeptide families, according to performance.

Analysis of the structure of predicted crinoid neuropeptide precursors was performed using Signal P 4.1 to predict signal peptides, whilst other features such as cleavage sites and putative mature peptides were manually annotated. Alignments of predicted crinoid mature neuropeptides with homologous neuropeptides in other echinoderms was performed using MAFFT (v7.470) (Katoh et al., 2002; Katoh and Standley, 2013).

### *De novo* identification of other predicted crinoid neuropeptide precursors

Sequences obtained using PrediSi (see above), but excluding sequences identified as homologs of known neuropeptide precursors in other echinoderms, were submitted to the online server NeuroPID (Ofer and Linial, 2014), which is a tool for neuropeptide precursor prediction based on machine learning. The outputs of NeuroPID were manually examined to check for evidence of neuropeptide precursor features, including the robustness of predicted signal peptides, presence of cleavage sites and other typical features such as glycine residues as sites for putative C-terminal amidation. Sequences that fulfilled most of the above characteristics were maintained as good candidates including 16 from *A. mediterranea*, 15 from *A. japonica*, and 15 from *F. serratissima*. Candidate neuropeptide precursor sequences from each species were then submitted as queries for BLASTP searches (E-value cut-off of 0.01) of the proteomes of the other two species, with removal of duplicates of hit sequences having cd-hits at 100%. This search identified a few sequences that had already been previously identified as homologs of known echinoderm neuropeptide precursors (see above), so these were removed. The remaining sequences were then filtered to remove sequences >1,000 amino acid residues in length, as it is highly unlikely that proteins of this length are neuropeptide precursors. The remaining 415 sequences were subject to cluster analysis using CLANS to identify clusters of candidate novel crinoid neuropeptide precursors (Supplementary Figure 2). Thirty-six clusters were identified and extracted and further manually examined to identify sequences with characteristics of neuropeptide precursors. Some sequences that had not been collected with previous data mining were identified as being homologous to known neuropeptide precursors, some were identified as additional predicted crinoid neuropeptide precursors (PCNP1-20) and others were discarded because they lacked known characteristics of neuropeptide precursor sequences (e.g., putative monobasic or dibasic cleavage sites).

### Analysis of the structure of genes encoding F-type and L-type SALMFamide precursors in *Anneissia japonica*

Transcripts encoding an *A. japonica* F-type SALMFamide precursor (Elphick et al., 2015) and a novel candidate *A. japonica* L-type SALMFamide precursor were identified in the *A. japonica* CDS genomic file downloaded from NCBI. Then the online tool Splign (Kapustin et al., 2008) was used to align the transcript sequences with the corresponding genes in the *A. japonica* genome assembly. The number, position and length of introns, the frame of the introns and the positions of neuropeptide-encoding sequences within exons were analyzed. Visual schematics of gene structures were constructed with IBS software^6^ (Liu et al., 2015) to enable comparison with the structure of genes encoding L-type and F-type SALMFamide precursors in the starfish *A. rubens*, the sea urchin *S. purpuratus* and the sea cucumber *A. japonicus*.

### Specimens of *Antedon mediterranea* used for experimental studies

Adult specimens of *A. mediterranea* were collected from Baia delle Grazie in the Gulf of La Spezia (Liguria, Italy) and then were brought to the University of Milan, where they were maintained in an artificial seawater system at ca. 15°C (salinity: ca. 37‰). Doliolaria larvae and pentacrinoids were reared from these captive animals, as described previously (Mercurio et al., 2019).

### Localization of neuropeptide precursor in *Antedon mediterranea* using mRNA *in situ* hybridization

#### Cloning and sequencing of neuropeptide precursor cDNAs and probe synthesis

Four cDNAs encoding neuropeptide precursors were selected for cloning: (i) F-type SALMFamide precursor, (ii) calcitonin-type precursor, (iii) orexin-type precursor, and (iv) vasopressin/oxytocin-type (crinotocin) precursor. A cDNA encoding the *A. mediterranea* F-type SALMFamide precursor was cloned into the pBluescriptSKII (+) vector (Agilent Technologies, Stockport, Cheshire, UK) and sequenced, as reported previously (Elphick et al., 2015). Using the same methodological approach, here cDNAs encoding the *A. mediterranea* calcitonin-type precursor (AmCTP) and the *A. mediterranea* orexin-type precursor (AmOXP) were cloned and sequenced. Informed by transcriptome sequence data, the following precursor-specific primers were used for polymerase chain reaction (PCR) cloning: 5′-CAGGGATA TACGGTCATCTTTT-3′/5′-GTGTTGCTTCTTGTTCTCTTCT-3′ (AmCTP) and 5′-GCGTTCCGTTTACCGACTAA-3′/5′- GGCGTGGSTGTTTTGGTATT-3′ (AmOXP). Efforts to clone and sequence a cDNA encoding the *A. mediterranea* vasopressin/oxytocin-type (crinotocin) precursor were unsuccessful and therefore a cDNA encoding this precursor was customized synthesized (GenScript) in the pBluescriptSKII (+) vector.

Digoxigenin (DIG)-labeled antisense and sense probes for the *A. mediterranea* F-type SALMFamide, calcitonin-type and vasopressin/oxytocin-type (crinotocin) precursor transcripts were generated using methods described previously for production of probes for neuropeptide precursor transcripts in the starfish *A. rubens* (Mayorova et al., 2016). Sense probes were used for negative control tests to assess the specificity of staining observed with antisense probes.

#### Whole-mount staining of *Antedon mediterranea* larvae and pentacrinoids

Whole-mount mRNA *in situ* hybridization was performed on doliolaria larvae and post-metamorphic pentacrinoids using a protocol described previously (Mercurio et al., 2019). Briefly, specimens were fixed in 4% paraformaldehyde, 0.5 M NaCl, 0.1 M 3-(N-morpholino) propanesulfonic acid (pH 7.5). Doliolariae were permeabilized with proteinase K (2 μg/ml) for 5 min at 37°C, while pentacrinoids were decalcified by incubating them in 5% EDTA in DEPC-treated water for 2 days. Specimens were incubated with probes at a concentration of 1μg/ml in hybridization buffer (50% formamide, 5× SSC, 100 μg/ml yeast RNA, 50 μg/ml heparin, 0.1% Tween-20) for 5 days at 65°C, while incubation with alkaline phosphatase-conjugated anti-DIG antibody was carried out overnight in blocking buffer (1:4 deactivated normal sheep serum in PBT) at room temperature. Finally, samples were incubated in APT buffer (5 M NaCl, 1 M MgCl_2_, 0.2 M Tris pH 9.5, 0.1% Tween-20) with NBT (nitroblue tetrazolium salt; Roche Diagnostics) and BCIP (5-bromo-4-chloro-3-indolylphosphate; Roche Diagnostics) until staining was observed, after which they were fixed in 4% PFA in DEPC-treated PBS. Larvae and pentacrinoids were mounted on slides in 80% glycerol and imaged with a QIClick CCD Colour Camera (Qimaging) linked to a DMRA2 light microscope (Leica), using Volocity^®^ v.6.3.1 image analysis software (PerkinElmer) installed on an iMac (27-inch, Late 2013 model with OS X Yosemite, Version 10.10).

#### Staining of sections of adult *Antedon mediterranea*

Adult specimens of *A. mediterranea* arms were fixed in 4% PFA in DEPC-treated PBS overnight or longer and then were decalcified in 10% EDTA in DEPC-treated autoclaved water solution (pH 7.4) for 7 days. Next specimens were dehydrated using increasing concentrations of ethanol up to 100%, briefly placed in xylene for clearing and then embedded in paraffin wax. Longitudinal and transverse sections of specimens (10 μm) were obtained using a microtome and then were mounted on glass slides (SuperFrost^®^ Plus, VWR). The method employed for visualization of mRNA transcripts in sections of adult specimens of *A. mediterranea* was a modified version of a protocol used previously in *A. rubens* (Lin et al., 2017b). First, slides were placed in xylene to remove wax and then slides were rehydrated through decreasing concentrations of ethanol, washed in PBS and then post-fixed in 4% PFA/PBS for 20 min. After washing in 0.1% Tween-20/PBS, tissue sections were permeabilized with a proteinase K solution (1 μg/ml in PBS) for 12 min at 37°C. This was followed by post-fixation in 4% PFA/PBS and then washing in 0.1% Tween-20/PBS. To remove positive charge from slides, an acetylation step was carried out with a solution of 0.1 M triethanolamine (TEA; pH 7–8) and 0.25% acetic anhydride for 10 min. Samples were washed in 0.1% Tween-20/PBS and then in 5X SSC. Sections were pre-hybridized for 1–2 h at room temperature in a pre-hybridization buffer (50% formamide, 50 μg/ml purified yeast RNA, 50 μg/ml heparin, 5X SSC, 0.1% Tween-20). After this, slides were incubated overnight at 65°C with probe at a concentration of 0.8–1 μg/ml in hybridization buffer solution. The next day slides were washed sequentially with the following solutions: 25% formaldehyde/5X SSC for 30 min at 65°C, 5X SSC for 30 min at 65°C, 0.2X SSC twice for 40 min at 65°C and 0.2X SSC at room temperature for 10 min. Then slides were equilibrated in buffer B1 (10 mM Tris-HCl pH 7.5 and 150 mM NaCl in autoclaved water) for 10 min. Slides were incubated for 2 h at room temperature with a solution of 5% normal goat serum (NGS) in B1 buffer to block non-specific binding sites for anti-digoxigenin antibodies. Then slides were incubated overnight at 4°C with the alkaline-phosphatase-conjugated anti-digoxigenin antibodies (Roche; 1:3,000 in B1 buffer with 2.5% NGS). The following day, slides were washed several times in B1 buffer and then B3 buffer (100 mM Tris-HCl pH 9.5, 100 mM NaCl and 50 mM MgCl_2_ in aqueous solution) for 10 min. Then slides were incubated in the dark with the staining solution (NBT 4.5 μl/ml and BCIP 3.5 μl/ml with 0.1% Tween-20 in B3 buffer). To stop the staining, slides were washed in autoclaved water. Slides were left to air-dry and then were washed for a few seconds in 100% ethanol and then for a few minutes in Histo-Clear and finally mounted with Histomount mounting solution.

### Immunohistochemistry

#### Whole-mount immunohistochemistry on pentacrinoids of *Antedon mediterranea*

Specimens of pentacrinoids were processed for whole-mount immunofluorescence labeling as described previously (Mercurio et al., 2019). After decalcification for 2 days in 5% EDTA in water specimens were permeabilized in 0.25% Triton-X for 30 min. Then they were incubated overnight at 4°C with a rabbit antiserum to the *A. rubens* calcitonin-type neuropeptide ArCT (Cai et al., 2018) diluted 1:3,000 in 1:1 NGS:PBS. This was followed by washes and incubation with Cy2-labeled goat anti-rabbit immunoglobulins (1:400; Jackson ImmunoResearch Europe Ltd., UK). Samples were then imaged using a Leica SP5 confocal microscope in combination with the Leica Application Suite Advanced Fluorescence (LAS AF; version 2.6.0.7266) program. The specificity of immunofluorescence observed in experiments with the ArCT antiserum was assessed by performing experiments in parallel with ArCT antiserum pre-absorbed with the ArCT antigen peptide (Cai et al., 2018). For these experiments, the ArCT antiserum was first diluted to 1:300 and then incubated with 20 μM ArCT antigen peptide for 2 h at room temperature. Then the pre-absorbed antiserum was diluted 10-fold to 1:3,000 and tested as described above.

#### Immunohistochemistry on sections of adult *Antedon mediterranea*

For immunohistochemistry on sections of arms and calyxes from adult *A. mediterranea*, an alcoholic Bouin’s fixative (5.3% picric acid, saturated alcoholic; 48% absolute ethanol; 13.3% water; 27% formalin, concentrated; 6.7% glacial acetic acid) (Holland, 1967) was used for fixation and decalcification of ossicles. Use of alcoholic Bouin’s fixative enabled decalcification without evolution of CO_2_ bubbles that would otherwise cause tissue damage (Holland, 1967; N.D. Holland, pers. comm.). After fixation, specimens were washed repeatedly in 50% ethanol, taken to 100% ethanol, placed briefly in xylene for clearing and then embedded in paraffin wax. Longitudinal and transverse sections of 10 μm were obtained using a microtome and mounted on chrome alum/gelatin-coated glass slides.

The immunohistochemistry protocol used was based on methods employed previously for analysis of neuropeptide expression in sections of the starfish *A. rubens* (Lin et al., 2017a). Slides were first washed in xylene to dissolve the paraffin wax and then placed in 100% ethanol. A 30 min incubation in 1% hydrogen peroxide in methanol was carried out to block endogenous peroxidase activity. Then rehydration was carried out by transferring slides through a series of decreasing concentrations of ethanol. The slides were then washed sequentially with distilled water, 1X PBS and then with a solution of 0.1% Tween-20 in 1X PBS (PBST). Then to block non-specific binding sites for secondary antibodies, a solution of 5% NGS in PBST was applied to each slide for 2 h in humidified chambers. After the blocking step, slides were incubated overnight at 4°C with a rabbit antiserum to the *A. rubens* calcitonin-type neuropeptide ArCT (Cai et al., 2018) diluted 1:3,000 in 1:1 NGS:PBS. Then slides were washed several times with PBST before they were incubated for 3 h at room temperature with peroxidase-conjugated AffiniPure goat anti-rabbit immunoglobulins (1:1,000 with 2% NGS in PBST; Jackson ImmunoResearch Laboratories, West Grove, PA, USA). After several washes in PBST, the DAB staining solution [0.05% diaminobenzidine (VWR Chemicals, Lutterworth, UK), 0.05% nickel chloride, 0.015% hydrogen peroxide diluted in PBS] was added to each slide. As soon as staining appeared, slides were washed in distilled water. Subsequently, slides were dehydrated with ethanol solutions of increasing concentrations up to 100%, washed in xylene and then mounted with coverslips using DPX as mounting medium. Images of immunostained sections were captured with a QIClick CCD Colour Camera (Qimaging) linked to a DMRA2 light microscope (Leica), using Volocity^®^ v.6.3.1 image analysis software (PerkinElmer) installed on an iMac (27-inch, Late 2013 model with OS X Yosemite, Version 10.10).

### Investigation of *in vitro* pharmacological effects of the vasopressin/oxytocin-type neuropeptide crinotocin on arm preparations from *Antedon mediterranea*

#### Arm preparations from *Antedon mediterranea*

**FIGURE 1:**
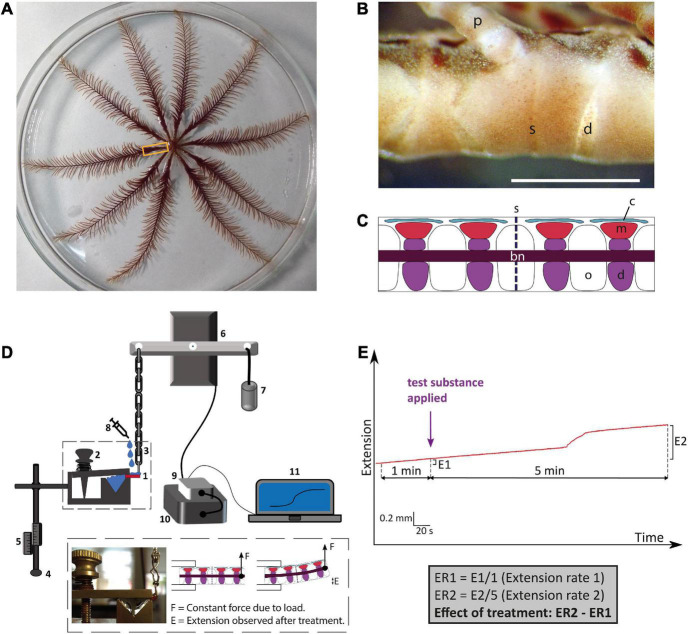
Anatomy of the whole-body and arms of adult *Antedon mediterranea* and experimental set-up used to investigate effects of neuropeptides on the mechanical behavior of arm preparations. **(A)** Photograph of a whole adult specimen of *A. mediterranea* in an 18 cm diameter petri dish. The rectangle shows the region of an arm nearest to the calyx, which corresponds to the region from which experimental preparations were dissected. **(B)** Photograph of lateral view of an arm, oral side up and proximal end to the right. The scale bar is 1 mm. d, diarthrosis; s, syzygy; p, pinnule. **(C)** A schematic drawing of a sagittal section of an arm equivalent to the portion shown in panel **(B)**. Major anatomical structures are labeled as follows: bn, brachial nerve; c, coelom; d, diarthrosis; m, muscle; o, ossicle; s, syzygy. **(D)** Schematic drawing of the experimental set-up for testing the effects of neuropeptides on the mechanical properties of arm preparations. The arm preparation (1) is orientated with its oral side up, as is the natural position of the arm, and is clasped at its proximal end by a spring-loaded clamp (2) and connected at its distal end via a heart clip to a chain (3). The exact positioning of the arm preparation in relation to the apparatus can be regulated by a system of rods that are adjusted using clamp manipulators (4) and scales (5). The chain is connected to an isotonic lever transducer (6) and at the opposite end of the lever is a weight of 10 g (7), which creates a tension in the arm preparation similar to that in an arm outstretched for food collection in the animal’s natural environment (La Touche, 1978; Byrne and Fontaine, 1981). Test substances (e.g., neuropeptides) are administered to the preparation using a syringe (8). Changes in the mechanical behavior of the arm preparation are detected by the transducer and relayed via a bridge amplifier (9) to acquisition hardware (10) and then finally to a computer (11), where recordings are made and stored using LabChart software. The inset shows a close-up of an arm preparation positioned in the apparatus and a schematic representation of arm extension after application of a test substance. **(E)** Schematic representation of the method used to quantify effects of test substances on arm extension. The basal rate of extension is measured during the 1 min prior to application of the test substance (E1). After application of the test substance, recording is continued for a period of 5 min and the extension observed in this testing period is E2. The average extension rates (ER: mm min^–1^) in the 1 min before and 5 min after application are then calculated, and the effect of the test substance is quantified as ER2 – ER1.

Experiments investigating the effects of neurotransmitters on muscles and collagenous ligaments that link skeletal elements in the arms of *A. mediterranea* have been reported previously (Wilkie et al., 2010) and here the same methodology was used to investigate *in vitro* pharmacological effects of the vasopressin/oxytocin-type neuropeptide crinotocin. The main skeletal elements of the arms are the brachial ossicles, which form a series connected by collagenous ligaments at two types of joints: syzygies are rigid joints specialized for autotomy (defensive detachment) and diarthroses are mobile joints which are also subtended by muscles whose contraction causes upward flexion. Arm preparations used here for the experiments were prepared as described previously (Wilkie et al., 2010) and were dissected under a microscope from animals anesthetized by immersion in artificial sea water (ASW) containing 0.1% propylene phenoxetol (1-phenoxy-2-propanol) (Figure 1A). Each sample consisted of a short length of arm containing one syzygy in the middle and normally two diarthroses on either side of the syzygy (Figures 1B,C). Arm preparations were dissected by starting from the portion of the arm nearest to the calyx, until four preparations were collected from each arm, with up to all ten arms being used from each animal. Arm preparations were kept in 24-well plates with ASW in an incubator at ca. 15°C until they were used for experiments. A schedule of alternate testing for the arm preparations was followed to minimize effects of time spent in incubator and randomize which portion in the proximal-distal sequence was used for test vs. control experiments.

#### Measurement of mechanical responses of arm preparations from *Antedon mediterranea*

To enable measurement of mechanical responses of arm preparations to applied test agents, an experimental set-up described previously (Wilkie et al., 2010) was used. Arm preparations were mounted on a mechanical testing apparatus horizontally and with the oral side uppermost. The distal end of the preparation was fixed with a spring-loaded clamp, while the proximal end was held with a heart-clip connected to a silver chain attached to one side of the lever of an isotonic transducer. At the other end of the lever was a weight of 10 g. With this set-up, the posture of the arms imitates the natural state in which the animal extends the arms to capture food particles (La Touche, 1978; Byrne and Fontaine, 1981). Output from the transducer was passed through a bridge amplifier and then to the acquisition hardware (PowerLab; AD Instruments) and a computer where it was recorded using LabChart (v.7) software (Figure 1D). Once an arm preparation was mounted, ASW was immediately pipetted onto it to ensure that it remained moist. After each sample was tested, the equipment was washed and dried before testing the next sample.

#### Investigating effects of crinotocin on arm preparations from *Antedon mediterranea*

Informed by identification of a transcript encoding a VP/OT-type neuropeptide precursor in *A. mediterranea*, the VP/OT-type neuropeptide crinotocin was synthesized (Peptide Protein Research Ltd, Fareham, UK). In accordance with the known mature structures of VP/OT-type neuropeptides in other taxa (Odekunle and Elphick, 2020), crinotocin was synthesized with a disulphide bridge between the two underlined cysteine residues and with C-terminal amidation (CFWRTCPVG-NH_2_). Solutions of crinotocin in artificial seawater (ASW) were prepared at a concentration of 10^–5^ M to enable preliminary testing at a physiologically relevant concentration. After mounting arm preparations in the testing apparatus as described above, the sample was kept moist and allowed to stabilize for ca. 3 min before application of test substances. Recording was carried out for at least 5 min after application of test substances to allow time for development of any effect and ASW was used as a negative control. This protocol was used for a total of 81 arm preparations from four animals. Effects were registered as changes in the rate of extension (ER) (extension being, in this context, displacement resulting from upward bending of preparations) and were quantified as ER2 − ER1: the difference between the mean extension rate in the 1 min before application of the test substance (ER1) and the mean extension rate in the 5 min after application of the test substance (ER2) (Figure 1E).

To investigate if any observed effects of crinotocin were specific to this peptide and not a non-specific effect of peptides in general, we also tested three neuropeptides from the starfish *A. rubens*: the SALMFamides S1 and S2 (Elphick et al., 1991a) and the luqin-type neuropeptide ArLQ (Yañez-Guerra et al., 2018). Homologs of these neuropeptides have been identified in *A. mediterranea* but with differences in their sequences and therefore crinotocin was the only neuropeptide tested that occurs naturally in *A. mediterranea.* The three *A. rubens* neuropeptides were dissolved in ASW, with sonication when necessary, at a concentration of 10^–5^ M and were tested on arm preparations using the same method as described above for crinotocin.

Having established that crinotocin has a significant and specific effect on the mechanical behavior of *A. mediterranea* arm preparations when tested at a concentration of 10^–5^ M, the dose-dependence of the effect of crinotocin was investigated by testing crinotocin at concentrations of 10^–4^, 10^–5^, 10^–6^, 10^–7^, and 10^–8^ M using the same method described above for tests with 10^–5^ M crinotocin. For these experiments a total of 143 arm preparations from 4 animals were used and GraphPad Prism software was used to fit data to a curve with non-linear regression.

To investigate the mechanism of action of crinotocin, arm preparations were anesthetized prior to testing to assess if effects were nervously mediated. Samples were incubated in the anesthetic procaine hydrochloride (10^–3^ M in ASW) for ca. 1 h and then the same experimental protocol as described above was used. For the test experiments, a solution of crinotocin (10^–5^ M) plus anesthetic was administered. As controls, some arm preparations were incubated for the same length of time in ASW and then tested either with only crinotocin (10^–5^ M; positive control) or ASW (negative control). In total, 106 arm preparations from three animals were used for this experiment.

For statistical analysis of data obtained from *in vitro* pharmacological experiments, Excel was used to conduct Student’s two-tailed *t*-test or, for more than two comparisons, one-way ANOVA followed by the Bonferroni *post-hoc* test.

## Results

### Discovery of crinoid representatives of known neuropeptide families

**FIGURE 2:**
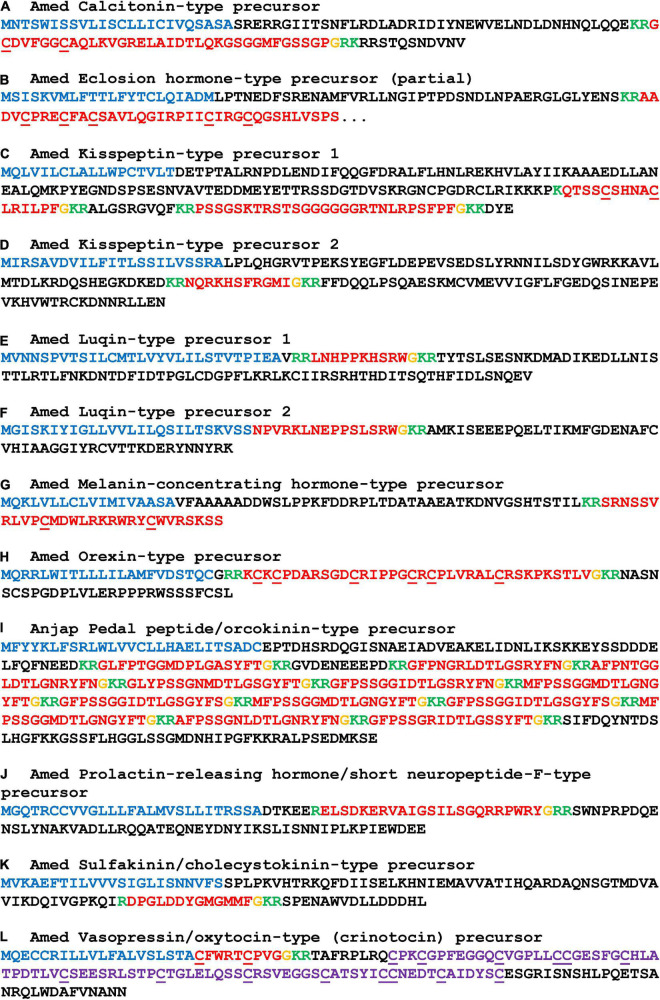
Sequences of crinoid proteins that are precursors of monomeric neuropeptides. Neuropeptide precursor sequences are shown in alphabetical order and are from *Antedon mediterranea* (Amed), with the exception of pedal peptide/orcokinin-type precursor which was not identified in this species but which was identified in *Anneissia japonica* (Anjap). The predicted signal peptide is shown in blue, predicted monobasic/dibasic cleavage sites are shown in green and predicted neuropeptides are shown in red, but with C-terminal glycine residues that are potential substrates for post-translational amidation shown in orange. The underlined cysteine residues have been shown to form intramolecular disulphide bridges in homologous monomeric neuropeptides in other taxa. The neurophysin domain of the vasopressin/oxytocin-type (crinotocin) precursor is shown in purple. The DNA sequences of transcripts encoding these precursors are shown in Supplementary File 2.

**FIGURE 3:**
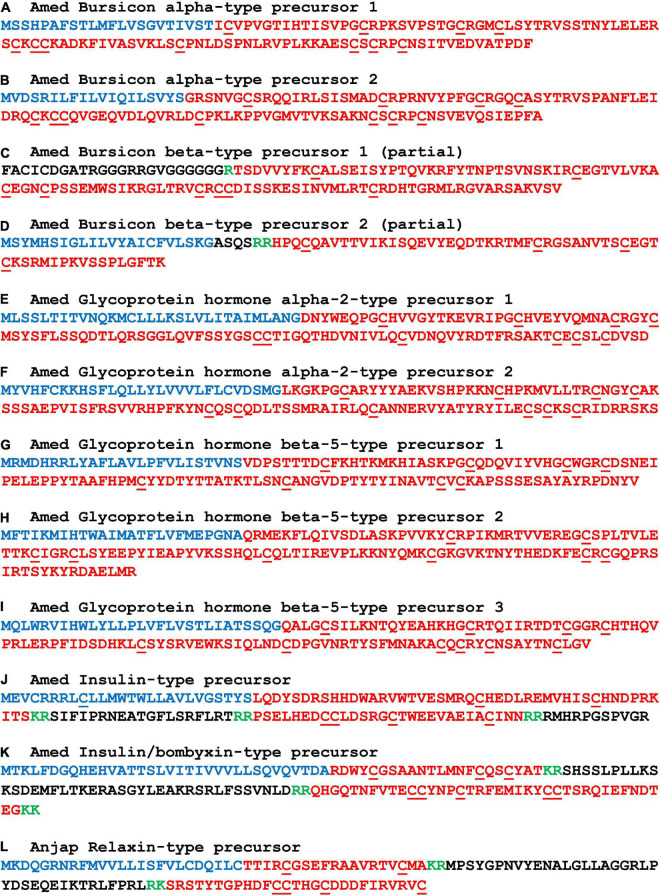
Sequences of crinoid proteins that are precursors of heterodimeric neuropeptides. Neuropeptide precursor sequences are shown in alphabetical order and are from *Antedon mediterranea* (Amed), with the exception of relaxin-type precursor which was not identified in this species but which was identified in *Anneissia japonica* (Anjap). The predicted signal peptide is shown in blue, predicted monobasic/dibasic cleavage sites are shown in green and predicted neuropeptides are shown in red. The underlined cysteine residues have been shown to form intramolecular or intermolecular disulphide bridges in homologous heterodimeric neuropeptides in other taxa. The DNA sequences of transcripts encoding these precursors are shown in Supplementary File 2.

**FIGURE 4:**
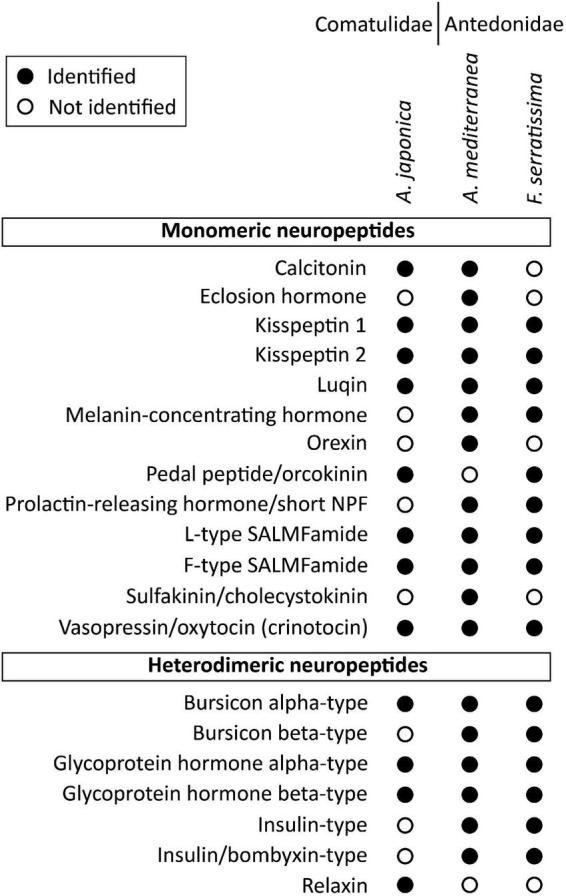
Summary showing in which species the monomeric and heterodimeric neuropeptide precursors have been identified in this study. Filled circles represent cases where neuropeptide precursors were identified in a given species, whereas empty circles represent cases in which the precursors were not identified. Where neuropeptide precursors were not identified, this may reflect incomplete transcriptome/genome sequence data or loss in one or more species. The DNA sequences of transcripts encoding these precursors are shown in Supplementary File 2.

**FIGURE 5:**
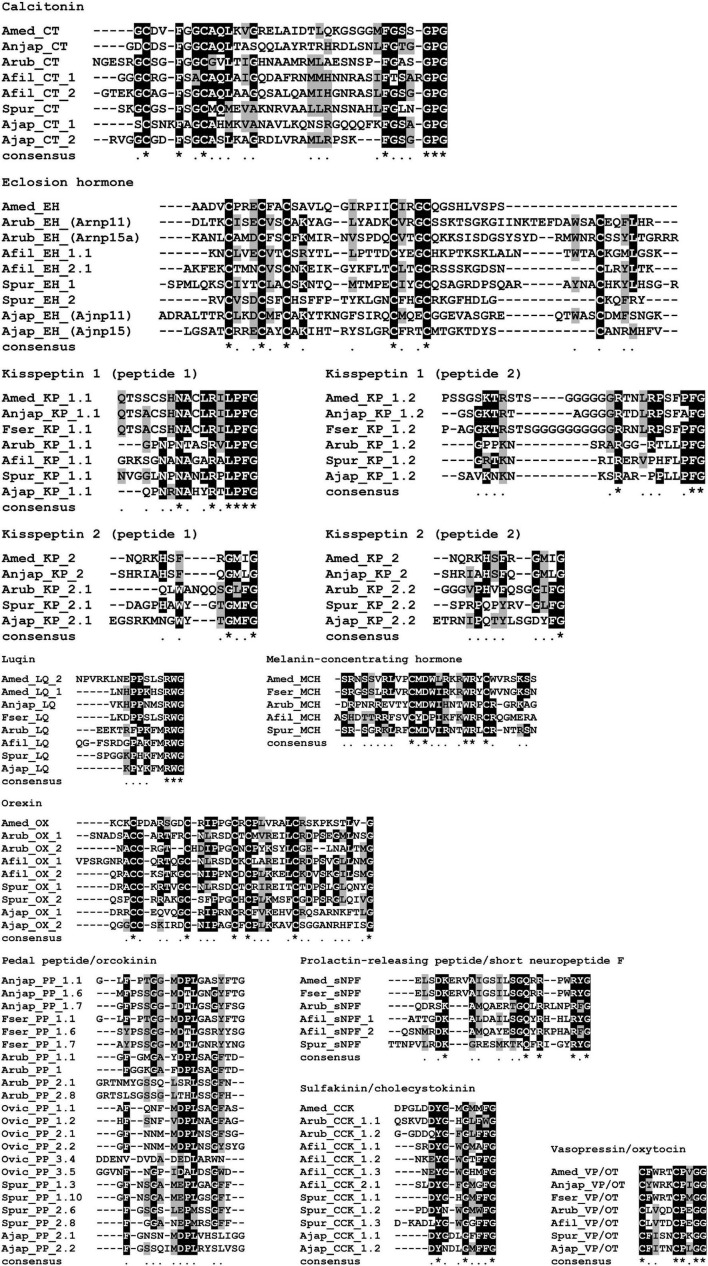
Alignments of predicted crinoid monomeric neuropeptides with homologous neuropeptides in eleutherozoan echinoderms. Neuropeptide sequences from all three crinoid species are shown, where known, and aligned with the sequences of homologous neuropeptides, where known, from two asterozoan species (starfish and brittle star) and two echinozoan species (sea urchin and sea cucumber). Species name abbreviations: Amed, *Antedon mediterranea*; Anjap, *Anneissia japonica*; Fser, *Florometra serratissima*; Arub, *Asterias rubens* (starfish); Afil, *Amphiura filiformis* (brittle star); Spur, *Strongylocentrotus purpuratus* (sea urchin) and Ajap, *Apostichopus japonicus* (sea cucumber). Alignments were performed using MAFFT (v7.470) with default parameters and conserved amino acids were highlighted using a threshold fraction of conserved sequences of 0.60 with Boxshade (https://manpages.ubuntu.com/manpages/bionic/man1/boxshade.1.html).

**FIGURE 6:**
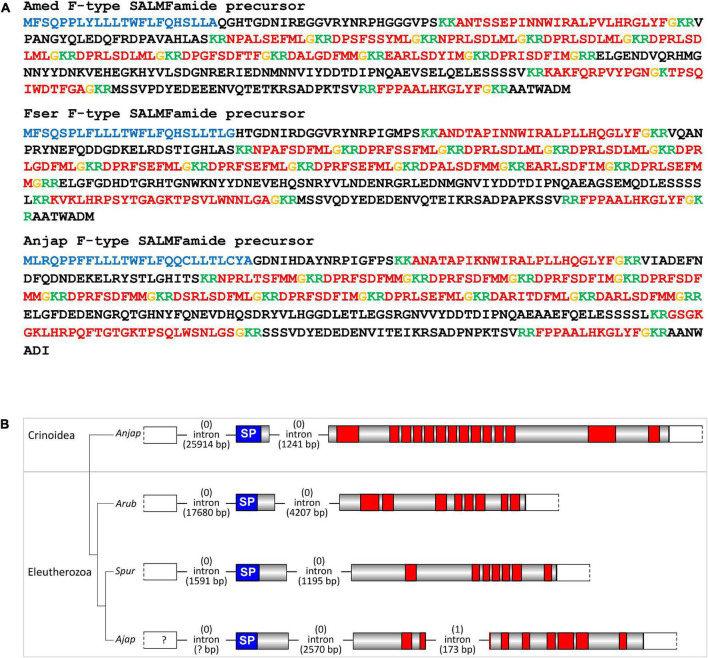
Crinoid F-type SALMFamide precursors and comparison of gene structure with eleutherozoan F-type SALMFamide precursor genes. **(A)** Sequences of F-type SALMFamide precursors in three crinoid species, *Antedon mediterranea* (Amed), *Florometra serratissima* (Fser), and *Anneissia japonica* (Anjap). The predicted signal peptide is shown in blue, predicted monobasic/dibasic cleavage sites are shown in green and predicted neuropeptides are shown in red, but with C-terminal glycine residues that are potential substrates for post-translational amidation shown in orange. **(B)** Structure of the F-type SALMFamide precursor gene in the crinoid *Anneissia japonica* (Anjap) compared with the structures of F-type SALMFamide precursor genes in eleutherozoans, including the starfish *Asterias rubens* (Arub), the sea urchin *Strongylocentrotus purpuratus* (Spur), and the sea cucumber *Apostichopus japonicus* (Ajap). In all four species there are two phase 0 introns, with one preceding the start codon encoding the first methionine of the signal peptide and another located between an exon encoding the signal peptide and an exon or exons encoding the SALMFamide neuropeptides, which provides additional evidence that these genes are orthologs. The *A. japonicus* gene also has a second intron in phase 1 that interrupts the coding sequence for the second predicted SALMFamide neuropeptide.

**FIGURE 7:**
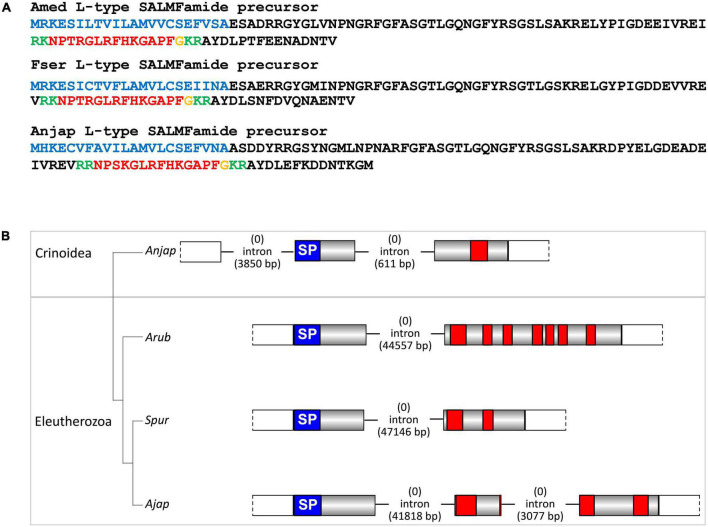
Crinoid L-type SALMFamide precursors and comparison of gene structure with eleutherozoan L-type SALMFamide precursor genes. **(A)** Sequences of L-type SALMFamide precursors in three crinoid species, *Antedon mediterranea* (Amed), *Florometra serratissima* (Fser), and *Anneissia japonica* (Anjap). The predicted signal peptide is shown in blue, predicted dibasic cleavage sites are shown in green and predicted neuropeptides are shown in red, but with C-terminal glycine residues that are potential substrates for post-translational amidation shown in orange. **(B)** Structure of the L-type SALMFamide precursor gene in the crinoid *Anneissia japonica* (Anjap) compared with the structures of L-type SALMFamide precursor genes in eleutherozoans, including the starfish *Asterias rubens* (Arub), the sea urchin *Strongylocentrotus purpuratus* (Spur), and the sea cucumber *Apostichopus japonicus* (Ajap). In all four species there is phase 0 intron located between an exon encoding the signal peptide and an exon or exons encoding the SALMFamide neuropeptides, which provides additional evidence that these genes are orthologs. The *A. japonica* gene has a second phase 0 intron preceding the start codon encoding the first methionine of the signal peptide and the *A. japonicus* gene also has a second phase 0 intron that interrupts the coding sequence for the second predicted SALMFamide neuropeptide.

Consistent with previous studies that analyzed transcriptomic/genomic sequence data from eleutherozoan echinoderms (Asteroidea, Ophiuroidea, Echinoidea, and Holothuroidea) (Rowe and Elphick, 2012; Rowe et al., 2014; Semmens et al., 2016; Smith et al., 2017; Zandawala et al., 2017; Suwansa-Ard et al., 2018; Chen et al., 2019) analysis of crinoid sequence data enabled identification of transcripts/genes encoding precursors of neuropeptides that belong to metazoan, bilaterian, deuterostome-specific or echinoderm-specific neuropeptide families. We present the sequences of these precursor proteins in several figures. Firstly, in Figures 2, 4, 5 we present crinoid representatives of neuropeptide precursor families that give rise to one or more monomeric neuropeptides, which include calcitonin, eclosion hormone, kisspeptin, luqin, melanin-concentrating hormone (MCH), orexin, pedal peptide/orcokinin, prolactin-releasing peptide/short neuropeptide-F, sulfakinin/cholecystokinin and vasopressin/oxytocin (crinotocin). Secondly, in Figures 3, 4 we present crinoid representatives of neuropeptide precursor families that give rise to heterodimeric neuropeptides, which include bursicon, glycoprotein hormone, insulin, insulin/bombyxin-like and relaxin. Thirdly, in Figures 6, 7 we present crinoid precursors of SALMFamide-type neuropeptides, which have thus far only been identified in echinoderms.

Because we have used *A. mediterranea* as an experimental model for investigation of neuropeptide expression and action in crinoids (see below), we preferentially present sequence data from this species (Figures 2–7). However, some of the neuropeptide precursors identified in the crinoid species for which a genome sequence is available, *A. japonica*, were not found in our *A. mediterranea* transcriptome data. Therefore, for these neuropeptide precursors we present in Figures 2, 3 the sequences of the *A. japonica* proteins. Furthermore, in Figure 4 we summarize the neuropeptide precursor sequence data that we have obtained from the three species analyzed in this study, *A. mediterranea, F. serratissima*, and *A. japonica*, and in Supplementary File 1 we present the sequences of neuropeptide precursors from all three crinoid species and in Supplementary File 2 we provide the corresponding nucleotide sequences.

#### Precursors of monomeric neuropeptides

The sequences of monomeric crinoid neuropeptides were compared with orthologous peptides that have been identified in species belonging to the four other extant echinoderm classes (eleutherozoans) (Figure 5). The rationale for doing this was 2-fold. Firstly, from a practical perspective we were interested in determining the extent of sequence conservation between the crinoid peptide and orthologs in other echinoderms to provide a basis for evaluation of potential cross-reactivity of the crinoid peptides with antibodies to neuropeptides in the starfish *A. rubens* that have been generated previously. Secondly, from an evolutionary perspective we were interested in investigating if the phylogenetic position of crinoids as a sister group to eleutherozoan echinoderms is reflected in neuropeptide sequence similarity.

##### Calcitonin-type precursor

The *A. mediterranea* calcitonin-type precursor (AmCTP) is a 116-residue protein that comprises a predicted 35-residue calcitonin-type peptide (AmCT) and this sequence was determined by analysis of transcriptome sequence data and cDNA cloning and sequencing (Figure 2A). Informed by the occurrence of post-translational modifications of calcitonin-type peptides in other species, including the starfish *A. rubens* (Semmens et al., 2016; Cai et al., 2018), AmCT is predicted to be C-terminally amidated and to have a disulphide bridge between the two cysteine residues in the N-terminal region of the peptide. Alignment of the sequence of AmCT with calcitonin-type peptide sequences in other echinoderms reveals regions of sequence identity in both the N-terminal and C-terminal regions of the peptides but not in the intervening region (Figure 5). Furthermore, it is noteworthy that the C-terminal region of AmCT (FGSSGP-NH_2_) shares sequence similarity with a peptide antigen (FGASGP-NH_2_) that has been used for production of antibodies to the starfish calcitonin-type peptide ArCT (Cai et al., 2018), indicating that these antibodies may cross-react with AmCT (see below).

##### Eclosion hormone-type precursors

A partial sequence of an eclosion hormone-type precursor was identified in *A. mediterranea* (AmEHP) (Figure 2B). Two eclosion hormone-type precursors have been identified in eleutherozoan species and these comprise peptides that have six cysteine residues, in common with eclosion hormone-type peptides in other phyla. Five of the six cysteine residues are present in the partial AmEHP sequence. Further studies will be required to determine full-length sequences of eclosion hormone-type precursors in crinoids.

##### Kisspeptin-type precursors

The occurrence of kisspeptin-type precursors in echinoderms was first reported with the identification of a protein in *A. rubens* (ArKPP), which comprises two kisspeptin-like peptides (Semmens et al., 2016), and orthologs of this protein have been identified in other echinoderms (Zandawala et al., 2017; Chen et al., 2019). Recently, a second kisspeptin-type precursor, also comprising two kisspeptin-like peptides, was identified in *A. rubens* and other echinoderms (Escudero Castelán et al., 2022). Therefore, these two precursor types are now referred to as KPP1 and KPP2, respectively, and here we report their identification in crinoids.

The *A. mediterranea* kisspeptin-type precursor 1 (AmKPP1) is a 189-residue protein that comprises two putative kisspeptin-like peptides (AmKP1.1, AmKP1.2), both of which are predicted to be C-terminally amidated (Figure 2C). Accordingly, kisspeptin-type precursors in eleutherozoans also comprise two putative C-terminally amidated kisspeptin-type peptides (e.g., ArKP1.1 and ArKP1.2 in *A. rubens*; Semmens et al., 2016; Escudero Castelán et al., 2022). Alignment of crinoid KP1.1-type peptides with eleutherozoan KP1.1-type peptides revealed a conserved C-terminal Arg-X-Leu-Pro-Phe-NH_2_ (RXLPFamide, where X is variable) motif and a conserved Asn (N) residue in the core of the peptide sequences (Figure 5). Furthermore, an interesting and unusual feature of AmKP1.1 and the KP1.1-type peptides in *A. japonica* and *F. serratissima* is the presence of two cysteine residues. Alignment of crinoid KP1.2-type peptides with eleutherozoan KP1.2-type peptides revealed a conserved C-terminal Pro-Phe-NH_2_ motif and a conserved Arg (R) residue in the core of the peptide sequences (Figure 5).

The *A. mediterranea* kisspeptin-type precursor 2 (AmKPP2) is a 153-residue protein that comprises a single putative kisspeptin-like peptide (AmKP2), which is predicted to be C-terminally amidated (Figure 2D). This contrasts with kisspeptin-type precursors in eleutherozoans that comprise two putative C-terminally amidated kisspeptin-type peptides (e.g., ArKP2.1 and ArKP2.2 in *A. rubens*; Semmens et al., 2016; Escudero Castelán et al., 2022). Sequence alignment revealed that crinoid KP2-type peptides have a C-terminal Gly-Met-X-NH_2_ motif (where X is variable) and a histidine (H) residue in the core of the peptides that is also a feature of one or more eleutherozoan KP2.1-type peptides. Similarly, crinoid KP2-type peptides have a C-terminal Gly-X-X-NH_2_ motif in common with eleutherozoan KP2.2-type peptides and a His-X-Phe (H-X-F) motif in the core of the crinoid KP2-type peptides that is shared with some eleutherozoan KP2.2-type peptides (Figure 5).

##### Luqin-type precursors

Two luqin-type neuropeptide precursors (AmLQP1 and ArLQP2) were identified in *A. mediterranea*. AmLQP1 is a 123-residue protein that comprises a predicted 11-residue luqin-type peptide with a C-terminal glycine residue that is a potential substrate for amidation. Therefore, the neuropeptide derived from this precursor (AmLQ1) is predicted to be a C-terminally amidated 10-residue peptide (Figure 2E). AmLQP2 is a 92-residue protein that comprises a predicted 16-residue luqin-type peptide with a C-terminal glycine residue that is a potential substrate for amidation. Therefore, the neuropeptide derived from this precursor (AmLQ2) is predicted to be a C-terminally amidated 15-residue peptide (Figure 2F). In contrast to the occurrence of two luqin-type precursors in *A. mediterranea*, in *A. japonica*, and *F. serratissima* single luqin-type precursors were identified. In this respect, *A. mediterranea* is atypical because in other echinoderms only one luqin-type precursor has been identified in each species analyzed. A characteristic feature of luqin-type neuropeptides in ambulacrarians is a C-terminal Arg-Trp-NH_2_ (RWamide) motif, contrasting with the Arg-Phe-NH_2_ (RFamide) or Arg-Tyr-NH_2_ (RYamide) motifs that are characteristic of luqin/RYamide-type neuropeptides in protostomes (Yañez-Guerra and Elphick, 2020). Accordingly, the luqin-type neuropeptides in crinoids have a RWamide motif. Furthermore, it is noteworthy that the crinoid luqin-type peptides have a C-terminal SRWamide motif, whereas luqin-type peptides in eleutherozoan echinoderms have a C-terminal MRWamide motif. Another characteristic feature of the crinoid luqin-type peptides is a pair of adjacent proline residues, whereas in eleutherozoan echinoderms the luqin-type peptides have a single proline residue in an equivalent position to one of the two prolines in the crinoid peptides (Figure 5).

##### Melanin-concentrating hormone-type precursor

The *A. mediterranea* melanin-concentrating hormone-type precursor (AmMCHP) is a 91-residue protein that comprises a predicted 29-residue MCH-type peptide (AmMCH) (Figure 2G). Informed by the occurrence of post-translational modifications of MCH-type peptides in vertebrates, mature AmMCH and MCH-type peptides in other echinoderms are predicted to have a disulphide bridge between the two conserved cysteine residues (Semmens et al., 2016). Other residues that are conserved between the echinoderm MCH-type peptides aligned in Figure 5 are aspartate (D), tryptophan (W), and arginine (R) residues at positions 13, 19, and 20 in AmMCH, which are all located between the conserved cysteine residues at positions 11 and 22 in AmMCH. Structural features that distinguish the crinoid MCH-type peptides from eleutherozoan MCH-type peptides are the residue at position 6 in AmMCH, which is valine/leucine (V/L) in crinoids and arginine (R) in eleutherozoans, and the residue at position 23 in AmMCH, which is tryptophan (W) in crinoids and arginine (R) in eleutherozoans.

##### Orexin-type precursor

A partial sequence of the *A. mediterranea* orexin-type precursor (AmOXP) was obtained from analysis of transcriptome sequence data. However, cDNA cloning and sequencing enabled determination of the entire coding sequence, which encodes a 92-residue protein that comprises a 37-residue orexin-like peptide with a C-terminal glycine residue that is a potential substrate for amidation (Figure 2H). Therefore, the neuropeptide derived from this precursor (AmOX) is predicted to be a C-terminally amidated 36-residue peptide. In common with orexin-type peptides identified in other echinoderms, AmOX contains six cysteine residues, which may form up to three disulphide bridges. However, it is noteworthy that in eleutherozoan orexin-type peptides the first two cysteines are adjacent residues, whereas in AmOX the first two cysteines are separated by a lysine (K) residue. Furthermore, in eleutherozoans there are two orexin-type neuropeptide precursors (e.g., ArOXP1 and ArOXP2 in *A. rubens*; Semmens et al., 2016), whereas only a single orexin-type precursor was identified in *A. mediterranea.* Interestingly, comparison of the sequence of AmOX with OX1-type and OX2-type peptides in eleutherozoans reveals sequence identity that is shared with both OX1-type and OX2-type peptides and sequence identity that is shared only with OX1-type or OX2-type peptides. Therefore, duplication of a gene encoding an AmOX-like peptide in a common ancestor of the Eluetherozoa may have given rise to the occurrence of two genes encoding OX-type peptides in eleutherozoans.

##### Pedal peptide/orcokinin-type precursor

A pedal peptide/orcokinin (PP/OK)-type precursor was identified in *A. japonica* and in *F. serratissima* but not in *A. mediterranea* and therefore in Figure 2 the sequence of the *A. japonica* pedal peptide/orcokinin(PP/OK)-type precursor (AnjapPPLNP) is shown as a representative example. AnjapPPLNP is a 379-residue protein that comprises 12 copies of PP/OK-type neuropeptides (Figure 2I). Alignment of representatives of these crinoid PP/OK-type peptides with PP/OK-type peptides that have been identified in eleutherozoan echinoderms revealed several conserved features, including a phenylalanine residue located near the N-terminus, an Asp-X-Leu motif (where X is Pro or Thr) in the core of the peptide sequences and an aromatic residue (Phe or Tyr) located near to the C-terminus. Accordingly, analysis of the structure-activity relationships of a PP/OK-type neuropeptide in the starfish *P. pectinifera* has revealed the importance of these conserved residues for the bioactivity (Kim et al., 2018). However, two PP/OK-type precursor proteins have been identified in the starfish *A. rubens*, which are referred to as ArPPLNP1 and ArPPLNP2 (Semmens et al., 2016; Lin et al., 2017a, 2018). It is noteworthy, therefore, that the alignment in Figure 5 shows that the crinoid PP/OK-type peptides share more sequence similarity with peptides derived from ArPPLNP1 than with peptides derived from ArPPLNP2. Thus, ArPPLNP1-like precursors have now been identified in all five extant echinoderm classes, whereas ArPPLNP2-like precursors have thus far only been identified in starfish and brittle star species (Zandawala et al., 2017) (i.e., in Asterozoa).

##### Prolactin-releasing peptide/short neuropeptide-F-type precursor

The *A. mediterranea* prolactin-releasing peptide/short neuropeptide-F-type precursor (AmPrRPP) is a 112-residue protein that comprises a predicted 23-residue PrRP-type peptide (AmPrRP) (Figure 2J). Informed by the occurrence of post-translational modifications of PrRP peptides in other species, including the starfish *A. rubens* (Yañez-Guerra et al., 2020), AmPrRP is predicted to be C-terminally amidated. Alignment of AmPrRP with PrRP-type peptides in other echinoderms reveals several conserved residues, including the lysine, glutamine, arginine and arginine residues at positions 5, 17, 19, and 22 in AmPrRP (Figure 5). However, a feature of the crinoid PrRP-type peptides that distinguishes them from PrRP-type peptides in eleutherozoan echinoderms is the presence of a Glu-Arg-Val (ERV) motif following the lysine residue at position 5. Informed by the phylogenetic relationships of extant echinoderms, two evolutionary scenarios could explain this difference. The presence of the ERV motif could be an ancestral characteristic of extant echinoderms that has been lost in the eleutherozoan lineage or it could be a derived characteristic that has been acquired in the crinoid lineage.

##### Sulfakinin/cholecystokinin-type precursor

The *A. mediterranea* sulfakinin/cholecystokinin-type precursor (AmSK/CCKP) is a 112-residue protein that comprises a predicted 13-residue SK/CCK-type peptide (AmSK/CCK) (Figure 2K). Informed by the occurrence of post-translational modifications of SK/CCK peptides in other species, including the starfish *A. rubens* (Tinoco et al., 2021), AmSK/CCK is predicted to be C-terminally amidated and with sulfation of a single tyrosine residue. By comparison with SK/CCK-type precursors that have been identified in other echinoderms, it is noteworthy that AmSK/CCKP comprises only a single SK/CCK-type peptide, whereas SK/CCK-type precursors that have been identified in eleutherozoan echinoderms comprise two or three SK/CCK-type peptides (Figure 5). Informed by the phylogenetic relationships of extant echinoderms, two evolutionary scenarios could explain this difference. The occurrence of a single peptide could be the ancestral condition, with the occurrence of two or more peptides reflecting intragenic sequence duplication in the eleutherozoan lineage. Alternatively, the occurrence of two peptides could be the ancestral condition, with subsequent loss of one peptide occurring in the crinoid lineage.

##### Vasopressin/oxytocin-type precursor

The *A. mediterranea* vasopressin/oxytocin-type precursor (AmVP/OTP) is a 149-residue protein that comprises a predicted 9-residue VP/OT-type peptide (crinotocin) followed by a neurophysin domain, consistent with the structure of VP/OT-type precursors in other taxa (Odekunle and Elphick, 2020; Figure 2L). Informed by the occurrence of post-translational modifications of VP/OT peptides in other species, including the starfish *A. rubens* (Odekunle et al., 2019), crinotocin is predicted to be C-terminally amidated and to have a disulphide bridge between the two cysteine residues in the N-terminal region of the peptide. Alignment of crinotocin with VP/OT-type peptides in other echinoderms reveals that, in addition to the presence of the two conserved cysteines predicted to form a disulphide bridge, a C-terminal PxG-NH_2_ motif (where X is variable) is also a conserved characteristic (Figure 5). However, in the three crinoid species analyzed, a feature of the VP/OT-type peptide that distinguishes it from VP/OT-type peptides in eleutherozoan echinoderms is a Trp-Arg motif at positions 3 and 4.

#### Precursors of heterodimeric neuropeptides

Glycoprotein hormone-type, bursicon-type and insulin/relaxin-related peptides have been identified previously in other echinoderms (Semmens et al., 2016; Zandawala et al., 2017; Chen et al., 2019) and here we report the identification of crinoid representatives of these peptide families. These include, in *A. mediterranea*, two bursicon-alpha type precursors (Figures 3A,B), partial sequences of two bursicon-beta type precursors (Figures 3C,D), two glycoprotein hormone alpha-2 type precursors (Figures 3E,F), three glycoprotein hormone beta-5 type precursors (Figures 3G–I), an insulin-type precursor (Figure 3J) and an insulin/bombyxin-type precursor (Figure 3K). In addition, a relaxin-type precursor was identified in *A. japonica* (Figure 3L).

### Discovery of genes encoding F-type and L-type SALMFamide precursors in crinoids

The SALMFamides are a family of neuropeptides that were first discovered in starfish and that have subsequently been discovered in other echinoderms, but not in other phyla (Elphick, 2014). Therefore, the SALMFamides may be an echinoderm-specific neuropeptide family. Analysis of transcriptome/genome sequence data has revealed the occurrence of two types of SALMFamide precursor in eleutherozoan echinoderms: L-type precursors that typically contain neuropeptides with a C-terminal Leu-X-Phe-NH_2_ motif (where X is variable) and F-type precursors that typically contain neuropeptides with a C-terminal Phe-X-Phe-NH_2_ motif (where X is variable). Furthermore, analysis of the exon/intron structure of genes encoding SALMFamides has provided further evidence for the existence of two distinct families of L-type and F-type precursor proteins (Elphick et al., 2015).

A transcript encoding a protein that comprises 14 predicted SALMFamide-type neuropeptides has been identified previously in *A. mediterranea* (Elphick et al., 2015). Here we have identified orthologs of this protein in *F. serratissima* and *A. japonica* (Figure 6A). Furthermore, analysis of the structure of the gene encoding this protein in *A. japonica* revealed the presence of two phase 0 introns, with the first intron preceding the start codon encoding the N-terminal methionine of the signal peptide and the second intron located between the exon encoding the signal peptide and the exon encoding multiple SALMFamide-type peptides. Genes encoding eleutherozoan F-type SALMFamide precursors in eleutherozoan have introns in the same locations and phase (0) (Figure 6B), indicating that the *A. japonica* protein is an ortholog of eleutherozoan F-type SALMFamide precursors.

Informed by identification of putative F-type SALMFamide precursors in crinoids, we investigated the occurrence of putative L-type SALMFamide precursors in crinoids. In all three species analyzed we identified a transcript encoding a protein that comprises a peptide with the predicted C-terminal sequence GLRFHKGAPF-NH_2_ (Figure 7A). Importantly, this peptide shares sequence similarity with neuropeptides derived from L-type SALMFamide precursors in other echinoderms. For example, a neuropeptide derived from the L-type SALMFamide precursor in the starfish *A. rubens* has the sequence LHSALPF-NH_2_ (Semmens et al., 2016), with the underlined residues/amide being identical in the crinoid peptides. This suggests that the crinoid precursors of peptides with the predicted C-terminal sequence GLRFHKGAPF-NH_2_ are orthologs of eleutherozoan L-type SALMFamide precursors. Analysis of the structure of the gene encoding the putative L-type SALMFamide precursor in *A. japonica* revealed the presence of two phase 0 introns and in the same locations as the two introns in F-type SALMFamide precursor genes. This contrasts with eleutherozoan L-type SALMFamide precursor genes, which only have a single phase 0 intron located between an exon encoding the signal peptide and an exon encoding two or more L-type SALMFamide peptides (Figure 7B). Furthermore, there is a second phase 0 intron in the L-type SALMFamide precursor gene in the sea cucumber *A. japonicus* (Figure 7B), which appears to be a taxon-specific characteristic.

### Discovery of other predicted crinoid neuropeptide precursors

**FIGURE 8:**
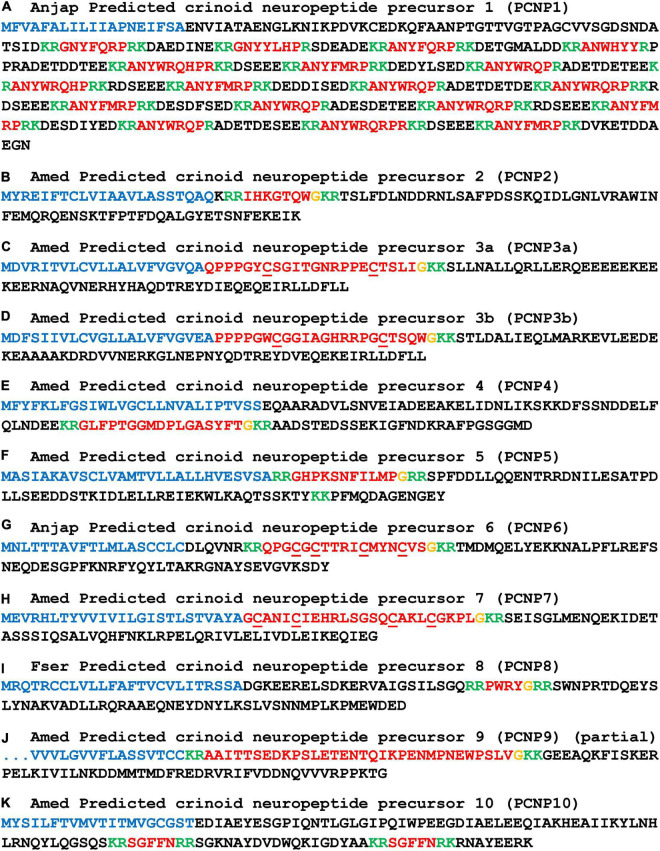
Other predicted crinoid neuropeptide precursors (PCNP1-10). Neuropeptide precursor sequences from *Antedon mediterranea* (Amed) are shown, with the exception of those precursors that were partially or not identified in this species and where sequences from *Anneissia japonica* (Anjap) or *Florometra serratissima* (Fser) are shown. The predicted signal peptide is shown in blue, predicted monobasic/dibasic cleavage sites are shown in green and predicted neuropeptides are shown in red, but with C-terminal glycine residues that are potential substrates for post-translational amidation shown in orange. The DNA sequences of transcripts encoding these precursors are shown in Supplementary File 2.

**FIGURE 9:**
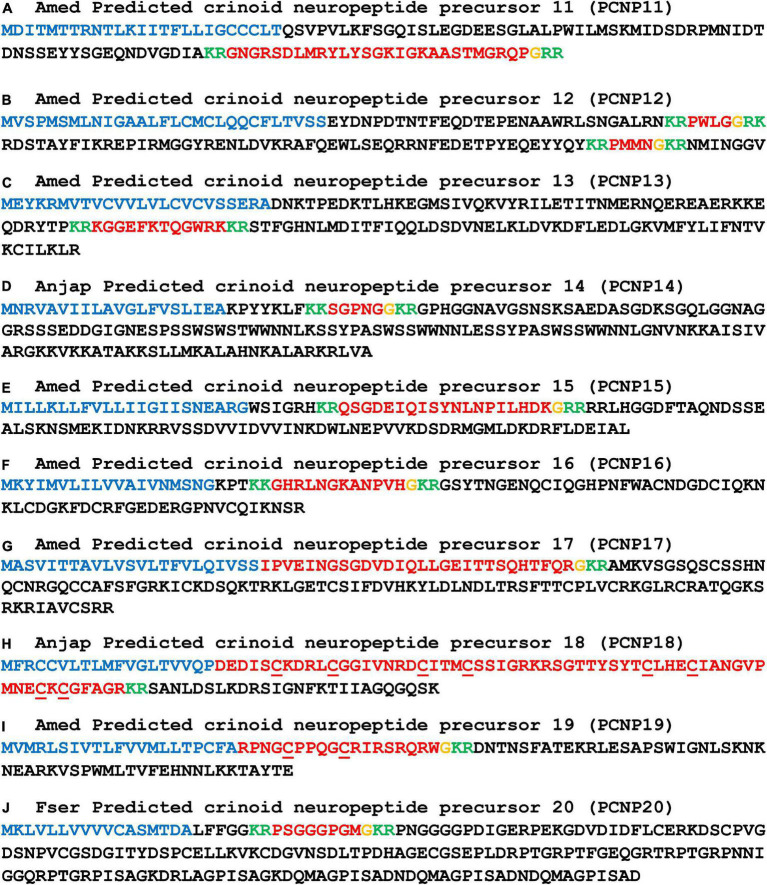
Other predicted crinoid neuropeptide precursors (PCNP11-20). Neuropeptide precursor sequences from *Antedon mediterranea* (Amed) are shown, with the exception of those precursors that were partially or not identified in this species and where sequences from *Anneissia japonica* (Anjap) or *Florometra serratissima* (Fser) are shown. The predicted signal peptide is shown in blue, predicted monobasic/dibasic cleavage sites are shown in green and predicted neuropeptides are shown in red, but with C-terminal glycine residues that are potential substrates for post-translational amidation shown in orange. The DNA sequences of transcripts encoding these precursors are shown in Supplementary File 2.

**FIGURE 10:**
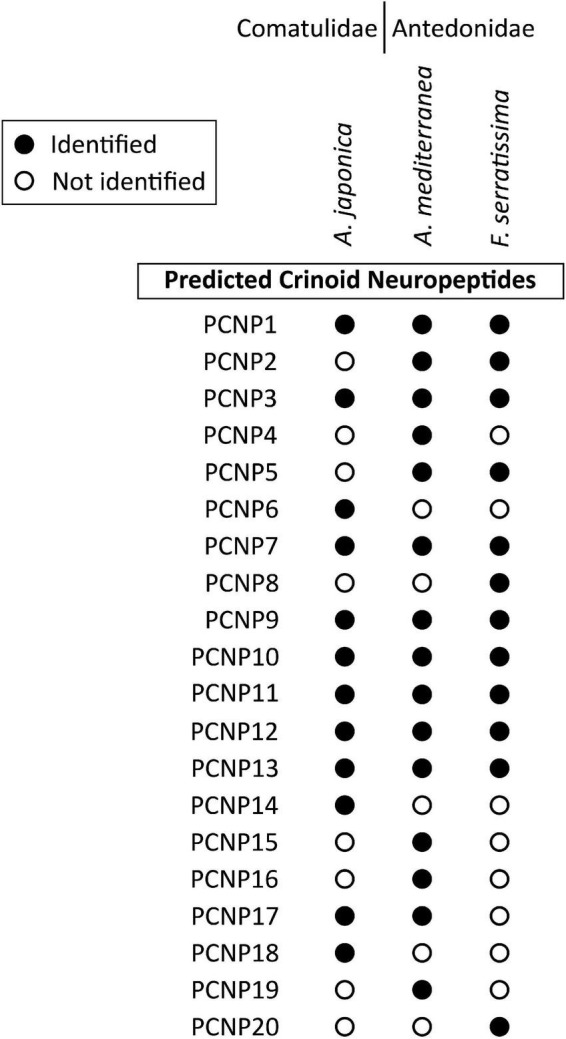
Summary showing the species in which the sequences of other predicted crinoid neuropeptide precursors (PCNP) have been identified in this study. Filled circles represent cases where neuropeptide precursors were identified in a given species, whereas empty circles represent cases in which the precursors were not identified. The DNA sequences of transcripts encoding these precursors are shown in Supplementary File 2. Multiple sequence alignments of PCNP sequences from the three crinoid species are shown in Supplementary File 3.

In addition to the neuropeptide precursors shown in Figures 2–7, and described above, we also identified other putative neuropeptide precursors in *A. mediterranea, F. serratissima*, and *A. japonica* (Figures 8–10 and Supplementary File 1). These were identified as predicted crinoid neuropeptide precursors (PCNPs) based on the following characteristics: (i) presence of a predicted N-terminal signal peptide and (ii) presence of one or more putative neuropeptide sequences bounded by predicted dibasic cleavage sites. Furthermore, several of the candidate neuropeptide precursors contained putative neuropeptide sequences with a C-terminal glycine residue, which is a potential substrate for post-translational conversion to a C-terminal amide group. Orthology of PCNPs in the three crinoid species was revealed by alignment of the precursor sequences (Supplementary File 3).

We have not identified relationships between the PCNPs and neuropeptide precursors in other phyla. However, the first of the PCNPs shown in Figure 8 is a crinoid homolog of precursors of a family of putative neuropeptides that have been identified in other echinoderms, which are known as AN peptides on account of the presence of an N-terminal Ala-Asn (AN) motif (Rowe and Elphick, 2012; Semmens et al., 2016; Zandawala et al., 2017; Chen et al., 2019). Accordingly, the *A. japonica* AN peptide precursor (AnjapPCNP1) comprises sixteen copies of putative neuropeptides with an N-terminal AN motif and two copies of putative neuropeptides with an N-terminal GN motif (Figure 8A).

For the other PNCPs we have not identified evidence of homology with neuropeptide precursors in other echinoderms. Therefore, here we restrict ourselves to a description of the putative neuropeptides derived from PCNP2-PCNP20. AmedPCNP2 comprises a single putative C-terminally amidated neuropeptide (IHKGTQW-NH_2_), which is located after the N-terminal signal peptide but separated from it by a predicted tribasic cleavage site (Figure 8B). AmedPCNP3a and AmedPCNP3b comprise structurally related putative C-terminally amidated neuropeptides that contain two cysteine residues, which may form an intramolecular disulphide bridge in the mature peptide (Figures 8C,D). AmedPCNP4 comprises a single putative C-terminally amidated neuropeptide GLFPTGGMDPLGASYFT-NH_2_ (Figure 8E). AmedPCNP5 comprises a single putative C-terminally amidated neuropeptide (GHPKSNFILMP-NH_2_), which is located after the N-terminal signal peptide but separated from it by a predicted dibasic cleavage site (Figure 8F). AnjapPCNP6 and AmedPCNP7 both comprise a putative C-terminally amidated neuropeptide that contains four cysteine residues, which may form two intramolecular disulphide bridges in the mature peptide, but differences in the sequences and the spacing of the cysteines residues in these two proteins suggests that they are not homologs (Figures 8G,H). AmedPCNP8 comprises a putative neuropeptide with the predicted structure PWRY-NH_2_ (Figure 8I). AmedPCNP9 is partial sequence because it lacks an N-terminal methionine, but it comprises a putative C-terminally amidated thirty-two residue neuropeptide (Figure 8J). AmedPCNP10 comprises two copies of a putative neuropeptide with the amino-acid sequence SGFFN (Figure 8K).

AmedPCNP11 comprises a putative C-terminally amidated twenty-seven residue neuropeptide located in the C-terminal region of the precursor protein (Figure 9A). AmedPCNP12 comprises two putative C-terminally amidated neuropeptides—PWLG-NH_2_ and PMMN-NH_2_ (Figure 9B). AmedPCNP13 comprises a putative neuropeptide with the amino-acid sequence KGGEFKTQGWRK (Figure 9C). AmedPCNP14 comprises a putative C-terminally amidated neuropeptide (SGPNG-NH_2_) (Figure 9D). AmedPCNP15 comprises a putative C-terminally amidated neuropeptide (QSGDEIQISYNLNPILHDK-NH_2_) (Figure 9E). AmedPCNP16 comprises a putative C-terminally amidated neuropeptide (GHRLNGKANPVH-NH_2_) (Figure 9F). AmedPCNP17 comprises a putative 28 residue C-terminally amidated neuropeptide, immediately after the signal peptide (Figure 9G). AmedPCNP18 comprises a putative C-terminally amidated 60-residue neuropeptide with 8 cysteine residues, which may form intramolecular or intermolecular disulphide bridges (Figure 9H). AmedPCNP19 comprises a putative C-terminally amidated neuropeptide with 2 cysteine residues, which may form an intramolecular disulphide bridge in the mature peptide (Figure 8I). AmedPCNP20 comprises a putative C-terminally amidated neuropeptide (PSGGGPGM-NH_2_; Figure 9J).

### Localization of F-type SALMFamide precursor expression in *Antedon mediterranea* using mRNA *in situ* hybridization

The expression of SALMFamide-type neuropeptides in crinoids has been investigated previously using antibodies to the starfish SALMFamide neuropeptides S1 and S2 (Elphick et al., 1991b, 1995; Moore and Thorndyke, 1993; Heinzeller and Welsch, 1994; Bonasoro et al., 1995; Candia Carnevali et al., 1998). However, the specificity of these heterologous antibodies when tested on crinoids is unknown. Therefore, here we used mRNA *in situ* hybridization methods to specifically investigate F-type SALMFamide precursor expression in *A. mediterranea*. This was facilitated by the previously reported cloning and sequencing of a cDNA encoding the *A. mediterranea* F-type SALMFamide precursor (Elphick et al., 2015), which was used as a template for production of DIG-labeled anti-sense RNA probes. Furthermore, F-type SALMFamide precursor expression was investigated at three stages of the *A. mediterranea* life cycle: (1) free-swimming doliolaria larval stage, (2) post-metamorphic sessile pentacrinoid juvenile stage and (3) free-swimming adult stage. Detailed morphological descriptions of these three stages of the *A. mediterranea* life cycle have been reported previously (Candia Carnevali et al., 1996; Candia Carnevali and Bonasoro, 2001; Barbaglio et al., 2012; Mercurio et al., 2019).

**FIGURE 11:**
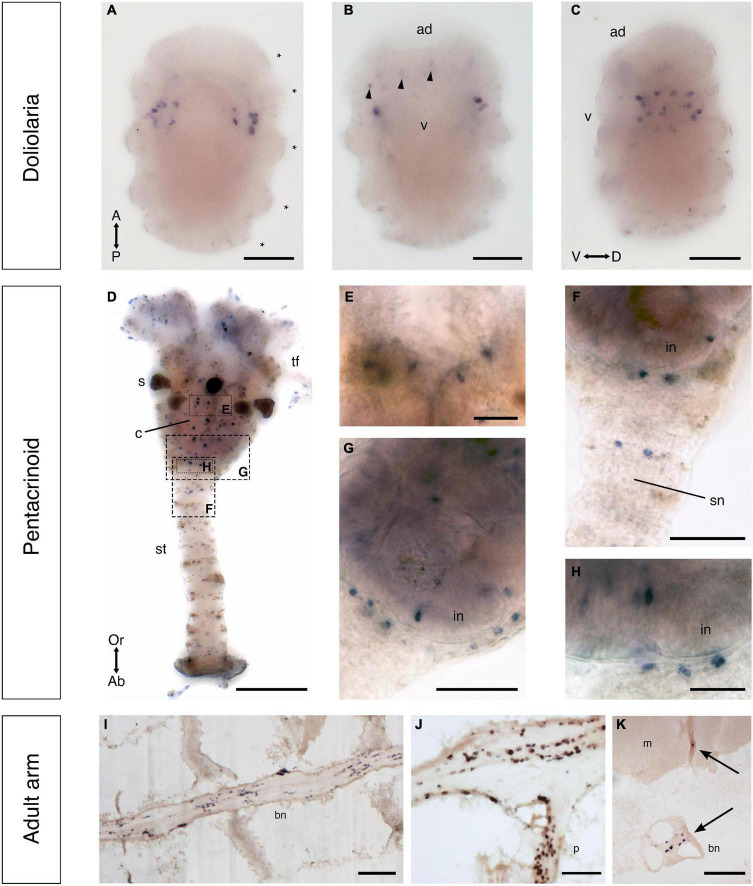
F-type SALMFamide precursor expression across different life stages of *Antedon mediterranea* revealed using mRNA *in situ* hybridization. **(A–C)** Doliolaria larva from dorsal **(A)**, ventral **(B)**, and lateral **(C)** views, showing strong staining in two symmetric dorso-lateral clusters of cells located between the second and third ciliary bands (asterisks) and weak staining in sparsely distributed cells located in an anterior-ventral position (arrowheads in panel **B**). **(D–H)** A pentacrinoid juvenile **(D)**, showing stained cells in both the calyx and stalk. High magnification images of the boxed regions in **(D)** show stained cells in mouth **(E)**, stalk nerve **(F)** and aboral nerve center **(G,H)**. **(I–K)** Longitudinal **(I,J)** and transverse **(K)** sections of arms from adult animals, revealing stained cells in the brachial nerve **(I)**, branches of the brachial nerve that project into the pinnules **(J)** and flexor muscles **(K)**. Scale bars: 150 μm for **(A–D,I)**; 75 μm for **(F,G,J,K)**; 25 μm for **(E,H)**. A, anterior side; Ab, aboral side; ad, adhesive pit; bn, brachial nerve; c, calyx; D, dorsal side; m, muscle; in, intestine; Or, oral side; P, posterior side; p, pinnule; s, saccules, sn, stalk nerve; st, stalk; tf, tube feet; V, ventral side; v, vestibulum.

Whole-mount staining methods were employed to visualize F-type SALMFamide precursor transcripts in the doliolaria larval stage, revealing bilaterally symmetrical clusters of strongly stained cells located between the second and third ciliary bands (Figures 11A–C). The specificity of this staining was confirmed by the absence of staining in these cells in larvae incubated with sense probes (Supplementary Figure 3A). Visualization of stained larvae from dorsal (Figure 11A), ventral (Figure 11B), and lateral (Figure 11C) views revealed that these cells are located laterally, extending from the ventral to the dorsal sides of the larvae. In addition to these strongly stained cells, a dispersed group of weakly stained cells is located anteriorly, close to the ventral surface between the adhesive pit and the vestibule (Figure 11B). These cells are located deep within the epidermis, corresponding to the position of the basiepithelial nerve plexus described previously in this larval stage (Mercurio et al., 2019).

Whole-mount staining methods were also employed to visualize F-type SALMFamide precursor transcripts in pentacrinoids and stained cells were revealed to be widely distributed in both the calyx and stalk (Figures 11D–H). In the calyx, labeled cells were revealed adjacent to the mouth and in the intestine, but no staining was observed in the ectoneural system associated with tube feet (Figures 11D,E). Staining in the saccules of the calyx was revealed to be non-specific because it is also observed with sense probes (Supplementary Figures 3E,F). In the stalk, staining was observed in a pair of cells in the stalk nerve (Figure 11F) and in the aboral nerve center (ANC) just below the intestine wall (Figures 11G,H).

To visualize F-type SALMFamide precursor transcripts in adult animals, sections of arms were analyzed. Staining was observed in all the major compartments of the entoneural nervous system (Figures 11I–K), including the brachial nerve, which runs aborally along the arm (Figure 11I), branches of the brachial nerve in the pinnules (Figure 11J) and in nerves proximal to the flexor muscles of the arm (arrows in Figure 11K). The distribution of these stained cells along the length of the brachial nerve is consistent with the positions of neuronal cell bodies, as shown by light and electron microscopy (Heinzeller and Welsch, 1994). Furthermore, the specificity of staining observed in sections of adult arms in tests with antisense probes (Figures 11I–K) was confirmed by an absence of this staining in sections of adult arms tested with sense probes (Supplementary Figures 3B,C).

### Localization of the expression of a calcitonin-type neuropeptide in *Antedon mediterranea*

Immunohistochemical analysis of neuropeptide expression in crinoids has been accomplished previously using antibodies to the starfish SALMFamide neuropeptide S2 (Heinzeller and Welsch, 1994). However, a complexity associated with immunohistochemical analysis of SALMFamide expression in echinoderms is the occurrence of two types of SALMFamide precursor that typically comprise multiple copies of structurally related peptides (Elphick et al., 2015), which makes interpretation of the specificity of immunostaining difficult. Therefore, having identified in this study many novel neuropeptide precursors in crinoids (see above), we investigated opportunities for analysis of the expression of neuropeptides derived from precursor proteins that comprise a single neuropeptide. Furthermore, we analyzed sequence data to identify neuropeptides in *A. mediterranea* that share a high level of sequence similarity with peptide antigens that have been used previously for production of antibodies to neuropeptides in the starfish *A. rubens*. This revealed that the C-terminal region of the *A. mediterranea* calcitonin-type neuropeptide AmCT (GGM**FG**S**SGP-NH_2_**) shares sequence similarity (in bold) with the C-terminal region of the *A. rubens* calcitonin-type neuropeptide ArCT (NSP**FG**A**SGP-NH_2_**), which has been used previously as an antigen for generation of antibodies that have been used for immunohistochemical visualization of ArCT expression in *A. rubens* (Cai et al., 2018).

**FIGURE 12:**
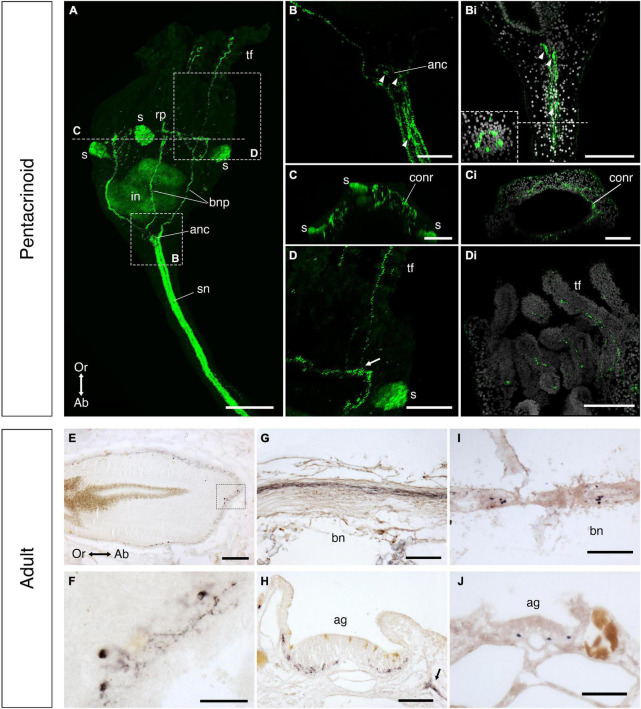
Localization of calcitonin-type neuropeptide expression in post-metamorphic stages of *Antedon mediterranea*. **(A–Di)** Immunofluorescent labeling of calcitonin-type neuropeptide expression in the pentacrinoid stage using an antiserum to the *A. rubens* calcitonin-type neuropeptide ArCT. Immunolabeling is observed in the calyx, stalk, and tube feet **(A)**. High magnification images show five tracts containing strongly labeled fibers in the stalk nerve **(B,Bi)**, and fibers and cell bodies (arrowheads) in the aboral nerve center **(B,Bi)**, from which five immunolabeled brachial nerve primordia project orally **(A,B)** to the circumoral nerve ring [**(A)**, resliced for oral view in panel **(C,Ci)**]. Labeled fibers in the tube feet **(D,Di)** appear to originate in the circumoral nerve ring [white arrow in panel **(D)**]. **(E,F)** Localization of calcitonin-type neuropeptide expression in sections of the adult calyx **(E,F)** and arm **(G,H)** as revealed by immunohistochemistry using an antiserum to the *A. rubens* neuropeptide ArCT **(E–H)** and by mRNA *in situ* hybridization using probes for *A. mediterranea* calcitonin-type precursor (AmCT) transcripts **(I,J)**. In the calyx, immunostaining is located at the base of the mucosa of the esophagus **(E)**, where high magnification images show staining located in cell bodies and fibers **(F)**. In a sagittal section of an arm (oral side uppermost), immunostained fibers can be seen running longitudinally in the brachial nerve **(G)**. In a transverse section of an arm (oral side uppermost), immunostained cells/fibers can be seen in the ectoneural region of the nervous system at the base of the ambulacral groove **(H)** and in fibers located aboro-laterally to the ambulacral groove (black arrow in panel **H**), which may be derived from hyponeural neurons. Consistent with immunohistochemical analysis of calcitonin-type neuropeptide expression, mRNA *in situ* hybridization reveals AmCT precursor transcripts in cells located in the brachial nerve **(I)** (oral side uppermost) and at the base of the ectoneural region of the ambulacral groove **(J)** (oral side uppermost). Scale bar is 100 μm for **(A,E,G,I)**; 75 μm for **(B–Di,H,J)**; 25 μm for **(F)**. Ab, aboral side; ag, ambulacral groove; anc, aboral nerve center; bn, brachial nerve; conr, circumoral nerve ring; in, intestine; bnp, brachial nerve primordia; Or, oral side; rp, radial plates; s, saccules; sn, stalk nerve; tf, tube foot.

We first tested the ArCT antiserum (dilution 1:3,000) on whole-mount preparations of pentacrinoid juveniles using immunofluorescence methods and this revealed a striking pattern of labeling. Previous studies have reported that non-specific autofluorescence is observed in the intestine and saccules of pentacrinoids (Mercurio et al., 2019), and accordingly we also observed fluorescence in these structures (Figure 12A). Furthermore, comparison of pentacrinoids that had been incubated with the ArCT antiserum and pentacrinoids that had been incubated with ArCT antiserum pre-absorbed with the ArCT antigen peptide (Supplementary Figure 3D) established the specificity of intense immunolabeling of nerve fibers in the stalk nerve and in the ANC at the base of the calyx, where a few immunolabeled cell bodies were also visible (Figures 12A,Bi). From the ANC, five groups of labeled fibers extend orally to the radial plates (Figure 12A) and we refer to the nerves containing these labeled fibers as *brachial primordia*. At the level of the brachial plates, the brachial primordia coalesce with other immunolabeled fibers in a circumoral nerve ring, which is visible from an oral view in Figures 12C,Ci. Immunolabeled fibers also extend from the circumoral nerve ring into the tube feet (Figures 12D,Di).

In adult animals, the ArCT antiserum was used for immunohistochemical staining of sections of the calyx and arms. In the calyx, immunostaining was observed in the esophagus (Figure 12E). High magnification images of the esophagus revealed immunostained cell bodies positioned at the base of the wall of the esophagus with processes extending into a basiepithelial nerve plexus (Figure 12F). In the arms, labeled fibers were revealed in the brachial nerve (Figure 12G) and cells with short processes were observed in the aboral region of the ambulacral groove (Figure 12H). Furthermore, immunostaining was also observed in fibers located aboro-laterally to the ambulacral groove (arrow in Figure 12H). In parallel with use of immunohistochemical staining methods, we also used mRNA *in situ* hybridization to analyze expression of AmCT precursor transcripts in arms. Consistent with the patterns of immunostaining observed with antibodies to ArCT (Figures 12E–H), AmCT precursor transcripts were revealed along the brachial nerve where entoneural neurons are positioned (Figure 12I) and in cells at the base of the ambulacral groove (Figure 12J), which is where the ectoneural nervous plexus is located (Heinzeller and Welsch, 1994).

### The vasopressin/oxytocin-type neuropeptide crinotocin is expressed in the brachial nerve of *Antedon mediterranea* and affects the mechanical behavior of arm preparations

We selected the vasopressin/oxytocin-type peptide crinotocin for experimental analysis of neuropeptide function in *A. mediterranea* because i) it is a small, single-copy neuropeptide that belongs to a family of peptides that typically have a highly conserved structure in bilaterian animals (Odekunle and Elphick, 2020), which facilitated synthesis of this peptide, and ii) our previous studies on an orthologous peptide in the starfish *A. rubens* (asterotocin) have revealed striking *in vitro* and *in vivo* pharmacological effects on muscle preparations and behavior, respectively (Odekunle et al., 2019).

**FIGURE 13:**
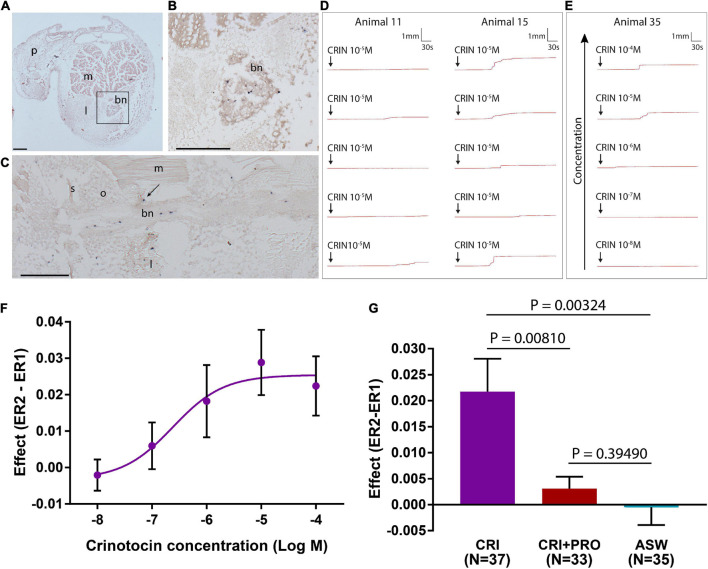
Crinotocin precursor expression in *Antedon mediterranea* and *in vitro* effects of crinotocin on arm preparations. **(A–C)** Arm sections of *A. mediterranea* labeled with antisense probes for crinotocin precursor transcripts using mRNA *in situ* hybridization. bn, brachial nerve; l, ligament; m, muscle; p, pinnule; o, ossicle. s, syzygy; Scale bars: 120 μm for **(A,B)**; 210 μm for **(C)**. **(A)** Transverse section of the arm, with the square showing the region containing the brachial nerve, which is shown at higher magnification in panel **(B)**. **(B)** High magnification image of the brachial nerve, showing the presence of crinotocin precursor-expressing cells in the core and margins of the nerve. **(C)** Sagittal section of an arm, showing labeled cells in the brachial nerve along its length and in branches of the brachial nerve that innervate ligaments and muscles (arrow). **(D)** Effects of crinotocin (10^–5^ M) on arm preparations, with five tests on different preparations from two representative animals shown. After addition of crinotocin (arrow), recording was continued for several minutes. Variability in responses both within and between animals is observed. For example, with animal 11 crinotocin caused a gradual and small flexion in some arm preparations, whereas in most animals (e.g., animal 15) crinotocin caused an abrupt and large flexion in most arm preparations. **(E)** Concentration-dependent effect of crinotocin on mean arm flexion rate. Five different concentrations were tested (10^–4^ M, 10^–5^ M, 10^–6^ M, 10^–7^ M, 10^–8^ M), and an example test per concentration for a representative animal is shown. Responses are greatest at the highest concentrations tested (10^–4^ M and 10^–5^ M), and visibly decrease at intermediate concentrations until little or no visible effect is observed at the lowest concentrations tested (10^–7^ M and 10^–8^ M). **(F)** Concentration-response curve for the effect of crinotocin on arm preparations. Data from 143 arm preparations from four animals were used to generate this concentration-response curve, but one data point for the 10^–4^ M concentration was excluded as an outlier. See Supplementary File 5. Error bars represent standard error of the mean (SEM). The graph was generated using GraphPad Prism. **(G)** Anesthetization of arm preparations blocks the response of arms to crinotocin. ASW: pretreated in ASW alone then treated with ASW alone; CRIN: pretreated in ASW alone then treated with 10^–5^ M crinotocin; CRIN+PRO: anesthetized in 10^–3^ M procaine hydrochloride then treated with 10^–5^ M crinotocin in anesthetic solution. Numbers of preparations are shown in parentheses. Error bars represent SEM. The graph was generated using GraphPad Prism.

As a prelude to pharmacological experiments, the expression of the crinotocin precursor in arms of adult *A. mediterranea* was first investigated using mRNA *in situ* hybridization. Cells expressing crinotocin precursor transcripts were revealed in the brachial nerve of arms (Figures 13A–C), with longitudinal sections of arms (Figure 13C) revealing stained cells along the length of the nerve either singularly or in small and sparse clusters, corresponding with previous descriptions of the locations of neuronal cell bodies (Heinzeller and Welsch, 1994). Stained cells were also observed at the base of brachial nerve branches that innervate the muscles and ligaments of the arm (Figure 13C). Accordingly, in transverse sections of arms labeled cells were observed both in cells at the center of the brachial nerve (the “medulla”) and in the external perikarya-rich “cortex” of the nerve (Figures 13A,B; and for details of brachial nerve anatomy, see Candia Carnevali et al., 1996). The specificity of staining observed with anti-sense probes was confirmed by an absence of staining in the nervous system in tests with sense probes (Supplementary Figures 3E,F).

Informed by these findings, we then investigated the pharmacological effects of crinotocin on arm preparations from *A. mediterranea*. The structure of the crinotocin peptide that was synthesized for these experiments (CFWRTCPVG-NH_2_, with a disulphide bridge between the pair of cysteine residues) was informed by the sequence of the crinotocin precursor protein, based on transcriptome sequence data, and known and highly conserved post-translational modifications of vasopressin/oxytocin-type neuropeptides in other taxa (Odekunle and Elphick, 2020).

Using an experimental method that has been employed previously for investigation of the effects of neurotransmitters on the mechanical properties of arm preparations from *A. mediterranea* (Wilkie et al., 2010) we observed that 10^–5^ M crinotocin administration was followed by a significant increase in the mean rate of extension (i.e., upward bending) of the preparations in comparison with ASW-treated controls (*P* = 0.00002) (Table 1). Crinotocin usually caused single or multiple abrupt but transient increases in bending rate, although in a minority of preparations there was a gradual acceleration of the bending rate (Figure 13D). To investigate the specificity of the effect of crinotocin, three other echinoderm neuropeptides (*A. rubens* SALMFamide S1 and S2 and *A. rubens* luqin-type peptide ArLQ) were also tested on arm preparations. These experiments showed that the effect was unique to the *Antedon*-specific neuropeptide crinotocin, as none of the starfish neuropeptides had a discernible effect on the bending rate of the preparations (Table 1, Supplementary Table 2, and Supplementary File 4). An experiment testing a range of crinotocin concentrations (from 10^–4^ to 10^–8^ M) showed that the effect of crinotocin is dose-dependent (Figures 13E,F and Supplementary File 5). The large error bars of the dose-response graphs reflect the highly variable nature of these biological samples, but the trend is clear. Furthermore, to gain insights into the mechanism of action of crinotocin we tested the anesthetic (sodium channel blocker) procaine hydrochloride (10^–3^ M), which blocked the effect of crinotocin (Figure 13G and Supplementary File 6), suggesting that the effect of crinotocin is mediated by excitable cells.

**TABLE 1:** Response of *Antedon mediterranea* arm preparations to crinotocin and other neuropeptides.

		ER2-ER1		
	*N*	Range	Mean (SEM)	*P*
**Demonstration of crinotocin effect**				
ASW	34	−0.048 to 0.034	−0.001 (0.003)	
Crinotocin	47	−0.024 to 0.262	0.055 (0.010)	0.00002
**Comparison with other neuropeptides**				
ASW	13	−0.048 to 0.014	−0.011 (0.004)	
Crinotocin	11	−0.024 to 0.084	0.022 (0.011)	0.01660
S1	12	−0.046 to 0.032	−0.011 (0.006)	0.92313
S2	13	−0.092 to 0.006	−0.011 (0.008)	0.93199
ArLQ	13	−0.068 to 0.026	−0.008 (0.006)	0.75518

Artificial sea water (ASW) is the negative control. N, number of samples; ER2-ER1, the effect (see main text); *P*, *p*-value of treatment vs. negative control (see also Supplementary File 4).

## Discussion

**FIGURE 14:**
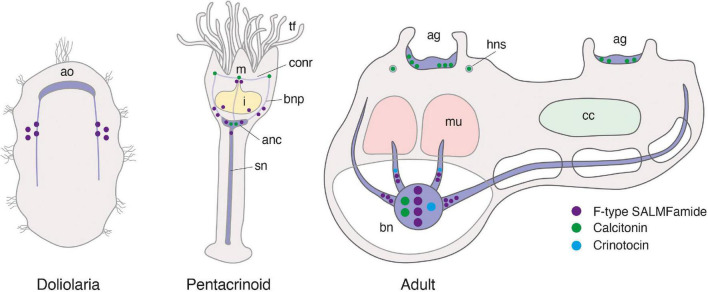
Summary of neuropeptide expression in *Antedon mediterranea*. The distribution of cells expressing F-type SALMFamide (purple, mRNA *in situ*); calcitonin-type neuropeptide (green, immunohistochemistry and mRNA *in situ*); and crinotocin (cyan, mRNA *in situ*) is summarized here for doliolaria larva, pentacrinoid and adult stages and related to the major compartments of the nervous system (blue). ag, ambulacral groove; anc, aboral nerve center; ao, apical organ; bn, brachial nerve; bnp, brachial nerve primordia; cc, coelomic canal; conr, circumoral nerve ring; hns, hyponeural system i, intestine; m, mouth; mu, muscles; sn, stalk nerve; tf, tube feet.

Here we report the first extensive molecular, anatomical and functional characterization of neuropeptides in crinoid echinoderms. As discussed in detail below, our findings provide new insights into neuropeptide evolution in echinoderms. Furthermore, our anatomical analysis of neuropeptide expression in the crinoid *A. mediterranea* has revealed hitherto unknown features of the architecture of crinoid nervous systems (Figure 14). Lastly, by testing the *in vitro* pharmacological effects of the vasopressin/oxytocin-type peptide crinotocin on *A. mediterranea*, we have obtained the first insights into the physiological roles of neuropeptides in crinoids.

### Discovery of crinoid representatives of known neuropeptide families

Identification of transcripts/genes encoding neuropeptide precursors in crinoids complements previous studies that have identified neuropeptide precursors in eleutherozoan echinoderms—the Asterozoa and Echinozoa (Rowe and Elphick, 2012; Rowe et al., 2014; Semmens et al., 2016; Smith et al., 2017; Zandawala et al., 2017; Suwansa-Ard et al., 2018; Chen et al., 2019). Because the class Crinoidea is a sister group to the Eleutherozoa (Telford et al., 2014), we are now able to reconstruct the evolution of a variety of neuropeptide types in extant echinoderms. All the neuropeptides under consideration here belong to ancient neuropeptide families whose evolutionary origins have been traced to the common ancestor of deuterostomes (e.g., melanin-concentrating hormone; Semmens et al., 2016) or bilaterians (e.g., calcitonin and vasopressin/oxytocin; Mirabeau and Joly, 2013). However, we have divided our analysis and discussion of these neuropeptides into two sections. First, we consider neuropeptides that are monomeric peptides, which are typically relatively short peptides. Second, we consider neuropeptides that are heterodimeric peptides, which are larger than the monomeric neuropeptides.

#### Monomeric neuropeptides

Analysis of the transcriptome/genome sequence data enabled identification of precursors of crinoid homologs of monomeric neuropeptides that have been identified previously in eleutherozoans, including calcitonin-type, eclosion hormone-type, kisspeptin-type, luqin-type, melanin-concentrating hormone (MCH)-type, orexin-type, pedal peptide/orcokinin-type, prolactin-releasing peptide(PrRP)/short neuropeptide-F(sNPF)-type, sulfakinin/cholecystokinin-type and vasopressin/oxytocin-type (crinotocin) neuropeptides. Analysis of the sequences of these peptides enabled identification of a calcitonin-type peptide in *A. mediterranea* (AmCT) that exhibits cross-reactivity with antibodies to a calcitonin-type peptide from the starfish *A. rubens* (ArCT; Cai et al., 2018), facilitating investigation of its expression in *A. mediterranea* and revealing new insights into the architecture of crinoid nervous systems, as discussed in detail below. Furthermore, analysis of the sequences of crinoid neuropeptides also provided some interesting new insights into neuropeptide evolution.

Two kisspeptin-type precursors (KPP1 and KPP2) have been identified in eleutherozoan echinoderms and both precursors comprise two kisspeptin-like peptides (Semmens et al., 2016; Escudero Castelán et al., 2022). Here we report for the first time the identification KPP1-type and KPP2-type precursors in crinoids. Analysis of the sequences of KPP1-type precursors in eleutherozoans has revealed the presence of two predicted neuropeptides, which we refer to as KP1.1 and KP1.2 (Escudero Castelán et al., 2022). KP1.1-type peptides are preceded by an amino acid sequence that contains two cysteine residues, which was originally thought to form the N-terminal region of the mature bioactive peptide (Semmens et al., 2016). However, mass spectroscopic analysis of radial nerve cord extracts from the starfish *A. rubens* has revealed that the two cysteine residues do not form part of the mature ArKP1.1 peptide because precursor cleavage occurs at an arginine residue located C-terminal to the second cysteine residue (Escudero Castelán et al., 2022). The KPP1-type precursor in crinoids also contains this pair of cysteine residues and these are followed by an amino acid sequence containing five basic amino acids (LRIKKKPK), which are potential cleavage sites. Informed by the structure of ArKP1.1 in *A. rubens* and mechanisms of neuropeptide cleavage characterized in other phyla (Veenstra, 2000), we predict that the mature structure of AmKP1.1 is pQTSSCSHNACLRILPF-NH_2_, with the N-terminal glutamine being a potential substrate for conversion to pyroglutamate (pQ). A notable feature of AmKP1.1 and the KP1.1-type peptides in *F. serratissima* and *A. japonica* that distinguishes them from KP1.1-type peptides in eleutherozoans is the presence of the two cysteine residues within the predicted mature neuropeptide. This is interesting because it is not a characteristic of other kisspeptin-type peptides in echinoderms or vertebrates and therefore it appears to be a unique feature of KP1.1-type peptides in crinoids. The presence of two cysteine residues is, however, a feature of other neuropeptide-types in the Bilateria; for example vasopressin/oxytocin-type (Odekunle and Elphick, 2020) and somatostatin/allatostatin-C-type neuropeptides (Zhang et al., 2022). Furthermore, in these peptides the pair of cysteines form a disulphide bridge in the mature bioactive neuropeptides (Odekunle and Elphick, 2020; Zhang et al., 2022). Therefore, it seems likely that the pair of cysteine residues in AmKP1.1 also form a disulphide bridge. The discovery of a kisspeptin-type peptide in crinoids that is predicted to contain a disulphide bridge is interesting from an evolutionary perspective because the receptors that mediate effects of kisspeptin-type neuropeptides are closely related to somatostatin/allatostatin-C-type receptors (Mirabeau and Joly, 2013). Thus, we speculate that an ancestral neuropeptide signaling system that gave rise to both the somatostatin/allatostatin-C-type and kisspeptin-type signaling systems would have comprised neuropeptides that have a disulphide bridge. This characteristic was then retained in somatostatin/allatostatin-C-type neuropeptides in extant bilaterians and, if our hypothesis is correct, appears to have also been uniquely retained in one of the kisspeptin-type neuropeptides in crinoids but lost in other bilaterian kisspeptin-type peptides.

Analysis of the sequences of KPP2-type precursors in eleutherozoans has revealed the presence of two predicted neuropeptides, which we refer to as KP2.1 and KP2.2 (Escudero Castelán et al., 2022). Interestingly, the KPP2-type precursors in crinoids comprise a single putative kisspeptin-like peptide, which shares sequence similarity with eleutherozoan KP2.1-type and KP2.2 type peptides. Therefore, the KPP2-type precursor in the common ancestor of extant echinoderms may have likewise comprised a single KP2-type peptide and then intragenic duplication may have given rise to KPP2-type precursors in eleutherozoan comprising two KP2-type peptides. Alternatively, the KPP2-type precursor in the common ancestor of extant echinoderms may have comprised two KP2-type peptides, as in eleutherozoans, with loss of one peptide then occurring in the crinoid lineage.

Comparison of the sequences of other predicted monomeric neuropeptides in crinoids with their eleutherozoan counterparts revealed characteristics consistent with the phylogenetic position of crinoids as a sister group to the Eleutherozoa. Thus, sequence motifs that distinguish crinoid neuropeptides from their eleutherozoan homologs were identified in luqin-type, MCH-type, PrRP-type and vasopressin/oxytocin-type (crinotocin) peptides. Furthermore, evidence of lineage-specific duplication of neuropeptide precursor genes was also obtained. Thus, the occurrence of two luqin-type neuropeptide precursors in *A. mediterranea* appears to be distinctive characteristic of this species because in all other echinoderm species analyzed so far, including the crinoids *F. serratissima* and *A. japonica*, only a single luqin-type precursor has been identified. Conversely, only one orexin-type precursor was identified in *A. mediterranea*, whereas in eleutherozoan species there are two orexin-type precursors, which we infer therefore may have arisen by gene duplication in a common ancestor of the Eleutherozoa.

#### Heterodimeric neuropeptides

Some neuropeptides or peptide hormones are heterodimers with multiple intramolecular and/or intermolecular disulphide bridges between cysteine residues and these include glycoprotein hormones, bursicons and insulin/relaxin-related peptides. Furthermore, many of these peptide signaling molecules exert their effects by binding to receptors that are members of a family of leucine-rich repeat containing G-protein coupled receptors, which are distinct from the rhodopsin-related receptors and secretin-type receptors that mediate effects of the monomeric neuropeptides (Van Loy et al., 2008; Mirabeau and Joly, 2013). Currently, little is known about the physiological roles of glycoprotein hormone-type, bursicon-type and insulin/bombyxin-type peptides in echinoderms and our knowledge is largely restricted to sequence data (Semmens et al., 2016; Zandawala et al., 2017; Chen et al., 2019). Accordingly, here we report the sequences of precursors of these neuropeptides in *A. mediterranea.* However, in contrast to the paucity of functional insights on the aforementioned neuropeptide types, a substantial body of knowledge has been obtained on the physiological roles of relaxin-type peptides in starfish species. Thus, a relaxin-type peptide has been shown to act as a gonadotropic peptide in starfish and accordingly it is known as relaxin-like gonad-stimulating peptide (RGP) (Mita et al., 2009; Mita, 2013). Furthermore, it has recently been reported that a relaxin-type peptide triggers spawning in the sea cucumber *Holothuria scabra* (Chieu et al., 2019). Therefore, it appears that relaxin-type peptides may have a general role as gonadotropins in echinoderms. With this hypothesis in mind, it is noteworthy that in this paper we report for the first time the identification of a precursor of a relaxin-type peptide in a crinoid (*A. japonica*). This provides a basis for studies in which the effects of relaxin-type peptides as putative regulators of spawning in crinoids could be investigated experimentally.

### Discovery of L-type and F-type SALMFamide precursors in crinoids provides new insights into the evolution of SALMFamide neuropeptides in the phylum Echinodermata

Analysis of transcriptome/genome sequence data has revealed the presence of two types of precursors of SALMFamide neuropeptides in eleutherozoan echinoderms—L-type and F-type precursors. L-type precursors give rise to two or more neuropeptides that typically have a C-terminal Leu-X-Phe-NH_2_ motif (where X is variable). F-type precursors give rise to seven or more neuropeptides that typically have a C-terminal Phe-X-Phe-NH_2_ motif (where X is variable) (Elphick et al., 2015). Analysis of transcriptome sequence data combined with cDNA cloning and sequencing previously enabled identification of SALMFamide-type neuropeptide precursor in *A. mediterranea* that comprises fourteen putative SALMFamides (Elphick et al., 2015). Because this precursor comprises some peptides with a C-terminal Phe-X-Phe-NH_2_ motif and some peptides with a C-terminal Leu-X-Phe-NH_2_ motif, it was suggested that it may represent an ancestral-type SALMFamide precursor, pre-dating a gene duplication event that gave rise to genes encoding L-type and F-type SALMFamide precursors in eleutherozoan echinoderms. Alternatively, because the SALMFamide precursor in *A. mediterranea* generally shares more similarity with F-type precursors than L-type precursors, an alternative hypothesis proposed is that it is an F-type precursor and that an L-type precursor remained to be discovered in crinoids (Elphick et al., 2015).

Analysis of the intron/exon structure of genes encoding L-type and F-type SALMFamide precursors in eleutherozoan echinoderms has revealed both similarities and differences in intron position. Thus, genes encoding both precursor types in eleutherozoans have a phase 0 intron located between an exon that encodes the N-terminal signal peptide and an exon that encodes SALMFamide neuropeptides, as shown in this study (Figures 6, 7) and previously (Elphick et al., 2015). However, the presence of another phase 0 intron that precedes the start codon in eleutherozoan F-type SALMFamide precursor genes distinguishes these genes from eleutherozoan L-type SALMFamide precursor genes, which do not have an intron in this position, as shown in this study and previously (Elphick et al., 2015). Here we report for the first time the discovery of putative L-type SALMFamide precursors in crinoids, providing evidence that the SALMFamide precursor previously identified in *A. mediterranea* (Elphick et al., 2015), and here in two other crinoid species, is an F-type precursor. Interestingly, both SALMFamide precursor genes in the crinoid *A. japonica* have two phase 0 introns and in the same positions as in eleutherozoan F-type SALMFamide precursor genes. Therefore, gene structure does not provide a basis for distinguishing F-type and L-type SALMFamide precursor genes in crinoids. However, similarities in the sequences of the candidate neuropeptides derived from putative crinoid L-type SALMFamide precursors and eleutherozoan L-type SALMFamide precursor derived neuropeptides provide a basis for ascribing orthology. Thus, a C-terminal Pro-Phe-NH_2_ motif is a feature of the predicted neuropeptides derived from the putative crinoid L-type SALMFamide precursors and this is also common characteristic of neuropeptides derived from eleutherozoan L-type SALMFamide precursors. On the other hand, the presence of an alanine residue preceding the Pro-Phe-NH_2_ motif in the crinoid peptides is atypical because neuropeptides derived from eleutherozoan L-type SALMFamide precursors typically have an eponymous leucine residue in this position.

Discovery of putative L-type SALMFamide precursors in crinoids is important from an evolutionary perspective because it indicates that the origin of both L-type and F-type SALMFamide precursors can be traced back to the common ancestor of extant echinoderms. Furthermore, the discovery that both the F-type and L-type SALMFamide genes in *A. japonica* have phase 0 introns in the same locations provides new evidence that F-type and L-type SALMFamide precursors are paralogous and probably evolved by gene duplication in a common ancestor of extant echinoderms (Escudero Castelán et al., 2022). Accordingly, the presence or absence of an intron preceding the start codon in eleutherozoan F-type and L-type SALMFamide precursor genes, respectively, presumably reflects intron retention or loss in this lineage.

### Discovery of other predicted crinoid neuropeptide precursors

Our analysis of crinoid transcriptome/genome sequence data has also enabled the identification of several predicted crinoid neuropeptide precursors (PCNP1-20). For the majority of these proteins, we have not identified sequence similarities with neuropeptide precursors in other taxa that would provide a basis for ascribing orthology. Therefore, further studies on crinoids will be necessary to investigate if these proteins are indeed neuropeptide precursors. For example, mass spectrometry could be used to determine if the predicted structures of candidate novel crinoid neuropeptides are correct. Furthermore, mRNA *in situ* hybridization methods could be employed to determine if the PCNPs are expressed in neurons and *in vitro* pharmacological experiments could be performed to investigate physiological roles of these putative neuropeptides.

One exception to the above is AnjapPCNP1, which contains multiple copies of peptides with an N-terminal Ala-Asn (AN) motif—AN peptides. Precursors of AN peptides have been identified in other echinoderms (Rowe and Elphick, 2012; Semmens et al., 2016; Zandawala et al., 2017; Chen et al., 2019) and thus these appear to be a family of neuropeptide precursors in the phylum Echinodermata. Little is known about the expression of the AN peptides that have been identified in starfish, brittle stars and sea cucumbers (Semmens et al., 2016; Zandawala et al., 2017; Chen et al., 2019) but evidence that AN peptides are neuropeptides has been reported in studies on sea urchins. Quantitative analysis of gene expression during development of the sea urchin *S. purpuratus* revealed a massive increase in expression of the AN peptide precursor (SpANPP) gene from 48 to 70 h after fertilization, which coincides with the period when the larval nervous system is formed. Furthermore, analysis of *S. purpuratus* larvae using mRNA *in situ* hybridization and immunocytochemistry revealed the expression patterns of the SpANPP gene and a neuropeptide derived from SpANPP (SpANP2), respectively, in sub-populations of neurons (Perillo et al., 2018; Wood et al., 2018). Discovery of AN peptide precursors in crinoids, as reported here, provides a basis for developmental analysis of their expression using mRNA *in situ* hybridization and comparison with findings from *S. purpuratus* (Perillo et al., 2018; Wood et al., 2018).

### Mapping neuropeptide expression provides new insights into the molecular architecture of nervous systems in larval, post-metamorphic (pentacrinoid), and adult crinoids

Cloning of a cDNA encoding the *A. mediterranea* F-type SALMFamide precursor (Elphick et al., 2015) and use of this cDNA here to generate DIG-labeled anti-sense probes for transcripts encoding this precursor has enabled the first analysis of the expression of a neuropeptide precursor in three developmental stages of a crinoid species: doliolaria larvae, post-metamorphic pentacrinoids and adults. Furthermore, employing antibodies to the *A. rubens* neuropeptide ArCT we report here the first analysis of calcitonin-type neuropeptide expression in crinoids. Collectively, these anatomical studies have provided new insights into molecular architecture of crinoid nervous systems (Figure 14), as discussed below.

#### Nervous system of doliolaria larvae of *Antedon mediterranea*

The nervous system of crinoid doliolaria larvae consists of a basiepithelial nerve plexus with a higher concentration of neurons and fibers anteriorly, where serotonergic and GABAergic populations can be found at the level of the apical organ, located dorsally under the apical pit (Nakano et al., 2009; Mercurio et al., 2019). Here we provide the first description of neuropeptidergic cells in the doliolaria larvae, with F-type SALMFamide precursor expression revealed in two previously undescribed dorso-lateral groups of cells located between the second and third ciliary band (Figures 11A–C), and therefore clearly separate from the apical organ complex. Low level expression of the F-type SALMFamide precursor was also detected in cells located in an anterior-ventral position between the vestibule and the adhesive pit (Figure 11B). Interestingly, cells in this region have also been reported to express miR-7 (Mercurio et al., 2019) a microRNA that has a conserved role in neurosecretory cells (Tessmar-Raible et al., 2007). Thus, these findings provide new insights into the diversity of cell types in the nervous system of crinoid larvae.

#### Nervous system of pentacrinoids of *Antedon mediterranea*

During settlement and metamorphosis the nervous system associated with the apical organ of doliolaria larvae degenerates (Nakano et al., 2009), while the fate of the rest of the nervous system is unknown. In the post-metamorphic pentacrinoid stage the basiepidermal nerve plexus comprises scattered serotonergic and GABAergic neurons across the calyx but only GABAergic neurons in the stalk. Additionally, the tube feet contain a population of serotonergic neurons alongside GABAergic and cholinergic cells (Mercurio et al., 2019). The entoneural nervous system, which forms the major component of the adult nervous system, starts to develop as an aboral nerve center (ANC) located at the base of the calyx. From the ANC a thick stalk nerve, comprising cholinergic neurons, runs along the stalk and five brachial primordia, which have been previously hypothesized to develop into the brachial nerves, project toward the oral side of the animal (Mercurio et al., 2019).

Analysis of F-type SALMFamide precursor expression revealed stained cells scattered across different regions of pentacrinoid specimens (Figures 11D–H). These included cells associated with the ectoneural systems of the mouth and in the digestive system (Figure 11E), but no expression was detected in tube feet. Several stained cells were also revealed in the entoneural system, including three to five cells in the ANC (Figure 11H), two cells in the proximal region of stalk nerve (Figure 11F) and a variable number of cells beneath the epidermis in the aboral region of the calyx (Figure 11G), which we hypothesize to be part of the brachial nerve primordia.

Use of immunohistochemistry with antibodies to the *A. rubens* neuropeptide ArCT enabled visualization of extensive calcitonin-type neuropeptide immunoreactivity in the pentacrinoid nervous system (Figures 12A–Di). A few immunoreactive cell bodies were labeled in the ANC (Figures 12B,Bi), from which fibers project into the stalk. These do not represent the entire stalk nerve, but are grouped into five bundles (Figure 12Bi), revealing a higher level of regionalization in this portion of the entoneural system than previously described. Interestingly, immunoreactive fibers were also revealed in the brachial nerve primordia (Figure 12A). Fibers in the brachial nerve primordia have been labeled previously with antibodies against tubulin (Mercurio et al., 2019), but here we show for the first time presence of a peptidergic component to this part of the nervous system. Furthermore, immunostaining was also revealed in the circumoral nerve ring connected to the brachial nerve primordia on the oral side of the calyx, at the level of the radial plates (Figures 12C,Ci). This nerve ring is composed of groups of neurons located between the saccules and interconnected by fibers. The nerve ring also appears to be connected with single ArCT-like immunoreactive fibers that run within the tube feet (Figures 12D,Di), possibly indicative of a link between entoneural and ectoneural components of the nervous system. Interestingly, however, ArCT-like immunoreactive fibers were only observed in some of the tube feet. Overall, the immunohistochemical analysis with ArCT antibodies revealed a complex neuropeptidergic system that develops after metamorphosis and is concentrated in the entoneural system, which eventually develops into the most prominent component of the nervous system in adult crinoids.

#### Nervous system of adult *Antedon mediterranea*

The nervous system of adult crinoids comprises a conspicuous aboral entoneural component, with a bowl-shaped ANC in the calyx connected to a pentagonal ring commissure from which the brachial nerves enter each arm and run through the brachial nerve canal of the ossicles, sending projections to pinnules, flexor muscles, and ligaments of the arm (Hyman, 1955). The brachial nerve comprises both serotonergic and dopaminergic cells (Candia Carnevali et al., 1996; Sugni et al., 2010). Hyponeural and ectoneural components are also present: the first as a ring in the oral connective tissue of the calyx that sends slender pairs of cords on the oral side of each arm; the second as an ectodermal plexus concentrated orally at the base of the ambulacral groove epithelium of the arms, around the mouth and in the esophagus (Heinzeller and Welsch, 1994). The plexus at the base of the ambulacral groove of the arm contains dopaminergic neurons (Candia Carnevali et al., 1996; Sugni et al., 2010). Finally, a basiepithelial nerve plexus is also present in the digestive system.

Previous studies have investigated the distribution of SALMFamide-like immunoreactivity in two species of *Antedon*, using antibodies to the starfish SALMFamide neuropeptides S1 and S2. In *A. bifida*, S2-like immunoreactivity was detected in pinnular branches of the brachial nerve, with cell processes projecting orally between ossicles (Heinzeller and Welsch, 1994). In *A. mediterranea*, both S1-like and S2-like immunoreactivity were revealed in the medulla and cortex of the brachial nerve (Bonasoro et al., 1995). However, a limitation of these pioneering studies was that the specific identity of neuropeptides labeled with antibodies to the starfish peptides S1 and S2 could not be determined. With the discovery of transcripts encoding SALMFamide precursors in *A. mediterranea*, as reported previously (Elphick et al., 2015) and in this study, transcript-specific probes could be used to more precisely analyze SALMFamide expression in this species. Thus, here we used mRNA *in situ* hybridization methods to investigate the location of transcripts encoding the F-type SALMFamide precursor in sections of the arms of adult *A. mediterranea*. This revealed expression in cells distributed along the length of the brachial nerve, consistent with descriptions of the locations of neuronal cell bodies based on electron microscopic analyses (Heinzeller and Welsch, 1994), and in branches of the brachial nerve that innervate pinnules and interossicular muscles, but no expression was revealed in the ambulacral groove (Figures 11I–K). These findings are consistent with the data obtained from whole-mount mRNA *in situ* hybridization on pentacrinoid specimens, as seen above, where F-type SALMFamide precursor expressing cells were revealed in the ANC, in the brachial nerve and possibly in brachial nerve primordia, but not in the tube feet. Thus, collectively these findings indicate that F-type SALMFamide precursor expression is largely associated with the entoneural component of the nervous system of post-metamorphic crinoids, with apparently no evidence of expression associated with ectoneural and hyponeural systems of the ambulacra.

Immunohistochemical analysis of calcitonin-type neuropeptide expression in sections of adult *A. mediterranea* revealed a widespread pattern of immunostaining in the nervous system that was compatible with corresponding mRNA *in situ* hybridization. Thus, in the entoneural system, immunolabeled nerve fibers were revealed in the brachial nerve (Figure 12G) and, accordingly, *in situ* hybridization revealed segmentally repeated clusters of cells expressing the calcitonin-type precursor AmCTP (Figure 12I). This staining pattern suggests that the immunolabeled peptide is located in the neurites that extend from cell bodies containing AmCTP transcripts. This is consistent with transmission electron microscopic studies that have revealed neurites running longitudinally through the brachial nerve, with dense core vesicles characteristic of peptidergic neurons along their length (Heinzeller and Welsch, 1994; van den Pol, 2012). Immunostaining was also revealed at the base of the ectoneural component of the nervous system in the ambulacral groove (Figure 12H) and in accordance with this pattern labeled with antibodies to the starfish peptide ArCT, use of mRNA *in situ* hybridization revealed cells expressing the calcitonin-type precursor AmCTP located in the same position (Figure 12J). Transmission electron microscopy of the ectoneural plexus has shown that neuronal cell bodies are located at the base of the ambulacral groove and have neurites that branch extensively (Heinzeller and Welsch, 1994). Thus, staining patterns in which calcitonin precursor transcripts (Figure 12J) are confined to a few cells at the base of the ambulacral groove and ArCT-like immunoreactivity (Figure 12H) is more broadly distributed close to the location where cell bodies are expected to be is consistent with the known morphology of the nervous system in this anatomical structure. Interestingly, immunolabeling was also revealed in cells/fibers located aboro-laterally to the ectoneural region of ambulacral groove in a position consistent with the location of the hyponeural component of the nervous system (Figure 12H). Therefore, calcitonin-type neuropeptide expression may be associated with all three major compartments of the nervous system in adult *A. mediterranea*—the entoneural, ectoneural, and hyponeural systems. In the calyx, calcitonin-type neuropeptide expression was revealed in cells and processes located at the base of the mucosal epithelium in the esophagus, but not in other regions of the digestive system. This expression pattern suggests that the esophageal nervous system may be a continuation of the ectoneural plexus of the ambulacral groove that is distinct from the intestinal basiepithelial plexus, as has been proposed previously (Heinzeller and Welsch, 1994).

### The vasopressin/oxytocin-type neuropeptide crinotocin regulates arm mechanics in *Antedon mediterranea*

Analysis of the expression of the vasopressin/oxytocin-type neuropeptide (crinotocin) precursor in *A. mediterranea* using mRNA *in situ* hybridization methods revealed stained cells in the brachial nerve and in branches of the brachial nerve that innervate interossicular muscles and ligaments of the arm. Accordingly, we investigated the *in vitro* effects of crinotocin on arm preparations from *A. mediterranea*. This revealed that crinotocin causes a significant increase in the average rate of extension (i.e., upward bending) of arm preparations in comparison with preparations superfused with seawater alone (Figure 13D and Table 1). This effect of crinotocin was concentration-dependent (Figures 13E,F) and evidence of the specificity of the effect was provided by the observation that other neuropeptides tested, the starfish SALMFamide S1 and S2 and the starfish luqin-type peptide ArLQ, had no effect on the rate of extension (Table 1). Furthermore, the effect of crinotocin was blocked by the anesthetic procaine (Figure 13G). The mechanism by which crinotocin could exert its effect on arm preparations is discussed below.

Crinoids and other echinoderms exhibit the unusual characteristic of having mutable collagenous tissues (MCTs), which can rapidly change their mechanical properties (stiffening or destiffening) under neural control (Wilkie et al., 2021). The main interossicular ligaments in the arm of *A. mediterranea* consist of MCT and previous studies have revealed that the neurotransmitter L-glutamate triggers autotomy of arm preparations, this effect being attributed to a drastic loss of tensile strength of the ligaments at normally rigid syzygial joints (Wilkie et al., 2010; see Figure 1B). Crinotocin did not induce fracture at syzygial joints, and therefore appears not to be involved in the activation of autotomy, but it did cause an increase in the upward bending rate of arm preparations, with bending occurring at mobile diarthrial joints (Figure 1B). As explained by Wilkie et al. (2010), in the experimental set-up used in these experiments, an increase in the upward flexion rate of the arm in response to an externally applied agent can be due to destiffening of the diarthrial ligaments either alone or in combination with brachial muscle contraction. Our observation that the effect of crinotocin is blocked by the anesthetic procaine, a sodium channel blocker, provides evidence that the response is nervously mediated. The immediate effectors of ligament destiffening are likely to be the juxtaligamental cells whose perikarya are in functional contact with motor nerves (Welsch et al., 1995; Wilkie et al., 2010; Wilkie and Candia Carnevali, in press).

Although the experimental data obtained here do not allow us to determine whether the action of crinotocin on *A. mediterranea* arm preparations can be attributed to effects on the diarthrial ligaments alone or on both the ligaments and the brachial muscles, further insights into this issue could be obtained by generating specific antibodies to crinotocin for immunohistochemical mapping of its expression in *A. mediterranea* and investigation of its occurrence in branches of the brachial nerve that innervate specifically brachial ligaments or muscles.

Our discovery of the effect of crinotocin on arm preparations from *A. mediterranea* is of interest also from a comparative perspective with respect to actions of vasopressin/oxytocin-type neuropeptides in other taxa (Odekunle and Elphick, 2020). Both vasopressin and oxytocin are well-known for their excitatory effects on smooth muscle in mammals, with their names originating from their powerful contractile effects on the vasculature (vasopressin) and uterine muscles (oxytocin) (Odekunle et al., 2019). Interestingly, investigation of the effects of vasopressin/oxytocin-type neuropeptides in other echinoderms has revealed excitatory and inhibitory effects on neuromuscular preparations. Thus, the neuropeptide echinotocin causes contraction of both tube foot and esophagus preparations from the sea urchin *Echinus esculentus* (Elphick and Rowe, 2009). In contrast, the neuropeptide asterotocin causes relaxation of cardiac stomach and apical muscle preparations from the starfish *A. rubens* (Odekunle et al., 2019). In this context, it will be of interest to determine how crinotocin causes an increase in the upward bending rate of arm preparations from *A. mediterranea* because it may provide insights into the evolution of vasopressin/oxytocin-type neuropeptide function in the phylum Echinodermata. Accordingly, experimental studies on ophiuroids (brittle stars) and holothurians (sea cucumbers) are also needed to obtain a broader perspective on the actions of vasopressin/oxytocin-type neuropeptides in echinoderms, and this has been facilitated recently by determination of the sequences of vasopressin/oxytocin-type neuropeptides in species belonging to these echinoderm classes (Zandawala et al., 2017; Chen et al., 2019).

## General conclusion

In this study we present the first multi-gene and multi-technique analysis of neuropeptides in crinoid echinoderms. Our analysis of transcriptome/genome sequence data has yielded a rich resource of neuropeptide precursor sequences, which provide a strong basis for future studies. Focusing on selected neuropeptides as exemplars, we report here how developmental analysis of neuropeptide expression in crinoids using mRNA *in situ* hybridization and immunohistochemical methods can provide new insights into the molecular anatomy of crinoid nervous systems in larval, post-metamorphic (pentacrinoid) and adult animals (Figure 14). There now exists the exciting prospect to apply these experimental techniques to many other neuropeptide types to obtain comprehensive maps of peptidergic neurons in the nervous systems of crinoids. Furthermore, we report here for the first time the application of *in vitro* pharmacological methods to investigate the physiological roles of neuropeptides in crinoids, showing the effect of the vasopressin/oxytocin-type neuropeptide crinotocin on the mechanical behavior of arm preparations from *A. mediterranea*. The same bioassay could be employed to investigate the *in vitro* actions of other neuropeptides identified in this study. Also, as highlighted above with respect to relaxin-type peptides, it will be interesting to identify neuropeptides that trigger spawning in crinoids. Furthermore, bioassays will also need to be developed to investigate the effects of neuropeptides on crinoid larvae; for example, by monitoring effects on larval feeding and swimming behavior. Discovery of neuropeptide actions in crinoids, as a sister group to the eleutherozoans, will surely provide valuable insights into the evolution of neuropeptide function in the phylum Echinodermata and in the animal kingdom as a whole.

## Data availability statement

The datasets presented in this study can be found in online repositories. The names of the repository/repositories and accession number(s) can be found in the article/Supplementary material.

## Author contributions

AA, LY-G, TR, and MRE: analysis of crinoid sequence data and identification of neuropeptide precursors. DS: isolation of *A. mediterranea* RNA for transcriptome sequencing. RW, ZA, DI, JD, and WC: cloning and sequencing of cDNAs encoding crinoid neuropeptide precursors. AA, GG, RW, ZA, DI, EO, ME, CF, MS, FB, and MRE: analysis of neuropeptide expression in *A. mediterranea* using mRNA *in situ* hybridization and/or immunohistochemistry. AA, IW, MS, and FB: investigation of the *in vitro* pharmacological effects of crinotocin in *A. mediterranea*. AA, CF, MS, and FB: animal sampling and maintenance. AA, IW, LY-G, GG, and MRE: writing of the first draft of the manuscript. MRE: initiation and management of the project. All authors: feedback on and approval of final version of the manuscript.

## Footnotes

1
https://www.ncbi.nlm.nih.gov/sra/SRX3627697

2
https://www.ncbi.nlm.nih.gov/sra/SRX1480801

3
https://www.ebi.ac.uk/ena/browser/view/PRJNA305899?show=reads

4
http://transdecoder.github.io/

5
https://github.com/JekelyLab/Thiel_Yanez-Guerra_et_al_2021

6
http://ibs.biocuckoo.org/
