# Supplementary material for: Discovery and functional characterization of neuropeptides in crinoid echinoderms
Source: Front Neurosci. 2022 Dec 13;16:1006594. doi: 10.3389/fnins.2022.1006594 (PMC9793003; doi:10.3389/fnins.2022.1006594)
Supplement: Supplementary file 1 [file Data_Sheet_1.pdf]

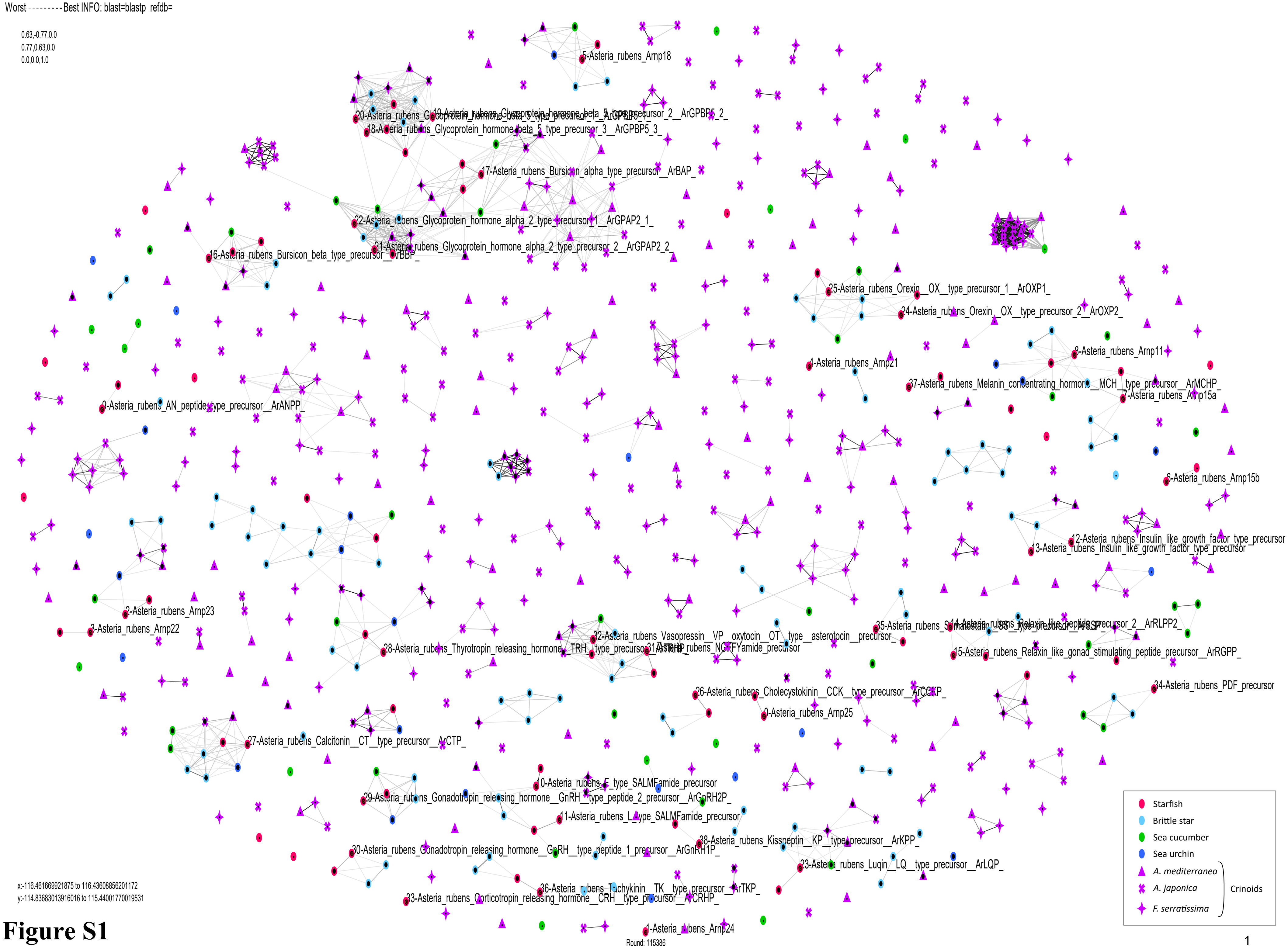
Worst Best INFO: blast=blastp refdb=
0.63,-0.77,0.0
0.77,0.63,0.0
0.0,0.0,1.0
5-Asteria_rubens_Arnp18
18-Asteria_rubens_Glycoprotein_hormone_beta_5_type_precursor_3__ArGPBP5_3_
17-Asteria_rubens_Bursicon_alpha_type_precursor__ArBAP_
22-Asteria_rubens_Glycoprotein_hormone_alpha_2_type_precursor_1__ArGPAP2_1_
21-Asteria_rubens_Glycoprotein_hormone_alpha_2_type_precursor_2__ArGPAP2_2_
16-Asteria_rubens_Bursicon_beta_type_precursor__ArBBP_
25-Asteria_rubens_Orexin__OX__type_precursor_1__ArOXP1_
24-Asteria_rubens_Orexin__OX__type_precursor_2__ArOXP2_
8-Asteria_rubens_Arnp11
4-Asteria_rubens_Arnp21
37-Asteria_rubens_Melanin_concentrating_hormone__MCH__type_precursor__ArMCHP_
7-Asteria_rubens_Arnp15a
9-Asteria_rubens_AN_peptide_type_precursor__ArANPP_
6-Asteria_rubens_Arnp15b
12-Asteria_rubens_Insulin_like_growth_factor_type_precursor
13-Asteria_rubens_Insulin_like_growth_factor_type_precursor
2-Asteria_rubens_Arnp23
3-Asteria_rubens_Arnp22
32-Asteria_rubens_Vasopressin__VP__oxytocin__OT__type__asterotocin__precursor_
28-Asteria_rubens_Thyrotropin_releasing_hormone__TRH__type_precursor
15-Asteria_rubens_Relaxin_like_gonad_stimulating_peptide_precursor__ArRGPP_
34-Asteria_rubens_PDF_precursor
26-Asteria_rubens_Cholecystokinin__CCK__type_precursor__ArCCKP_
0-Asteria_rubens_Arnp25
27-Asteria_rubens_Calcitonin__CT__type_precursor__ArCTP_
10-Asteria_rubens_F_type_SALMFamide_precursor
29-Asteria_rubens_Gonadotropin_releasing_hormone__GnRH__type_peptide_2_precursor__ArGnRH2P_
11-Asteria_rubens_L_type_SALMFamide_precursor
38-Asteria_rubens_Kisspeptin__KP__type_precursor__ArKPP_
30-Asteria_rubens_Gonadotropin_releasing_hormone__GnRH__type_peptide_1_precursor__ArGnRH1P_
23-Asteria_rubens_Luqin__LQ__type_precursor__ArLQP_
36-Asteria_rubens_Tachykinin__TK__type_precursor__ArTKP_
33-Asteria_rubens_Corticotropin_releasing_hormone__CRH__type_precursor__ArCRHP_
1-Asteria_rubens_Arnp24
x:-116.461669921875 to 116.43608856201172
y:-114.83683013916016 to 115.44001770019531
Round: 115386
Starfish
Brittle star
Sea cucumber
Sea urchin
A. mediterranea
A. japonica
F. serratissima
Crinoids

Figure S1

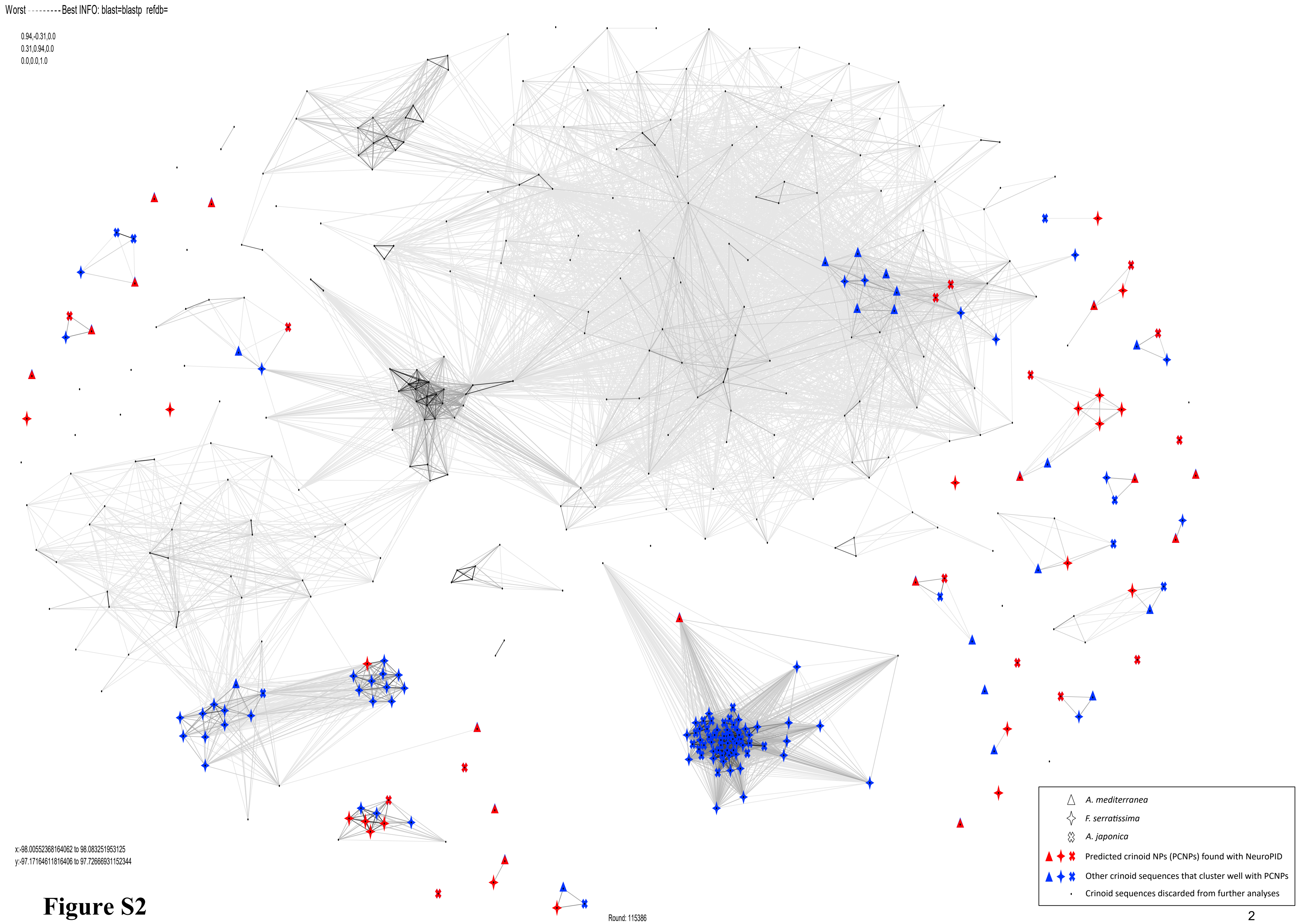
Worst ---------- Best INFO: blast=blastp refdb=
0.94,-0.31,0.0
0.31,0.94,0.0
0.0,0.0,1.0
x:-98.00552368164062 to 98.083251953125
y:-97.17164611816406 to 97.72666931152344
Round: 115386
A. mediterranea
F. serratissima
A. japonica
Predicted crinoid NPs (PCNPs) found with NeuroPID
Other crinoid sequences that cluster well with PCNPs
Crinoid sequences discarded from further analyses

Figure S2

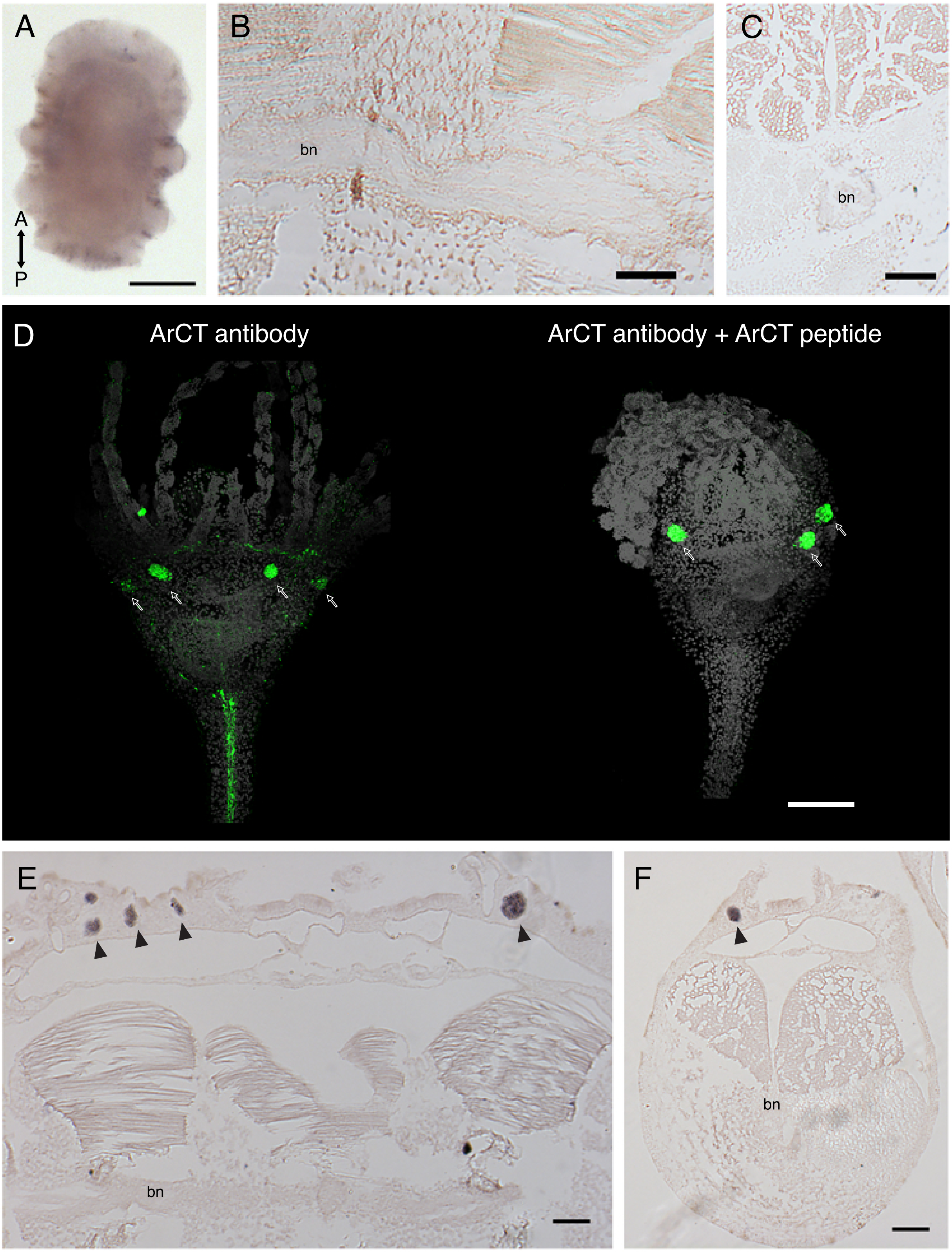
A
A
P
B
bn
C
bn
D
ArCT antibody
ArCT antibody + ArCT peptide
E
bn
F
bn

Figure S3

Table S1

Order	Family	Species	Source	Details	cd-hit	BUSCO (version 4.0.6)					
						Complete (tot)	Complete (single)	Complete (double)	Fragmented	Missing	BUSCO database used
Comatulida	Antedonidae	***Antedon mediterranea***	Elphick et al. 2015	Translated+predicted proteins with Transdecoder v5.5.0	none	**79.70%**	78.50%	1.20%	15.50%	4.80%	metazoa_odb10 (954)
					96%	**79.70%**	78.50%	1.20%	15.50%	4.80%	metazoa_odb10 (954)
		Florometra serratissima	https://www.ebi.ac.uk/ena/browser/view/PRJNA305899	Downloaded fastq reads; removed adaptors with Trimmomatic v0.36; assembled with Trinity v2.11.0; translated+predicted proteins with Transdecoder v5.5.0	none	**96.90%**	38.30%	58.60%	2.10%	1.00%	metazoa_odb10 (954)
					96%	**96.80%**	89.50%	7.30%	2.10%	1.10%	metazoa_odb10 (954)
	Comatulidae	***Anneissia japonica***	https://www.ncbi.nlm.nih.gov/assembly/GCF_011630105.1/	Downloaded protein FASTA file.	none	**97.00%**	70.00%	27.00%	1.40%	1.60%	metazoa_odb10 (954)
					96%	**97.10%**	93.60%	3.50%	1.40%	1.50%	metazoa_odb10 (954)

Table S2

ASW	S1	S2	CRI	LUQ
-0.01	-0.012	-0.012	0.008	0
-0.004	-0.002	0.006	0.034	-0.022
0.002	0.000	0.004	-0.008	-0.006
-0.006	-0.046	-0.002	0.034	-0.002
-0.002	0.032	0.004	0.064	0.002
-0.014	-0.008	0.004	-0.008	-0.008
-0.008	-0.016	-0.008	0.084	-0.01
0.002	-0.038	-0.048	0.058	-0.03
-0.024	-0.008	0.004	0.016	-0.014
-0.014	0.002	-0.092	-0.024	0.012
-0.048	-0.008	-0.002	-0.02	-0.068
-0.026	-0.032	0.000		0.014
0.014		-0.006		0.026

Anova: Single Factor

SUMMARY

Groups	*Count*	*Sum*	*Average*	*Variance*
ASW	13	-0.138	-0.010615385	0.000242256
S1	12	-0.136	-0.011333333	0.000424242
S2	13	-0.148	-0.011384615	0.000786256
CRI	11	0.238	0.021636364	0.001310255
LUQ	13	-0.106	-0.008153846	0.000546974

ANOVA

Source of Variation	*SS*	*df*	*MS*	*F*	*P-value*	*F crit*
Between Groups	0.00934849	4	0.00233712	3.6323319	0.010506146	2.53358327
Within Groups	0.036675058	57	0.00064342			
Total	0.046023548	61				

t-Test: Two-Sample Assuming Unequal Variances

	ASW	*S1*
Mean	-0.010615385	-0.011333333
Variance	0.000242256	0.000424242
Observations	13.000	12.000
Hypothesized Mean Difference	0.000	
df	20.000	
t Stat	0.09771072	
P(T<=t) one-tail	0.461567306	
t Critical one-tail	2.527977003	
P(T<=t) two-tail	0.923134612	
t Critical two-tail	2.84533971	

	ASW	*CRI*
Mean	-0.010615385	0.021636364
Variance	0.000242256	0.001310255
Observations	13	11
Hypothesized Mean Difference	0	
df	13	
t Stat	-2.747949736	
P(T<=t) one-tail	0.008301378	
t Critical one-tail	2.650308838	
P(T<=t) two-tail	0.016602757	
t Critical two-tail	3.012275839	

	ASW	*S2*
Mean	-0.010615385	-0.011384615
Variance	0.000242256	0.000786256
Observations	13	13
Hypothesized Mean Difference	0	
df	19	
t Stat	0.086481551	
P(T<=t) one-tail	0.465994256	
t Critical one-tail	2.539483191	
P(T<=t) two-tail	0.931988511	
t Critical two-tail	2.860934606	

	ASW	*LUQ*
Mean	-0.010615385	-0.008153846
Variance	0.000242256	0.000546974
Observations	13	13
Hypothesized Mean Difference	0	
df	21	
t Stat	-0.315919401	
P(T<=t) one-tail	0.377591061	
t Critical one-tail	2.517648016	
P(T<=t) two-tail	0.755182122	
t Critical two-tail	2.831359558	

Table S3

CRI	CRI+PRO	ASW
-0.004	0.002	-0.018
0.002	0.002	0.000
0.094	-0.010	0.002
0.012	0.002	-0.010
-0.002	0.002	0.002
0.000	-0.008	-0.008
0.050	-0.008	0.016
0.052	-0.010	0.004
-0.008	0.004	0.008
0.076	0.004	0.002
0.108	0.042	0.064
-0.022	0.046	-0.036
-0.010	0.002	-0.018
0.046	0.000	-0.008
-0.014	0.000	0.002
0.000	0.008	0.006
0.000	-0.010	-0.020
0.006	0.000	-0.018
-0.016	-0.004	-0.056
-0.030	-0.008	0.012
-0.006	0.002	-0.024
0.000	0.002	-0.016
0.044	0.002	-0.008
0.022	0.002	0.004
0.140	0.030	0.002
0.012	-0.008	-0.008
-0.008	0.010	0.036
0.044	0.004	0.004
0.068	0.000	0.022
0.010	0.000	-0.008
0.002	0.012	0.016
0.012	0.000	0.004
0.030	-0.010	0.014
0.050		0.010
0.008		0.010
0.048		
-0.012		

Anova: Single Factor

SUMMARY

Groups	*Count*	*Sum*	*Average*	*Variance*
CRI	37	0.804	0.02173	0.001481
CRI+PRO	33	0.102	0.003091	0.000173
ASW	35	-0.016	-0.00046	0.000416

ANOVA

Source of Variation	*SS*	*df*	*MS*	*F*	*P-value*	*F crit*
Between Groups	0.01025	2	0.00512	7.16033	0.00123	3.08547
Within Groups	0.073	102	0.00072			
Total	0.08325	104				

t-Test: Two-Sample Assuming Unequal Variances

	CRI	*ASW*
Mean	0.02173	-0.00046
Variance	0.001481	0.000416
Observations	37	35
Hypothesized Mean Difference	0	
df	55	
t Stat	3.078995	
P(T<=t) one-tail	0.001619	
t Critical one-tail	2.174331	
P(T<=t) two-tail	0.003238	
t Critical two-tail	2.461509	

	CRI+PRO	*ASW*
Mean	0.003091	-0.00046
Variance	0.000173	0.000416
Observations	33	35
Hypothesized Mean Difference	0	
df	58	
t Stat	0.857126	
P(T<=t) one-tail	0.197452	
t Critical one-tail	2.171459	
P(T<=t) two-tail	0.394903	
t Critical two-tail	2.457532	

	CRI	*CRI+PRO*
Mean	0.02173	0.003091
Variance	0.001481	0.000173
Observations	37	33
Hypothesized Mean Difference	0	
df	45	
t Stat	2.770503	
P(T<=t) one-tail	0.004053	
t Critical one-tail	2.186752	
P(T<=t) two-tail	0.008106	
t Critical two-tail	2.478733	

Figure S1. Cluster analysis of sequences (CLANS) of crinoid neuropeptide precursors and neuropeptide precursors from other echinoderms that belong to known families. CLANS analysis facilitated identification of crinoid members of known neuropeptide families. Red = sequences from starfish species (*Asterias rubens* and *Acanthaster planci*); Light blue = sequences from brittle star species (*Amphiura filiformis* and *Ophionotus victoriae*); Green = sea cucumber sequences (*Apostichopus japonicus*); Dark blue = sea urchin sequences (*Strongylocentrotus purpuratus*). Sequences from *Asterias rubens* have the name highlighted; Pink = crinoid sequences. The pink triangles are *A. mediterranea* sequences; the pink crosses are *A. japonica* sequences; the pink stars are *F. serratissima* sequences. Clustering and connections are shown at p-value = $1.00E^{-12}$. However, clustering performed better at different p-values for different neuropeptide families; consequently, identification of crinoid sequences was based on CLANS at different p-values specific to the neuropeptide family.

Figure S2. Cluster analysis of sequences (CLANS) of other predicted crinoid neuropeptide precursors (PCNPs). The sequences identified from NeuroPID analysis were BLASTed against sequence data from the other crinoid species and then clustered using CLANS. Sequences identified as putative neuropeptide precursors from the NeuroPID pipeline are in red. In blue are the sequences obtained from the BLAST analysis that cluster well with the putative neuropeptide precursors and that were therefore collected for manual examination. Sequences not highlighted did not cluster well and were discarded from further examination. Triangles = *A. mediterranea*; Crosses = *A. japonica*; Stars = *F. serratissima*. Clustering and connections are shown at p-value = $1.00E^{-8}$. However, clustering performed better at different p-values for different proteins; consequently, the choice of sequences to keep for further analysis was based on CLANS at different p-values specific to each cluster containing the originally identified putative neuropeptide precursor.

Figure S3. Negative controls for experiments investigating neuropeptide expression in *A. mediterranea*. (**A-C**) *in situ* hybridization with sense probes for the F-type SALMFamide precursor in doliolaria specimens (**A**) and adult arm sections (**B-C)** showing absence of staining. (**D**) Comparison of fluorescence observed in pentacrinoids with the ArCT antiserum and the ArCT antiserum pre-absorbed ArCT antigen peptide. Fluorescence observed in the nervous system with the ArCT antiserum is abolished by pre-absorption of the antiserum with the antigen peptide, demonstrating the specificity of this immunostaining. Fluorescence in saccules (arrows) is also present in specimens incubated with the pre-absorbed ArCT antiserum and therefore this is non-specific fluorescence. (**E-F**) *in situ* hybridization with sense probes for the crinotocin precursor in adult sections, showing absence of staining in neural structures. Non-specific staining is present in saccules (arrowheads). Scale bars: 100 μm. Abbreviations: A: anterior side; bn: brachial nerve; P: posterior side.

Table S1. Crinoid species proteomes used in this study with respective BUSCO values as a measure of proteome completeness. Sources and details of processing, where applicable, are indicated for the proteomes of the three crinoid species analysed in this study. For all three species a cd-hit with identity threshold of 96% was carried out to remove almost identical transcript variants. Then BUSCO was performed on both the non-cd-hit and the cd-hit versions of the proteomes. In general, proteome completeness, as measured by the percentage of total and single-copy BUSCO genes present in the proteome, was good for all three species. The cd-hit improved the level of single-copy BUSCO for *F. serratissima* and *A. japonica*. Cd-hit versions of the proteomes were used for subsequent analyses.

Table S2. Statistics for the comparison of neuropeptide effects on *Antedon mediterranea* arm preparations. First an ANOVA Single Factor was performed, and this highlighted that there is a statistically significant difference between some of the treatments. Then, multiple t-tests with a Bonferroni-corrected alpha value were applied to the single treatments versus between some of the treatments. Then, t-tests with Bonferroni-corrected alpha value were applied to the single treatments versus control (ASW). 106 arm preparations from three animals were used for this experiment, although one data point for crinotocin + procaine hydrochloride was excluded as an outlier (see Supplementary File 5). While crinotocin has a significant effect versus ASW ($p = 0.00324$), the crinotocin + procraine hydrochloride is not significantly different from the negative control ($p = 0.39490$).

Table S3. Statistics for the anaesthetisation experiment. First an ANOVA Single Factor was performed, and this highlighted that there was a statistically significant difference between some of the treatments. Then, t-tests with Bonferroni-corrected alpha value were applied to the single treatments versus control (ASW). 106 arm preparations from three animals were used for this experiment, although one data point for crinotocin + procaine hydrochloride was excluded as an outlier (see Supplementary File 6). While crinotocin has a significant effect versus ASW ($p = 0.00324$), the crinotocin + procraine hydrochloride is not significantly different from the negative control ($p = 0.39490$).
