# Supplementary material for: Discovery and functional characterization of neuropeptides in crinoid echinoderms
Source: Front Neurosci. 2022 Dec 13;16:1006594. doi: 10.3389/fnins.2022.1006594 (PMC9793003; doi:10.3389/fnins.2022.1006594)
Supplement: Supplementary file 2 [file Data_Sheet_2.DOCX]

**Monomeric Neuropeptides**

**Calcitonin-type precursors**

**>Amed Calcitonin-type precursor**

**MNTSWISSVLISCLLICIVQSASASRERRGIITSNFLRDLADRIDIYNEWVELNDLDNHNQLQQEKRGCDVFGGCAQLKVGRELAIDTLQKGSGGMFGSSGPGRKRRSTQSNDVNV**

**>Anjap Calcitonin-type precursor**

**MNSSWIASILLPCLLICIVQSASINRERRGVISSDFLRELADRIDFYNEWLQLNEFDNNNKQVQEKRGDCDSFGGCAQLTASQQLAYRTRHRDLSNLFGTGGPGRKRRSTQPSDAQN**

**Eclosion hormone-type precursors**

**>Amed Eclosion hormone-type precursor (partial)**

**MSISKVMLFTTLFYTCLQIADMLPTNEDFSRENAMFVRLLNGIPTPDSNDLNPAERGLGLYENSKRAADVCPRECFACSAVLQGIRPIICIRGCQGSHLVSPS...**

**Kisspeptin-type precursors 1**

**>Amed Kisspeptin-type precursor 1**

**MQLVILCLALLWPCTVLTDETPTALRNPDLENDIFQQGFDRALFLHNLREKHVLAYIIKAAAEDLLANEALQMKPYEGNDSPSESNVAVTEDDMEYETTRSSDGTDVSKRGNCPGDRCLRIKKKPKQTSSCSHNACLRILPFGKRALGSRGVQFKRPSSGSKTRSTSGGGGGGRTNLRPSFPFGKKDYE**

**>Fser Kisspeptin-type precursor 1**

**MQLVVLCLAIFWPWTVLSDEIPSALRNTDIENDFFQDGFDRRLFPHNLKQKQMLAYIIKAAAEDLLANEALQIADNNSPSESNVAVAEEGTDYGTTGSSDGTDVSKRGGNCLGDRCVRIKKKPKQTSACSHNACLRILPFGKRALGPPRLSKQGGGFKRPAGGKTRSTSGGGGGGGGGGRRNLRPSFPFGKKNYE**

**>Anjap Kisspeptin-type precursor 1**

**MQLIVLCLAIIWPWTVLSDEVPSALRSTEIENDFLRGGFDRRILSHTLKQKHVLAHIIKSAAEDLLANEALQMKTYEESDWPSDSNVVTEDGIDYAATGSEETEVSKRGNCPGDRCLRIKKKPKQTSACSHNACLRILPFGKRASPRSSSQGWQKRGSGKTRTAGGGGRTDLRPSFAFGKKDF**

**Kisspeptin-type precursors 2**

**>Amed Kisspeptin-type precursor 2**

**MIRSAVDVILFITLSSILVSSRALPLQHGRVTPEKSYEGFLDEPEVSEDSLYRNNILSDYGWRKKAVLMTDLKRDQSHEGKDKEDKRNQRKHSFRGMIGKRFFDQQLPSQAESKMCVMEVVIGFLFGEDQSINEPEVKHVWTRCKDNNRLLEN**

**>Fser Kisspeptin-type precursor 2 (partial)**

**MIRFVVDVTLFIILSSILVTSRASPLQHDRVTPEKSYEGFLDEAEESEDILYRNNILNDYGWRKQRVLMSDLK...**

**>Anjap Kisspeptin-type precursor 2**

**MIRFVVEVSLCITLSSILVTSRASPLQQGRVTPEKSFEGFLDEPASDDSVYLNNRLRDYGWGQQGVLMTDLKRDQQHGEKRDEEEDKRSHRIAHSFQGMLGKRFFDQQRPLQEEKPKCAMEVVLSFLLGADKTINDPEVKHVLTRCKQNASNRLFDN**

**Luqin-type precursors**

**>Amed Luqin-type precursor 1**

**MVNNSPVTSILCMTLVYVLILSTVTPIEAVRRLNHPPKHSRWGKRTYTSLSESNKDMADIKEDLLNISTTLRTLFNKDNTDFIDTPGLCDGPFLKRLKCIIRSRHTHDITSQTHFIDLSNQEV**

**>Amed Luqin-type precursor 2**

**MGISKIYIGLLVVLILQSILTSKVSSNPVRKLNEPPSLSRWGKRAMKISEEEPQELTIKMFGDENAFCVHIAAGGIYRCVTTKDERYNNYRK**

**>Fser Luqin-type precursor 1**

**MAISKIHIGLLVVLVLQSILTSTVSSNPVRKLKDPPSLSRWGKRAMTVSDDEPQELTLQLFGDENVFCVKIAEGGIYRCISGLGIFDQYRK**

**>Anjap Luqin-type precursor 1**

**MANSALTSVICLVLVQFILLSLVTPLDAARRVKHPPNMSRWGKRLYTLPADSHEEIIGTNGQSLTTTSNPLFNEASSLKTPEECGGPFLKKLRCIIRSRHAQGMTSQHQSIEVSNQDSVNALLVLF**

**Melanin-concentrating hormone-type precursor**

**>Amed Melanin-concentrating hormone-type precursor**

**MQKLVLLCLVIMIVAASAVFAAAAADDWSLPPKFDDRPLTDATAAEATKDNVGSHTSTILKRSRNSSVRLVPCMDWLRKRWRYCWVRSKSS**

**>Fser Melanin-concentrating hormone-type precursor**

**MHKLILLLLVVMTIVSTGLAEHWSLKSDGTPLTDATSIVSTELAEHWSLKYDDATSAKDNILQASTIHKRSRGSSLRLVRCMDWIRKRWRYCWVNGKSN**

**Orexin-type precursor**

**>Amed Orexin-type precursor**

**MQRRLWITLLLILAMFVDSTQCGRRKCKCPDARSGDCRIPPGCRCPLVRALCRSKPKSTLVGKRNASNSCSPGDPLVLERPPPRWSSSFCSL**

**Pedal peptide/orcokinin-type precursor**

**>Fser Pedal peptide/orcokinin-type precursor**

**MLYFKSFGRMWLVVWLLNAALITTAISEQVDNVRAEVLSNAEIADEEAKELIDNLMHSKKDFSSNDDELFQLNEEDKRGLFPTGGMDPLGASYFTGKRGADSNEENTDKRGFPNAGLDTLGSKYYNGKRAYPSSGGLDTLGSRYFTGKRSYPGSGGMDMLGNGYFNGKRAYPSSGGMDTLGNRYFNGKRSYPSSGGMDTLGSRYYSGKRAYPSSGGMDTLGNRYYNGKRMYPSSGGMDTLGSGYFNGKRAYPVSGGMDTLGSGYFNGKRAYPSSGGMDTLGSSFFNGKRGFPNAGLDTLGSRYYNGKRAYPSSGGMDTLGSRYYNGKRAIFDDFNTADSLHGFKKGSSFLHGGLSSDRMLGFKKRSVSSDEMKSE**

**>Anjap Pedal peptide/orcokinin-type precursor**

**MFYYKLFSRLWLVVCLLHAELITSADCEPTDHSRDQGISNAEIADVEAKELIDNLIKSKKEYSSDDDELFQFNEEDKRGLFPTGGMDPLGASYFTGKRGVDENEEEPDKRGFPNGRLDTLGSRYFNGKRAFPNTGGLDTLGNRYFNGKRGLYPSSGNMDTLGSGYFTGKRGFPSSGGIDTLGSRYFNGKRMFPSSGGMDTLGNGYFTGKRGFPSSGGIDTLGSGYFSGKRMFPSSGGMDTLGNGYFTGKRGFPSSGGIDTLGSGYFSGKRMFPSSGGMDTLGNGYFTGKRAFPSSGNLDTLGNRYFNGKRGFPSSGRIDTLGSSYFTGKRSIFDQYNTDSLHGFKKGSSFLHGGLSSGMDNHIPGFKKRALPSEDMKSE**

**Prolactin-releasing hormone/short NPF-type precursor**

**>Amed Prolactin-releasing hormone/short neuropeptide-F-type precursor**

**MGQTRCCVVGLLLFALMVSLLITRSSADTKEERELSDKERVAIGSILSGQRRPWRYGRRSWNPRPDQENSLYNAKVADLLRQQATEQNEYDNYIKSLISNNIPLKPIEWDEE**

**>Fser Prolactin-releasing hormone/short neuropeptide-F-type precursor**

**MRQTRCCLVLLFAFTVCVLITRSSADGKEERELSDKERVAIGSILSGQRRPWRYGRRSWNPRTDQEYSLYNAKVADLLRQRAAEQNEYDNYLKSLVSNNMPLKPMEWDED**

**F-type SALMFamide precursor**

**>Amed F-type SALMFamide precursor**

**MFSQPPLYLLLTWFLFQHSLLAQGHTGDNIREGGVRYNRPHGGGVPSKKANTSSEPINNWIRALPVLHRGLYFGKRVPANGYQLEDQFRDPAVAHLASKRNPALSEFMLGKRDPSFSSYMLGKRNPRLSDLMLGKRDPRLSDLMLGKRDPRLSDLMLGKRDPRLSDLMLGKRDPGFSDFTFGKRDALGDFMMGKREARLSDYIMGKRDPRISDFIMGRRELGENDVQRHMGNNYYDNKVEHEGKHYVLSDGNRERIEDNMNNVIYDDTDIPNQAEVSELQELESSSSVKRKAKFQRPVYPGNGKTPSQIWDTFGAGKRMSSVPDYEDEEENVQTETKRSADPKTSVRRFPPAALHKGLYFGKRAATWADM**

**>Fser F-type SALMFamide precursor**

**MFSQSPLFLLLTWFLFQHSLLTLGHTGDNIRDGGVRYNRPIGMPSKKANDTAPINNWIRALPLLHQGLYFGKRVQANPRYNEFQDDGDKELRDSTIGHLASKRNPAFSDFMLGKRDPRFSSFMLGKRDPRLSDLMLGKRDPRLSDLMLGKRDPRLGDFMLGKRDPRFSEFMLGKRDPRFSEFMLGKRDPRFSEFMLGKRDPALSDFMMGKREARLSDFIMGKRDPRLSEFMMGRRELGFGDHDTGRHTGNWKNYYDNEVEHQSNRYVLNDENRGRLEDNMGNVIYDDTDIPNQAEAGSEMQDLESSSSLKRKVKLHRPSYTGAGKTPSVLWNNLGAGKRMSSVQDYEDEDENVQTEIKRSADPAPKSSVRRFPPAALHKGLYFGKRAATWADM**

**>Anjap F-type SALMFamide precursor**

**MLRQPPFFLLLTWFLFQQCLLTLCYAGDNIHDAYNRPIGFPSKKANATAPIKNWIRALPLLHQGLYFGKRVIADEFNDFQDNDEKELRYSTLGHITSKRNPRLTSFMMGKRDPRFSDFMMGKRDPRFSDFMMGKRDPRFSDFIMGKRDPRFSDFMMGKRDPRFSDFMMGKRDSRLSDFMLGKRDPRFSDFIMGKRDPRLSEFMLGKRDARITDFMLGKRDARLSDFMMGRRELGFDEDENGRQTGHNYFQNEVDHQSDRYVLHGGDLETLEGSRGNVVYDDTDIPNQAEAAEFQELESSSSLKRGSGKGKLHRPQFTGTGKTPSQLWSNLGSGKRSSSVDYEDEDENVITEIKRSADPNPKTSVRRFPPAALHKGLYFGKRAANWADI**

**L-type SALMFamide precursor**

**>Amed L-type SALMFamide precursor**

**MRKESILTVILAMVVCSEFVSAESADRRGYGLVNPNGRFGFASGTLGQNGFYRSGSLSAKRELYPIGDEEIVREIRKNPTRGLRFHKGAPFGKRAYDLPTFEENADNTV**

**>Fser L-type SALMFamide precursor**

**MRKESICTVFLAMVLCSEIINAESAERRGYGMINPNGRFGFASGTLGQNGFYRSGTLGSKRELGYPIGDDEVVREVRKNPTRGLRFHKGAPFGKRAYDLSNFDVQNAENTV**

**>Anjap L-type SALMFamide precursor**

**MHKECVFAVILAMVLCSEFVNAASDDYRRGSYNGMLNPNARFGFASGTLGQNGFYRSGSLSAKRDPYELGDEADEIVREVRRNPSKGLRFHKGAPFGKRAYDLEFKDDNTKGM**

**Sulfakinin/cholecystokinin-type precursor**

**>Amed Sulfakinin/cholecystokinin-type precursor**

**MVKAEFTILVVVSIGLISNNVFSSPLPKVHTRKQFDIISELKHNIEMAVVATIHQARDAQNSGTMDVAVIKDQIVGPKQIRDPGLDDYGMGMMFGKRSPENAWVDLLDDDHL**

**Vasopressin/oxytocin-type (crinotocin) precursor**

**>Amed Vasopressin/oxytocin-type (crinotocin) precursor**

**MQECCRILLVLFALVSLSTACFWRTCPVGGKRTAFRPLRQCPKCGPFEGGQCVGPLLCCGESFGCHLATPDTLVCSEESRLSTPCTGLELQSSCRSVEGGSCATSYICCNEDTCAIDYSCESGRISNSHLPQETSANRQLWDAFVNANN**

**>Fser Vasopressin/oxytocin-type (crinotocin) precursor**

**MQDCCRILLFLFALVSISTACFWRTCPMGGKRTAFRPIRQCPKCGPFEGGQCVGPLLCCGESFGCHLATPDTLVCSEESRLSTPCTGIELQSSCRSVEGGSCATSYICCNEDTCAIDYSCEAGMALGRISNSHLAQETSANRQLWDAFVNTNN**

**>Anjap Vasopressin/oxytocin-type (crinotocin) precursor**

**MQEGCRILLVLFALFAVSTACYWRKCPIGGKRTSSFRPLRQCPKCGPFEGGQCVGPLICCGESFGCHIATADTLVCSEESRLSTPCTGPELQPSCRAVEGGSCATSYICCNEDICAIDYSCEESETGMVRRTNSHLVNI**

**Heterodimeric Neuropeptides**

**Bursicon alpha-type precursors**

**>Amed Bursicon alpha-type precursor 1**

**MSSHPAFSTLMFLVSGVTIVSTICVPVGTIHTISVPGCRPKSVPSTGCRGMCLSYTRVSSTNYLELERSCKCCKADKFIVASVKLSCPNLDSPNLRVPLKKAESCSCRPCNSITVEDVATPDF**

**>Amed Bursicon alpha-type precursor 2**

**MVDSRILFILVIQILSVYSGRSNVGCSRQQIRLSISMADCRPRNVYPFGCRGQCASYTRVSPANFLEIDRQCKCCQVGEQVDLQVRLDCPKLKPPVGMVTVKSAKNCSCRPCNSVEVQSIEPFA**

**>Fser Bursicon alpha-type precursor 1**

**MHLSSSLLLCLVSCTLLLTSLVDAGKGRRKAKNVLSMLDDSEPTVVATATRRNRENRRNGEENRRNGDENRRNGEENRRNGEGSHRGKSPDELLTPSSEEAIEITEKRYLRGDWCKTQPVKQIIEEEGCISRTITNRFCYGQCNSFYVPKTVKDREQDAFVSCAFCKPYKYKIITVTLRCPGRTPNTRRKKIKKIKQCRCIAIDVPVD**

**>Fser Bursicon alpha-type precursor 2**

**MVPIRIFLIMAIPILGIYSGRGSNIGCSKQQIRLTISMGECRPRVIYPFGCRGQCASYTRVSPANFLEIDRQCKCCQVGEQVDLQVRLDCPKLRPPVGTVTIKSAKNCSCRPCSSVEVQAVEPFAYKSP**

**>Anjap Bursicon alpha-type precursor 1**

**MNLSSSLLVCIVSCALLLLSSLTEAGKGRRKTKNVLSMLDDSEPTVMDNVPRRNRENRRNGEGSHRGKSPDELLTPSSEEAIEITEKQYLRGDWCKTQPVKQLIEEDGCISRTITNRFCYGQCNSFYVPKTVKDREQDAFVSCAFCKPYKYKMITVTLRCPGRNPNTKRKKIKKIKQCRCIAIDVPVD**

**Bursicon beta-type precursors**

**>Amed Bursicon beta-type precursor 1 (partial)**

**FACICDGATRGGGRRGVGGGGGGRTSDVVYFKCALSEISYPTQVKRFYTNPTSVNSKIRCEGTVLVKACEGNCPSSEMWSIKRGLTRVCRCCDISSKESINVMLRTCRDHTGRMLRGVARSAKVSV**

**>Amed Bursicon beta-type precursor 2 (partial)**

**MSYMHSIGLILVYAICFVLSKGASQSRRHPQCQAVTTVIKISQEVYEQDTKRTMFCRGSANVTSCEGTCKSRMIPKVSSPLGFTK**

**>Fser Bursicon beta-type precursor 2**

**MSYLYSLSLIFVYAICFVFARGVTQSRRHQQCQSVTTVIKIAQEVYEQDTRRTMFCRGNATVTSCEGTCKSRMIPKVSSPLGFSKVCKCCRETRLRIKTVELRQCYTENNILIPDEKYSVDIRVPESCVCSTCG**

**Glycoprotein hormone alpha-2-type precursors**

**>Amed Glycoprotein hormone alpha-2-type precursor 1**

**MLSSLTITVNQKMCLLLKSLVLITAIMLANGDNYWEQPGCHVVGYTKEVRIPGCHVEYVQMNACRGYCMSYSFLSSQDTLQRSGGLQVFSSYGSCCTIGQTHDVNIVLQCVDNQVYRDTFRSAKTCECSLCDVSD**

**>Amed Glycoprotein hormone alpha-2-type precursor 2**

**MYVHFCKKHSFLQLLYLVVVLFLCVDSMGLKGKPGCARYYYAEKVSHPKKNCHPKMVLLTRCNGYCAKSSSAEPVISFRSVVRHPFKYNCQSCQDLTSSMRAIRLQCANNERVYATYRYILECSCKSCRIDRRSKS**

**>Fser Glycoprotein hormone alpha-2-type precursor 1**

**MLSSLTITVNHKMYLLLKSLVLIAAVMLANGDNYWEQPGCHVVGYTKEVRIPGCHVEYVQMNACRGYCMSYSFLSSQDTLQRSGGLQVFSSYGSCCTIGQTHDVNIVLQCVDNQVYKDTFRSARTCECSLCDVSD**

**>Anjap Glycoprotein hormone alpha-2-type precursor 1**

**MLSSLTILVNQKMCLLLKTFVLVAAITLVNGNYWEQPGCHVVGYTKEVRIPGCHVEYVQMNACRGYCMSYSFLSSQDTLQRSGGLQVFSSYGSCCTIGQTHDVNIVLQCVDNQVYRDTFRSARTCECSLCDVSD**

**Glycoprotein hormone beta-5-type precursors**

**>Amed Glycoprotein hormone beta-5-type precursor 1**

**MRMDHRRLYAFLAVLPFVLISTVNSVDPSTTTDCFKHTKMKHIASKPGCQDQVIYVHGCWGRCDSNEIPELEPPYTAAFHPMCYYDTYTTATKTLSNCANGVDPTYTYINAVTCVCKAPSSSESAYAYRPDNYV**

**>Amed Glycoprotein hormone beta-5-type precursor 2**

**MFTIKMIHTWAIMATFLVFMEPGNAQRMEKFLQIVSDLASKPVVKYCRPIKMRTVVEREGCSPLTVLETTKCIGRCLSYEEPYIEAPYVKSSHQLCQLTIREVPLKKNYQMKCGKGVKTNYTHEDKFECRCGQPRSIRTSYKYRDAELMR**

**>Amed Glycoprotein hormone beta-5-type precursor 3**

**MQLWRVIHWLYLLPLVFLVSTLIATSSQGQALGCSILKNTQYEAHKHGCRTQIIRTDTCGGRCHTHQVPRLERPFIDSDHKLCSYSRVEWKSIQLNDCDPGVNRTYSFMNAKACQCRYCNSAYTNCLGV**

**>Fser Glycoprotein hormone beta-5-type precursor 1**

**MRMDRTSCAFLVVLTLLTISTVQSVDPSTTTDCFKHTKMKHIATKSGCEDQIIYVHGCWGRCDSNEIPELEPPYTAAFHPLCYYDTYATVTTRLSNCAPGVDPTYTYINAVTCVCKAPSSSESAYAYRPDTYV**

**>Anjap Glycoprotein hormone beta-5-type precursor 2**

**MIKIKIFTTCTMMMLLFSFMEPVNSQRMDKFLQIVGNLAAKPVVRYCRPVKMRKVIERQGCKPLTVLDTTKCVGRCLSYEEPHIEAPYVKSSHQLCQLTARDVPLKQQYQMKCDQGVNPYYTHEDSFECRCSQPRSLRTSYRYRDAELS**

**Insulin-type precursors**

**>Amed Insulin-type precursor**

**MEVCRRRLCLLMWTWLLAVLVGSTYSLQDYSDRSHHDWARVWTVESMRQCHEDLREMVHISCHNDPRKITSKRSIFIPRNEATGFLSRFLRTRRPSELHEDCCLDSRGCTWEEVAEIACINNRRRMHRPGSPVGR**

**>Fser Insulin-type precursor**

**MEVCRRRTWLVTWTWLMAALVGSTYSLQDYSDRSHYDWARVWTVESMRQCHEDLREMVHISCHNDPRKITSKRSIFIPRNEATGFLSRFLRTRRPSELHEDCCLDSRGCTWEEVAEIACINNRRRIHRPGSPVGR**

**Insulin-type precursors**

**>Amed Insulin/bombyxin-type precursor**

**MTKLFDGQHEHVATTSLVITIVVVLLSQVQVTDARDWYCGSAANTLMNFCQSCYATKRSHSSLPLLKSKSDEMFLTKERASGYLEAKRSRLFSSVNLDRRQHGQTNFVTECCYNPCTRFEMIKYCCTSRQIEFNDTEGKK**

**>Fser Insulin/bombyxin-type precursor**

**MTKLFCGQHKNMATASLVITAIVVLLSHVQVTEARDWYCGNAATTLMDICQSCYATKRGYSSLPSLKSRTDGMFLTKERASGYLETKRSRLFSSVNLDRRQHGQTNFVMECCYNPCSRFEMIKYCCTSRQLEFNDTEGKK**

**Relaxin-type precursors**

**>Anjap Relaxin-type precursor**

**MKDQGRNRFMVVLLISFVLCDQILCTTIRCGSEFRAAVRTVCMAKRMPSYGPNVYENALGLLAGGRLPYDSEQEIKTRLFPRLRKSRSTYTGPHDFCCTHGCDDDFIRVRVC**

**Predicted Crinoid Neuropeptides**

**Predicted crinoid neuropeptide precursor 1 (PCNP1)**

**>Amed Predicted crinoid neuropeptide precursor 1 (PCNP1)**

**MYVTFALILILVPNGILSAESVIGTEPEKVLVKPVNTKCDNEKATDTTTTKSSGCVVSGDTKDSDEKRGNYFQRLQKDTKEISDDKEDDKRANYHRRLRSDGDDVEKEPEKRGNFHRRLRMDETDMAANKRANDFDESEMTEEEK**

**>Fser Predicted crinoid neuropeptide precursor 1 (PCNP1)**

**MFVAFALILILVPNGILSAESVIATAEKVLTNIAPEDNKCNDKAGKGDGTYVASPDTPTGSPPGCATSGDEKDSATVDKRANFFQRLRKNAEDISEDSVDEVKRANYFRRPRADETDLVADKKANYHRRPRAEEMDMTEDKRANYYRRPRADESDMTEEKKSAPIIIAANYRPRADESYMTEEEKRANYHRRPRADESDMTEEEKRANYHRRPRADDSYMTEEEKRANYHRRPRADESDMTEEEKRANYHRRPRADESDMTEEEKRANYFRRPRADEMYMTEEEKRANYFRRPRADEMYMTEEEKRANYYRRPRADESDMTEEEKRAN**

**>Anjap Predicted crinoid neuropeptide precursor 1 (PCNP1)**

**MFVAFALILIIAPNEIFSAENVIATAENGLKNIKPDVKCEDKQFAANPTGTTVGTPAGCVVSGDSNDATSIDKRGNYFQRPRKDAEDINEKRGNYYLHPRSDEADEKRANYFQRPRKDETGMALDDKRANWHYYRPPRADETDDTEEKRANYWRQHPRKRDSEEEKRANYFMRPRKDEDYLSEDKRANYWRQPRADETDETEEKRANYWRQHPRKRDSEEEKRANYFMRPRKDEDDISEDKRANYWRQPRADETDETDEKRANYWRQRPRKRDSEEEKRANYFMRPRKDESDFSEDKRANYWRQPRADESDETEEKRANYWRQRPRKRDSEEEKRANYFMRPRKDESDIYEDKRANYWRQPRADETDESEEKRANYWRQRPRKRDSEEEKRANYFMRPRKDVKETDDAEGN**

**Predicted crinoid neuropeptide precursor 2 (PCNP2)**

**>Amed Predicted crinoid neuropeptide precursor 2 (PCNP2)**

**MYREIFTCLVIAAVLASSTQAQKRRIHKGTQWGKRTSLFDLNDDRNLSAFPDSSKQIDLGNLVRAWINFEMQRQENSKTFPTFDQALGYETSNFEKEIK**

**>Fser Predicted crinoid neuropeptide precursor 2 (PCNP2)**

**MYREILTCLVIAAVLTSTHAQKRRIHKGTQWGKRTSLYDLNDERNPSGYPEPSKQMDLGKLVRAWINFEMQRQGNTKTFQTFDQALGYETSNFEKEMK**

**Predicted crinoid neuropeptide precursor 3 (PCNP3)**

**>Amed Predicted crinoid neuropeptide precursor 3a (PCNP3a)**

**MDVRITVLCVLLALVFVGVQAQPPPGYCSGITGNRPPECTSLIGKKSLLNALLQRLLERQEEEEEKEEKEERNAQVNERHYHAQDTREYDIEQEQEIRLLDFLL**

**>Amed Predicted crinoid neuropeptide precursor 3b (PCNP3b)**

**MDFSIIVLCVGLLALVFVGVEAPPPPGWCGGIAGHRRPGCTSQWGKKSTLDALIEQLMARKEVLEEDEKEAAAAKDRDVVNERKGLNEPNYQDTREYDVEQEKEIRLLDFLL**

**>Fser Predicted crinoid neuropeptide precursor 3 (PCNP3)**

**MDLRITVLFVLALVFLCIEAQLPPGCNTGVNGKRPPGCGSQVGRKDLLNSLLERLLERELQLDETAQEERTVEKKAMEEYKVPETRRFEGEEREETVRLLDLLLDLEGEHGIKK**

**>Anjap Predicted crinoid neuropeptide precursor 3 (PCNP3)**

**MHLRFMVLLLSVLVLITCTEAPPPPECMSPVAQNRPSHCQVVWGRKALNTLERLLKKELIDEEMNTEERNLGDMEEAEGQETRNLDDENQNLYDEVRFLELLLDLKGENNIKK**

**Predicted crinoid neuropeptide precursor 4 (PCNP4)**

**>Amed Predicted crinoid neuropeptide precursor 4 (PCNP4)**

**MFYFKLFGSIWLVGCLLNVALIPTVSSEQAARADVLSNVEIADEEAKELIDNLIKSKKDFSSNDDELFQLNDEEKRGLFPTGGMDPLGASYFTGKRAADSTEDSSEKIGFNDKRAFPGSGGMD**

**Predicted crinoid neuropeptide precursor 5 (PCNP5)**

**>Amed Predicted crinoid neuropeptide precursor 5 (PCNP5)**

**MASIAKAVSCLVAMTVLLALLHVESVSARRGHPKSNFILMPGRRSPFDDLLQQENTRRDNILESATPDLLSEEDDSTKIDLELLREIEKWLKAQTSSKTYKKPFMQDAGENGEY**

**>Fser Predicted crinoid neuropeptide precursor 5 (PCNP5)**

**MASTAKAVSCLVAMTVLLALLHMDSVSARRGHPKSQFILMPGRRSPFDLLQENQRREDILESATPDLLADEDDSTQIDLELLREIEKWLKAQSSSKTYMKPFMQEAGDNGEY**

**Predicted crinoid neuropeptide precursor 6 (PCNP6)**

**>Anjap Predicted crinoid neuropeptide precursor 6 (PCNP6)**

**MNLTTTAVFTLMLASCCLCDLQVNRKRQPGCGCTTRICMYNCVSGKRTMDMQELYEKKNALPFLREFSNEQDESGPFKNRFYQYLTAKRGNAYSEVGVKSDY**

**Predicted crinoid neuropeptide precursor 7 (PCNP7)**

**>Amed Predicted crinoid neuropeptide precursor 7 (PCNP7)**

**MEVRHLTYVVIVILGISTLSTVAYAGCANICIEHRLSGSQCAKLCGKPLGKRSEISGLMENQEKIDETASSSIQSALVQHFNKLRPELQRIVLELIVDLEIKEQIEG**

**>Fser Predicted crinoid neuropeptide precursor 7 (PCNP7)**

**MEVRHITCVIMVLIGISSLTSTVSSTSACTRFCIDNNYSGRECAKTCGRLFGKREPALTENIGWINDRDDEFANKPSESMQSTLVEHFNQLRPEMQRIVFELILDLELSSEMEG**

**>Anjap Predicted crinoid neuropeptide precursor 7 (PCNP7)**

**MEVRHIMYLLIVVIGLSSFKSAASATCSDRCNKRRLTGDACAKLCGRIYGKRQTTLSENSRWMDKIEEVASEVPEEIEESSLLQHFYQLRPQLQRIVYQVILELEISQMEG**

**Predicted crinoid neuropeptide precursor 8 (PCNP8)**

**>Fser Predicted crinoid neuropeptide precursor 8 (PCNP8)**

**MRQTRCCLVLLFAFTVCVLITRSSADGKEERELSDKERVAIGSILSGQRRPWRYGRRSWNPRTDQEYSLYNAKVADLLRQRAAEQNEYDNYLKSLVSNNMPLKPMEWDED**

**Predicted crinoid neuropeptide precursor 9 (PCNP9)**

**>Amed Predicted crinoid neuropeptide precursor 9 (PCNP9) (partial)**

**...VVVLGVVFLASSVTCCKRAAITTSEDKPSLETENTQIKPENMPNEWPSLVGKKGEEAQKFISKERPELKIVILNKDDMMTMDFREDRVRIFVDDNQVVVRPPKTG**

**>Fser Predicted crinoid neuropeptide precursor 9 (PCNP9)**

**MQMKSVLLVVVGVVFFASSVTCCKRSAPSASGGENVEESEKQNIEDNKMETQWPALVGKKGEEAEKLILKARPDLKIFILPQDAMMTMDFREDRVRILVDENQIVVRPPKVG**

**>Anjap Predicted crinoid neuropeptide precursor 9 (PCNP9) (partial)**

**...MCIWCPSLHRSIIACAVGTAVFVYFWSIIASKQDIPLDKKAEEAKKFILEESPELQIYILPEDSMMTMDYRTDRVRIFIDENQIVVKPPKVG**

**Predicted crinoid neuropeptide precursor 10 (PCNP10)**

**>Amed Predicted crinoid neuropeptide precursor 10 (PCNP10)**

**MYSILFTVMVTITMVGCGSTEDIAEYESGPIQNTLGLGIPQIWPEEGDIAELEEQIAKHEAIIKYLNHLRNQYLQGSQSKRSGFFNRRSGKNAYDVDWQKIGDYAAKRSGFFNRKRNAYEERK**

**>Fser Predicted crinoid neuropeptide precursor 10 (PCNP10)**

**MYSILFTVMIAITMVGVGSTDEMGEYETGQIQNTIGLGIPQIWPEEGDIAELDEQIAKHEAVIKYLNHLRNQYLQGSQTKRSGFFNRRSGNSMYDVDWHKIGDYAAKRSGFFNRKRSGYDERK**

**>Anjap Predicted crinoid neuropeptide precursor 10 (PCNP10)**

**MYRPTILFALMISAMMAGVGNTEDIGEFEAGQIQNTVGLGIPKIWPDEGDIAELAEEQIAQHEEIINYLNHLRNEYLQGSQTKRSGFFNRRSGSPMYDVPSQDWNKRGDFTSAKRSGFFNRKRSGYEQRK**

**Predicted crinoid neuropeptide precursor 11 (PCNP11)**

**>Amed Predicted crinoid neuropeptide precursor 11 (PCNP11)**

**MDITMTTRNTLKIITFLLIGCCCLTQSVPVLKFSGQISLEGDEESGLALPWILMSKMIDSDRPMNIDTDNSSEYYSGEQNDVGDIAKRGNGRSDLMRYLYSGKIGKAASTMGRQPGRR**

**>Fser Predicted crinoid neuropeptide precursor 11 (PCNP11)**

**MDITMTTSNTLKIITFLLIGCLCLTESVPVLKFSGQITLETDEGVALPWMLMSKMIDSDRLMNTDIDNSSEYYSGEQNDVADVTKRGNNRSDLLRYLYSGKLNKAASTMARKQTGRR**

**>Anjap Predicted crinoid neuropeptide precursor 11 (PCNP11)**

**MDTMTTSNTLKIITVLLLGCICLTESVPVLRFSGQISLETDGESGAVLPWVLMSKMLESDRFMNTDLDGQSEYYSGEENNLDVTKRGSNRETAIKQQLFGGRLRKVAGINLGRKPGGR**

**Predicted crinoid neuropeptide precursor 12 (PCNP12)**

**>Amed Predicted crinoid neuropeptide precursor 12 (PCNP12)**

**MVSPMSMLNIGAALFLCMCLQQCFLTVSSEYDNPDTNTFEQDTEPENAAWRLSNGALRNKRPWLGGRKRDSTAYFIKREPIRMGGYRENLDVKRAFQEWLSEQRRNFEDETPYEQEYYQYKRPMMNGKRNMINGGV**

**>Fser Predicted crinoid neuropeptide precursor 12 (PCNP12)**

**MVSSMSMLNIGAALFLFMCLQQCFSTVSSEYDSPDTNTFDQDTEQENAAWLTHGGLINKRPWLGGKRSSTGFFIKRDPIRMGGYREMDVKRAFQEWLTEQQRRSYEETPYQQEYYEYKRPMMNGKRNMINGGV**

**>Anjap Predicted crinoid neuropeptide precursor 12 (PCNP12)**

**MVSSMSMMNMGAALFLFMCLQQCFLTVSSEYDAPDTNTFDQDTEQEKAAWLTHGGLINKRPWLGGKRSSTSYFTKRDPIRMGGYREMEAKRAFEEWLSEQRRNYEEIPFEQEYYELRKRPMMNGKRNMVNGGP**

**Predicted crinoid neuropeptide precursor 13 (PCNP13)**

**>Amed Predicted crinoid neuropeptide precursor 13 (PCNP13)**

**MEYKRMVTVCVVLVLCVCVSSERADNKTPEDKTLHKEGMSIVQKVYRILETITNMERNQEREAERKKEQDRYTPKRKGGEFKTQGWRKKRSTFGHNLMDITFIQQLDSDVNELKLDVKDFLEDLGKVMFYLIFNTVKCILKLR**

**>Fser Predicted crinoid neuropeptide precursor 13 (PCNP13)**

**MGYKDLFIILIVCVFFVCANSQRSDNQTPEEVSEAETIHEEGMRIVQRIYSILETITNMQRNQEMEEKRSEEQAIYDAKRRRGGDFKSQAWRKKRSTFGKNLMDITFVQQLDSETKDLKTDVIDFLEDLRQFRELFFQWL**

**>Anjap Predicted crinoid neuropeptide precursor 13 (PCNP13)**

**MGFKVLCILSLAGFFVFVSSQNVETNRTPEDVSKSETVREEGMRIVQRIYSILDTIKNMERNKKNEERRKTEITEYNPKRRPGGDFSTQAWRKKRSTIGKNLMEITFVQRLDSETMELRIEVKEFLQTIGQWLRKALNKSHIDVGDTGTRGFFAELASNEEVFVE**

**Predicted crinoid neuropeptide precursor 14 (PCNP14)**

**>Anjap Predicted crinoid neuropeptide precursor 14 (PCNP14)**

**MNRVAVIILAVGLFVSLIEAKPYYKLFKKSGPNGGKRGPHGGNAVGSNSKSAEDASGDKSGQLGGNAGGRSSSEDDGIGNESPSSWSWSTWWNNLKSSYPASWSSWWNNLESSYPASWSSWWNNLGNVNKKAISIVARGKKVKKATAKKSLLMKALAHNKALARKRLVA**

**Predicted crinoid neuropeptide precursor 15 (PCNP15)**

**>Amed Predicted crinoid neuropeptide precursor 15 (PCNP15)**

**MILLKLLFVLLIIGIISNEARGWSIGRHKRQSGDEIQISYNLNPILHDKGRRRRLHGGDFTAQNDSSEALSKNSMEKIDNKRRVSSDVVIDVVINKDWLNEPVVKDSDRMGMLDKDRFLDEIAL**

**Predicted crinoid neuropeptide precursor 16 (PCNP16)**

**>Amed Predicted crinoid neuropeptide precursor 16 (PCNP16)**

**MKYIMVLILVVAIVNMSNGKPTKKGHRLNGKANPVHGKRGSYTNGENQCIQGHPNFWACNDGDCIQKNKLCDGKFDCRFGEDERGPNVCQIKNSR**

**Predicted crinoid neuropeptide precursor 17 (PCNP17)**

**>Amed Predicted crinoid neuropeptide precursor 17 (PCNP17)**

**MASVITTAVLVSVLTFVLQIVSSIPVEINGSGDVDIQLLGEITTSQHTFQRGKRAMKVSGSQSCSSHNQCNRGQCCAFSFGRKICKDSQKTRKLGETCSIFDVHKYLDLNDLTRSFTTCPLVCRKGLRCRATQGKSRKRIAVCSRR**

**>Anjap Predicted crinoid neuropeptide precursor 17 (PCNP17)**

**MASFIATTAVFISVMTFVLQLVSSIPIEVNGGGDGEEYTQLFGEITTHDTFQRGKRAMKVSSSRSCSSNADCRRDQCCAFDFGRKVCKASQRVRNLGETCSFVDIHKYLDLHDLTRSFTRCPMICKEGLRCWRTHERYSKSNTRKSVCRR**

**Predicted crinoid neuropeptide precursor 18 (PCNP18)**

**>Anjap Predicted crinoid neuropeptide precursor 18 (PCNP18)**

**MFRCCVLTLMFVGLTVVQPDEDISCKDRLCGGIVNRDCITMCSSIGRKRSGTTYSYTCLHECIANGVPMNECKCGFAGRKRSANLDSLKDRSIGNFKTIIAGQGQSK**

**Predicted crinoid neuropeptide precursor 19 (PCNP19)**

**>Amed Predicted crinoid neuropeptide precursor 19 (PCNP19)**

**MVMRLSIVTLFVVMLLTPCFARPNGCPPQGCRIRSRQRWGKRDNTNSFATEKRLESAPSWIGNLSKNKNEARKVSPWMLTVFEHNNLKKTAYTE**

**Predicted crinoid neuropeptide precursor 20 (PCNP20)**

**>Fser Predicted crinoid neuropeptide precursor 20 (PCNP20)**

**MKLVLLVVVVCASMTDALFFGGKRPSGGGPGMGKRPNGGGGPDIGERPEKGDVDIDFLCERKDSCPVGDSNPVCGSDGITYDSPCELLKVKCDGVNSDLTPDHAGECGSEPLDRPTGRPTFGEQGRTRPTGRPNNIGGQRPTGRPISAGKDRLAGPISAGKDQMAGPISADNDQMAGPISADNDQMAGPISAD**
