# Supplementary material for: Discovery and functional characterization of neuropeptides in crinoid echinoderms
Source: Front Neurosci. 2022 Dec 13;16:1006594. doi: 10.3389/fnins.2022.1006594 (PMC9793003; doi:10.3389/fnins.2022.1006594)
Supplement: Supplementary file 4 [file Data_Sheet_4.DOCX]

**Predicted Crinoid Neuropeptide 1**

**Amed_PCNP_1 1 MYVTFALILILVPNGILSAESVIGTEPEKVL--VKPVNTKCDNE---------KATD-TTTTKSSGCVVSGDTKDSD--EKRGNYFQRLQKDTKEISD--
Fser_PCNP_1 1 MFVAFALILILVPNGILSAESVIAT-AEKVLTNIAPEDNKCNDKAGKGDGTYVASPD-TPTGSPPGCATSGDEKDSATVDKRANFFQRLRKNAEDISE--
Anjap_PCNP_1 1 MFVAFALILIIAPNEIFSAENVIAT-AENGLKNIKP-DVKCEDK------QFAANPTGTTVGTPAGCVVSGDSNDATSIDKRGNYFQRPRKDAEDINEKR
consensus *.*.******..**.*.***.**.* .*..* ...* . **... . . .. *..... **..***..*. ...**.*.***..*....*..

Amed_PCNP_1 85 ---------DKEDD-KRANYHRRLRSDGDDVEKE--------------------------------------PEKRGNFHRRLRMDETDMAANKRANDF-
Fser_PCNP_1 97 ---------DSVDEVKRANYFRRPRADETDLVAD----------------------------------------KKANYHRRPRAEEMDMTEDKRANYYR
Anjap_PCNP_1 93 GNYYLHPRSDEADE-KRANYFQRPRKDETGMALDDKRANWHYYRPPRADETDDTEEKRANYWRQHPRKRDSEEEKRANYFMRPRKDEDYLSEDKRANYWR
consensus * *. *****..*.* *.... . .*..*...*.* .* .....****...

Amed_PCNP_1 136 ----DESEMTEEEK--------------------------------------------------------------------------------------
Fser_PCNP_1 148 RPRADESDMTEEKKSAPIIIAANYRPRADESYMTEEEKRANYHRRPRADESDMTEEEKRANYHRRPRADDSYMT-----------------EEEKRANYH
Anjap_PCNP_1 192 QPRADETDETEEKR-------ANYWRQHPRKRDSEEEKRANYFMRPRKDEDDIS-EDKRANYWRQPRADETDETDEKRANYWRQRPRKRDSEEEKRANYF
consensus ...**...***.. ... ......... ... .. ... ....... . ...... . ........

Amed_PCNP_1 ----------------------------------------------------------------------------------------------------
Fser_PCNP_1 231 RRPRADESDMTEEEKRANYHRRPRADESDMTEEEKRANYFR-RPRADEMYMTEEEKRANYFRRPRADEMYMTEEEKRANYYRRPRADESDMTEEEKRAN-
Anjap_PCNP_1 284 MRPRKDESDFS-EDKRANYWRQPRADESDET-EEKRANYWRQRPRKRD---SEEEKRANYFMRPRKDESDIY-EDKRANYWRQPRADETDES-EEKRANY
consensus ... .... . ....... . ....... . ......... ... . .......... ... .. . ......... ....... . ......

Amed_PCNP_1 -----------------------------------
Fser_PCNP_1 -----------------------------------
Anjap_PCNP_1 377 WRQRPRKRDSEEEKRANYFMRPRKDVKETDDAEGN
consensus**

**Predicted Crinoid Neuropeptide 2**

**Amed_PCNP_2 1 MYREIFTCLVIAAVLASSTQAQKRRIHKGTQWGKRTSLFDLNDDRNLSAFPDSSKQIDLGNLVRAWINFEMQRQENSKTFPTFDQALGYETSNFEKEIK
Fser_PCNP_2 1 MYREILTCLVIAAVL-TSTHAQKRRIHKGTQWGKRTSLYDLNDERNPSGYPEPSKQMDLGKLVRAWINFEMQRQGNTKTFQTFDQALGYETSNFEKEMK
consensus ***** *********. ** ****************** **** ** * * *** *** ************* * *** **************** ***

**Predicted Crinoid Neuropeptide 3**

**Amed_PCNP_3a 1 MDVRITVLCV-LLALVFVGVQAQPPPGYCSGITGNRPPECTSLIGKKSLLNALLQRLLERQE--EEEEKEEKEERNAQ-------VNERHYHAQDTREYD
Amed_PCNP_3b 1 MDFSIIVLCVGLLALVFVGVEAPPPPGWCGGIAGHRRPGCTSQWGKKSTLDALIEQLMARKEVLEEDEKEAAAAKDRDVVNERKGLNEPNY--QDTREYD
Fser_PCNP_3 1 MDLRITVLFV--LALVFLCIEAQLPPGCNTGVNGKRPPGCGSQVGRKDLLNSLLERLLERELQLDETAQEERTV-------EKKAMEEYKV--PETRRFE
Anjap_PCNP_3 1 MHLRFMVLLLSVLVLI-TCTEAPPPPECMSPVAQNRPSHCQVVWGRKAL--NTLERLLKKELIDEEMNTEERNLGD---------MEEAEG--QETRNLD
consensus *.....**.. .*.*... ..* .**..... ...*...*....* *.........*.... ...*...*.. ... ..... * .. . **...

Amed_PCNP_3a 91 IEQEQ---E-IRLLDFLL----------
Amed_PCNP_3b 99 VEQEK---E-IRLLDFLL----------
Fser_PCNP_3 90 GEERE---ETVRLLDLLLDLEGEHGIKK
Anjap_PCNP_3 87 DENQNLYDE-VRFLELLLDLKGENNIKK
consensus .*... * *.*. **.. .. ...**

**Predicted Crinoid Neuropeptide 5**

**Amed_PCNP_5 1 MASIAKAVSCLVAMTVLLALLHVESVSARRGHPKSNFILMPGRRSPFDDLLQQENTRRDNILESATPDLLSEEDDSTKIDLELLREIEKWLKAQTSSKTY
Fser_PCNP_5 1 MASTAKAVSCLVAMTVLLALLHMDSVSARRGHPKSQFILMPGRRSPFD--LLQENQRREDILESATPDLLADEDDSTQIDLELLREIEKWLKAQSSSKTY
consensus *** ****************** *********** ************..* *** ** ********** ***** **************** *****

Amed_PCNP_5 101 KKPFMQDAGENGEY
Fser_PCNP_5 99 MKPFMQEAGDNGEY
consensus ***** ** ******

**Predicted Crinoid Neuropeptide 7**

**Amed_PCNP_7 1 MEVRHLTYVVIVILGISTLSTVAYAG--CANICIEHRLSGSQCAKLCGKPLGKR----SEISGLMEN-QEKIDETASSSIQSALVQHFNKLRPELQRIVL
Fser_PCNP_7 1 MEVRHITCVIMVLIGISSLTSTVSSTSACTRFCIDNNYSGRECAKTCGRLFGKREPALTENIGWINDRDDEFANKPSESMQSTLVEHFNQLRPEMQRIVF
Anjap_PCNP_7 1 MEVRHIMYLLIVVIGLSSFKSAASAT--CSDRCNKRRLTGDACAKLCGRIYGKRQTTLSENSRWMDKIEEVASEVPEEIEESSLLQHFYQLRPQLQRIVY
consensus *****......*..*.*.... .... *. *......* ***.**...*** ..*...... .. . ......*.*..**..***..****.

Amed_PCNP_7 94 ELIVDLEIKEQIEG
Fser_PCNP_7 101 ELILDLELSSEMEG
Anjap_PCNP_7 99 QVILELEI-SQMEG
consensus ..*..**. ...****

**Predicted Crinoid Neuropeptide 9**

**Amed_PCNP_9 1 -------------VVVLGVVFLAS--SVTCCKRAAITTSEDKPSLETENTQIKPENMPNEWPSLVGKKGEEAQKFISKERPELKIVILNKDDMMTMDFRE
Fser_PCNP_9 1 MQM------KSVLLVVVGVVFFAS--SVTCCKRSAPSASGGENVEESEKQNIEDNKMETQWPALVGKKGEEAEKLILKARPDLKIFILPQDAMMTMDFRE
Anjap_PCNP_9 1 MCIWCPSLHRSIIACAVGTAVFVYFWSIIASKQDIP----------------------------LDKKAEEAKKFILEESPELQIYILPEDSMMTMDYRT
consensus . . ........*...... *....*. ... . ... .. . .. ...**.*** *.*....*.*.*.**. * *****.*.

Amed_PCNP_9 86 DRVRIFVDDNQVVVRPPKTG
Fser_PCNP_9 93 DRVRILVDENQIVVRPPKVG
Anjap_PCNP_9 73 DRVRIFIDENQIVVKPPKVG
consensus *****..*.**.**.***.***

**Predicted Crinoid Neuropeptide 10**

**Amed_PCNP_10 1 MY--SILFTVMVTITMVGCGSTEDIAEYESGPIQNTLGLGIPQIWPEEGDIAEL-EEQIAKHEAIIKYLNHLRNQYLQGSQSKRSGFFNRRSGKNAYDV-
Fser_PCNP_10 1 MY--SILFTVMIAITMVGVGSTDEMGEYETGQIQNTIGLGIPQIWPEEGDIAEL-DEQIAKHEAVIKYLNHLRNQYLQGSQTKRSGFFNRRSGNSMYDV-
Anjap_PCNP_10 1 MYRPTILFALMISAMMAGVGNTEDIGEFEAGQIQNTVGLGIPKIWPDEGDIAELAEEQIAQHEEIINYLNHLRNEYLQGSQTKRSGFFNRRSGSPMYDVP
consensus ** .***..*....*.*.*.*....*.*.*.****.*****.***.******* .****.**..*.*******.******.*********** .***

Amed_PCNP_10 97 --DWQKIGDY-AAKRSGFFNRKRNAYEERK
Fser_PCNP_10 97 --DWHKIGDY-AAKRSGFFNRKRSGYDERK
Anjap_PCNP_10 101 SQDWNKRGDFTSAKRSGFFNRKRSGYEQRK
consensus **.*.**. .***********..*..****

**Predicted Crinoid Neuropeptide 11**

**Amed_PCNP_11 1 MDITMTTRNTLKIITFLLIGCCCLTQSVPVLKFSGQISLEGDEESGLALPWILMSKMIDSDRPMNIDTDNSSEYYSGEQNDVGDIAKRGNGRSDLMR-YL
Fser_PCNP_11 1 MDITMTTSNTLKIITFLLIGCLCLTESVPVLKFSGQITLETDE--GVALPWMLMSKMIDSDRLMNTDIDNSSEYYSGEQNDVADVTKRGNNRSDLLR-YL
Anjap_PCNP_11 1 MD-TMTTSNTLKIITVLLLGCICLTESVPVLRFSGQISLETDGESGAVLPWVLMSKMLESDRFMNTDLDGQSEYYSGEENNL-DVTKRGSNRETAIKQQL
consensus **.****.*******.**.**.***.*****.*****.**.*...*..***.*****..*** **.*.*..*******.*...*..***..*..... .*

Amed_PCNP_11 100 YSGKIGKAAS-TMGR-QPGRR
Fser_PCNP_11 98 YSGKLNKAAS-TMARKQTGRR
Anjap_PCNP_11 99 FGGRLRKVAGINLGR-KPGGR
consensus ..*.. *.*. ...* ..*.***

**Predicted Crinoid Neuropeptide 12**

**Amed_PCNP_12 1 MVSPMSMLNIGAALFLCMCLQQCFLTVSSEYDNPDTNTFEQDTEPENAAWRLSNGALRNKRPWLGGRKRDSTAYFIKREPIRMGGYRENLDVKRAFQEWL
Fser_PCNP_12 1 MVSSMSMLNIGAALFLFMCLQQCFSTVSSEYDSPDTNTFDQDTEQENAAW-LTHGGLINKRPWLGG-KRSSTGFFIKRDPIRMGGYRE-MDVKRAFQEWL
Anjap_PCNP_12 1 MVSSMSMMNMGAALFLFMCLQQCFLTVSSEYDAPDTNTFDQDTEQEKAAW-LTHGGLINKRPWLGG-KRSSTSYFTKRDPIRMGGYRE-MEAKRAFEEWL
consensus ***.***.*.******.*******.******* ******.****.*.*** *..*.*.******** **.**..*.**.********* ...****.***

Amed_PCNP_12 101 SE-QRRNFEDETPYEQEYYQY-KRPMMNGKRNMINGGV
Fser_PCNP_12 98 TEQQRRSYE-ETPYQQEYYEY-KRPMMNGKRNMINGGV
Anjap_PCNP_12 98 SE-QRRNYE-EIPFEQEYYELRKRPMMNGKRNMVNGGP
consensus .* ***..* *.*..****.. ***********.***.**

**Predicted Crinoid Neuropeptide 13**

**Amed_PCNP_13 1 MEYKRMVTVCVVLVLCVCVSSERADNKTPED----KTLHKEGMSIVQKVYRILETITNMERNQEREAERKKEQDRYTPKRK-GGEFKTQGWRKKRSTFGH
Fser_PCNP_13 1 MGYKDLFIILIVCVFFVCANSQRSDNQTPEEVSEAETIHEEGMRIVQRIYSILETITNMQRNQEMEEKRSEEQAIYDAKRRRGGDFKSQAWRKKRSTFGK
Anjap_PCNP_13 1 MGFKVLCILSLAGFFVFVSSQNVETNRTPEDVSKSETVREEGMRIVQRIYSILDTIKNMERNKKNEERRKTEITEYNPKRRPGGDFSTQAWRKKRSTIGK
consensus *..* . .. .. .. .. .... .*.***... .*...***.***..*.**.**.**.**.. *..*. *. * .**. **.*..*.*******.*.

Amed_PCNP_13 96 NLMDITFIQQLDSDVNELKLDVKDFLEDLGKVM------------------FYLIFNTVKCILKLR
Fser_PCNP_13 101 NLMDITFVQQLDSETKDLKTDVIDFLEDLRQFREL----------------FFQWL----------
Anjap_PCNP_13 101 NLMEITFVQRLDSETMELRIEVKEFLQTIGQWLRKALNKSHIDVGDTGTRGFFAELASNEEVF-VE
consensus ***.***.*.***.. .*...*..**....... *. . . . .**

**Predicted Crinoid Neuropeptide 17**

**Amed_PCNP_17 1 MASVI-TTAVLVSVLTFVLQIVSSIPVEINGSGDVD--IQLLGEITTSQHTFQRGKRAMKVSGSQSCSSHNQCNRGQCCAFSFGRKICKDSQKTRKLGET
Anjap_PCNP_17 1 MASFIATTAVFISVMTFVLQLVSSIPIEVNGGGDGEEYTQLFGEITT-HDTFQRGKRAMKVSSSRSCSSNADCRRDQCCAFDFGRKVCKASQRVRNLGET
consensus *** *.**** ** ***** ***** * ** ** .. ** *****. ************ * **** * * ***** **** ** ** * ****

Amed_PCNP_17 98 CSIFDVHKYLDLNDLTRSFTTCPLVCRKGLRCRATQ---GKSRKRIAVCSRR
Anjap_PCNP_17 100 CSFVDIHKYLDLHDLTRSFTRCPMICKEGLRCWRTHERYSKSNTRKSVCRR-
consensus ** * ****** ******* ** * **** * ... ** * ** *.**
